# Supplementary material for: Peptide-conjugated antimiRs improve myotonic dystrophy type 1 phenotypes by promoting endogenous MBNL1 expression
Source: Mol Ther Nucleic Acids. 2023 Sep 5;34:102024. doi: 10.1016/j.omtn.2023.09.001 (PMC10514136; doi:10.1016/j.omtn.2023.09.001)
Supplement: Supplementary file 1 — Document S1. Figures S1–S10 and Tables S1, S2, S5, and S7 [file mmc1.pdf]

OMTN, Volume *34*

Supplemental information

Peptide-conjugated antimiRs improve myotonic dystrophy type 1 phenotypes by promoting endogenous MBNL1 expression

Irene González-Martínez, Estefanía Cerro-Herreros, Nerea Moreno, Andrea García-Rey, Jorge Espinosa-Espinosa, Marc Carrascosa-Sàez, Diego Piqueras-Losilla, Andrey Arzumanov, David Seoane-Miraz, Yahya Jad, Richard Raz, Matthew J. Wood, Miguel A. Varela, Beatriz Llamusí, and Rubén Artero

Supplemental Information

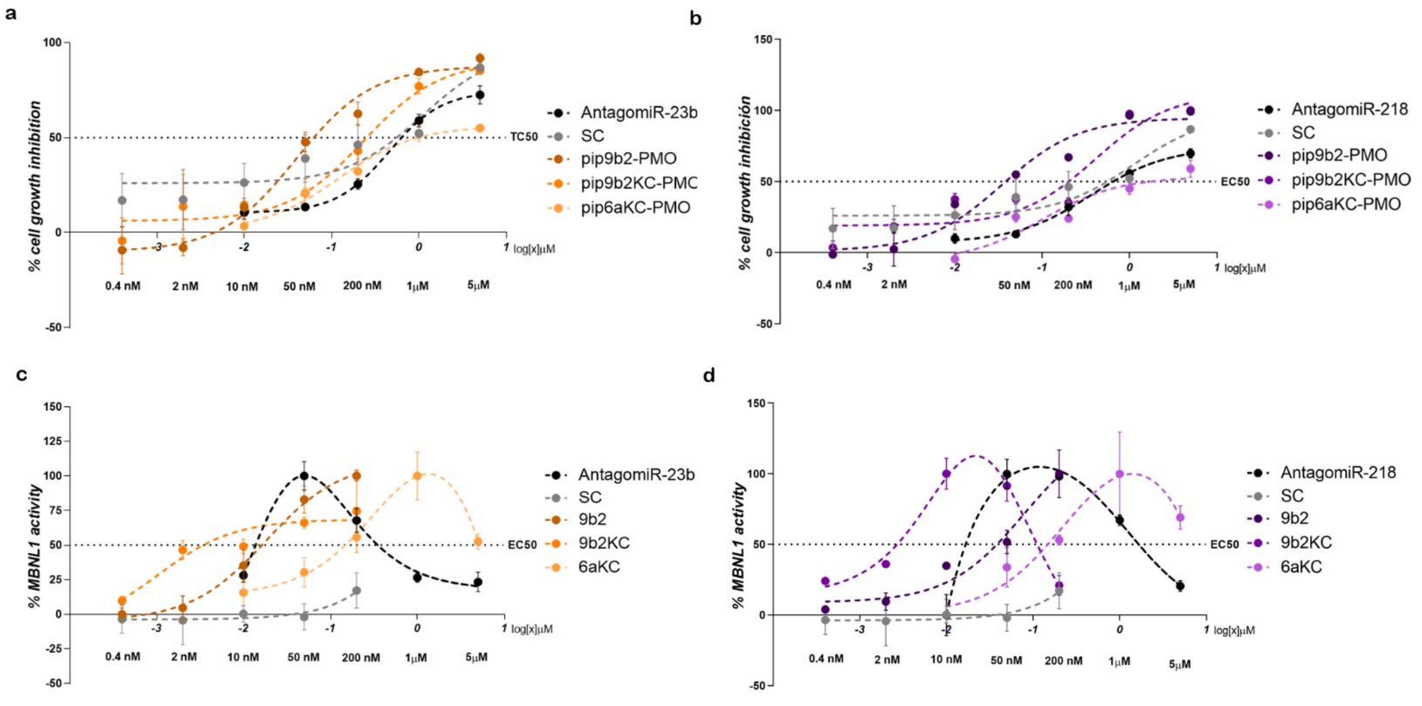

Figure S1 CPP-PMO antimiRs promote MBNL1 expression in DM1 cells. (**a**, **b**) Percentage of cell growth inhibition of DM1 cells after lipofection at different concentrations with ONs against (**a**) miR-23b and (**b**) miR-218. Dotted lines indicate TC_{50} value. (**c**, **d**) Percentage of MBNL1 expression increase (100% equals maximum increase) in DM1 cells after lipofection at the indicated concentrations with ONs against (**c**) miR-23b and (**d**) miR-218. Dotted lines indicate EC_{50} value. Error bars indicate mean ± SEM.

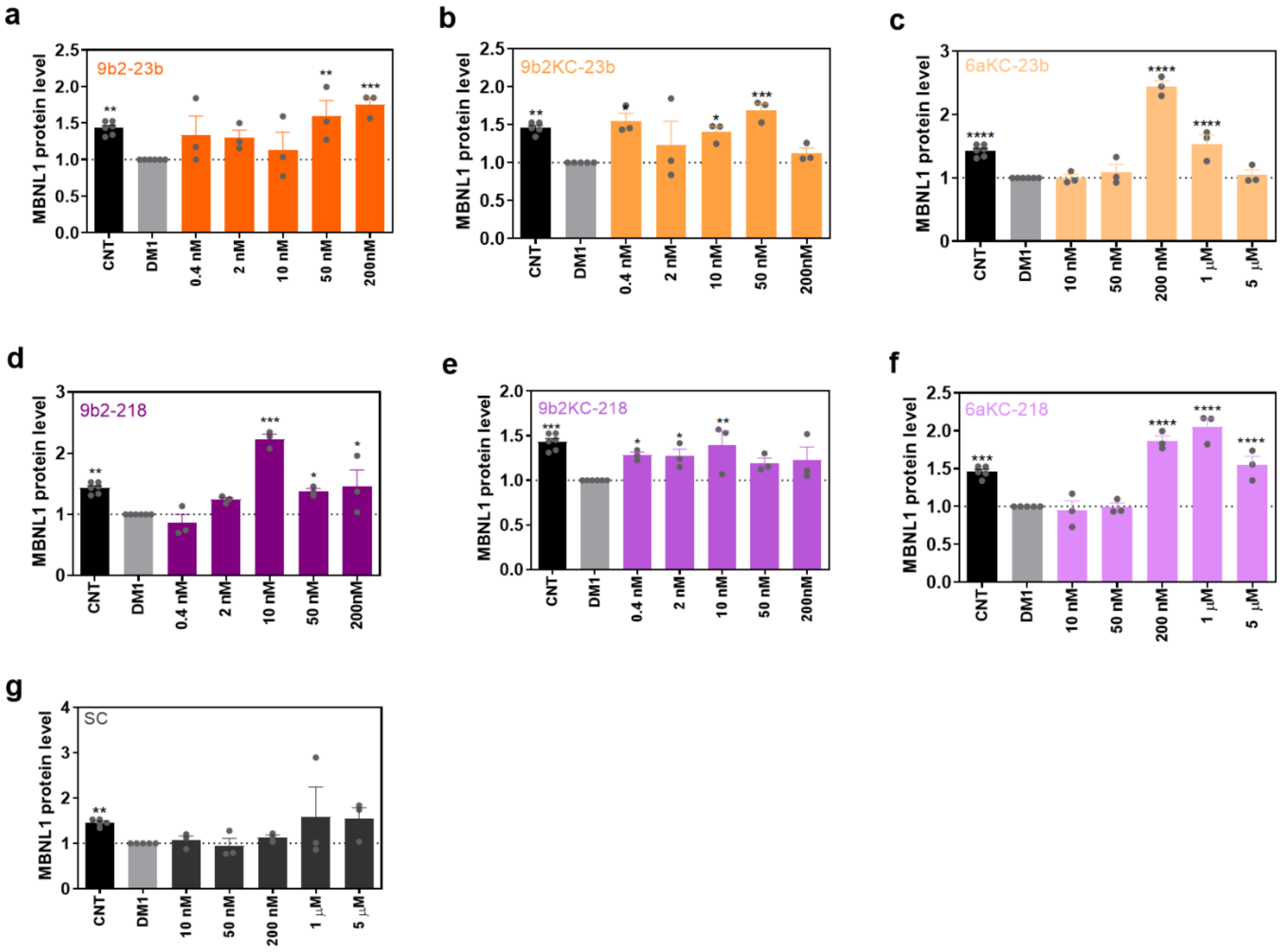

Figure S2 Activity effect of CPP-PMO antimiRs in DM cells by Gymnotic Delivery. Data shows the quantification by QDB of MBNL1 protein levels after treatment with different ONs in DM1 cells at the indicated concentrations: (**a**) 9b2-23b, (**b**) 9b2KC-23b, (**c**) 6aKC-23b, (**d**) 9b2-218, (**e**) 9b2KC-218, (**f**) 6aKC-218 and (**g**) scrambled control (SC). Black bars represent MBNL1 levels of unaffected control (CNT) myotubes and light grey bars represent MBNL1 levels in DM1 mock-treated cells. Dotted line indicates MBNL1 levels in DM1 cells without treatment. *: $p<0.05$, **: $p<0.01$, ***: $p<0.001$ according to one-way ANOVA compared to DM1 non-treated cells. Each concentration was tested in triplicate. Individual values are indicated as datapoints. Error bars indicate mean ± SEM.

a

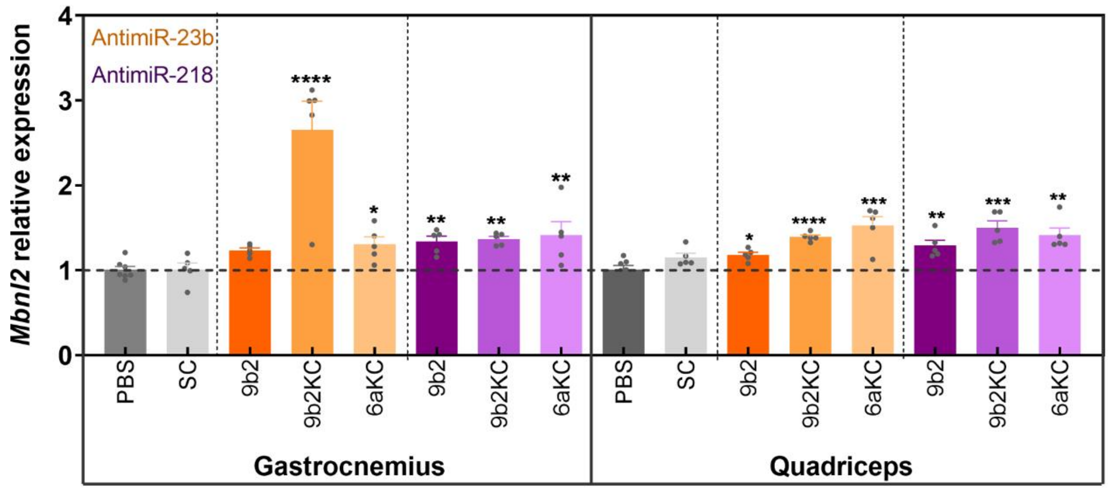

b

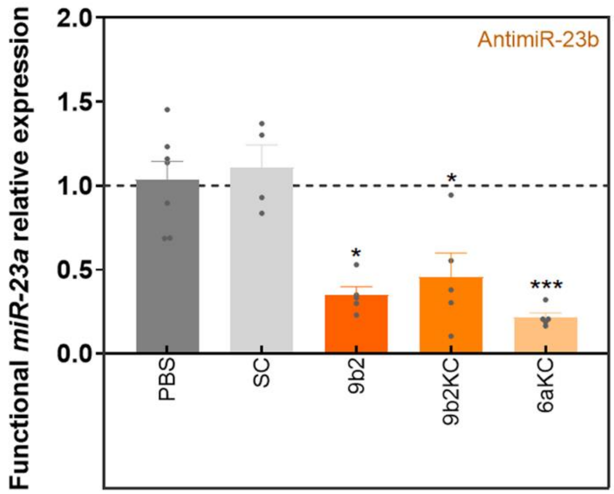

microRNA	Sequence	Max Score	Query Cover	E Value	Percent Identity	Accession Length
hsa-miR-23b-3p	AUCACAUUGCCAGGGAUUACCAC	-	-	-	-	23
hsa-miR-23a-3p	AUCACAUUGCCAGGGAUUUCC	36.2	78%	4.00E-09	87%	21

Figure S3 Administration of CPP-PMO antimiRs increases *Mbnl2* and decreases *miR-23a* expression in HSA^{LR} mice. After 45 days of treatment with CPP-PMOs, gastrocnemius and quadriceps muscles were dissected to measure (**a**) *Mbnl2* transcript levels relative to *Gapdh* endogenous control and (**b**) *miR-23a* functional levels relative to U1 and U6 snRNA endogenous controls. An alignment of miR-23b and miR-23a is shown. Statistical comparisons were performed in each case against PBS-treated *HSA^{LR}* values (indicated by a black dashed line) with Student's

t-tests. *: $p<0.05$, **: $p<0.01$, ***: $p<0.001$, ****: $p<0.0001$. PBS n=7, SC n=5, 9b2-218 n=5, 9b2-23b n=5, 9b2KC-218 n=5, 9b2KC-218 n=5, 6aKC-218 n=5, and 6aKC-23b n=5. Individual values are indicated as datapoints. Error bars indicate mean ± SEM.

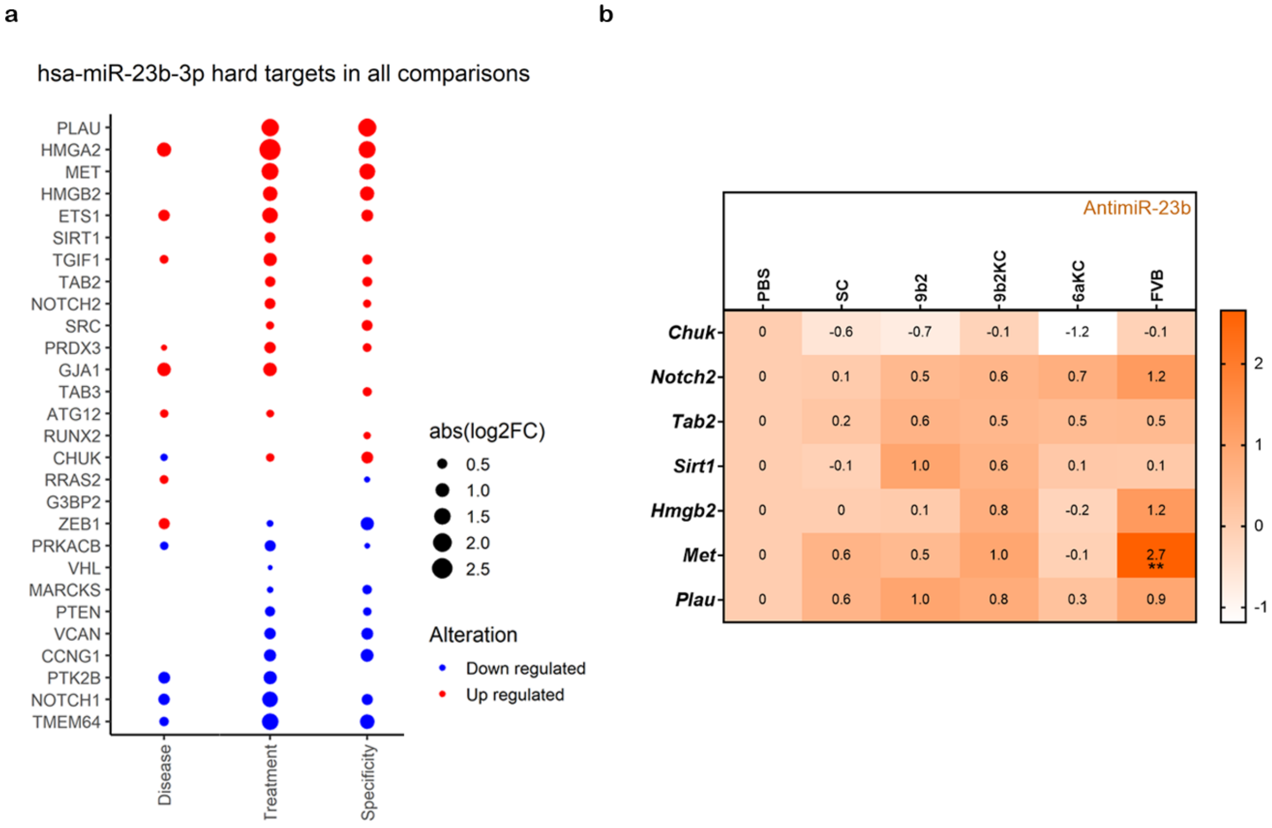

Figure S4 Assessment of effects by miR-23b blockers. (**a**) Effects on miR-23b target transcripts that previous antagomiR-23b manages to activate (red dots) or repress (blue dots). The size of each dot represents the log2 fold change of the expression of the indicated gene for the comparisons shown: DM1 untreated versus CNT healthy cells (Disease), antagomiR-23b-treated DM1 versus CNT healthy cells (Treatment), antagomiR-23b-treated DM1 versus DM1 untreated cells (Specificity). (**b**) Expression of selected genes (log2 fold change) in quadriceps muscles of CPP-PMO antimiRs-23b treated mice. Data were analysed by one-way ANOVA test compared to PBS-treated *HSA*^{LR} mice. **: $p<0.01$.

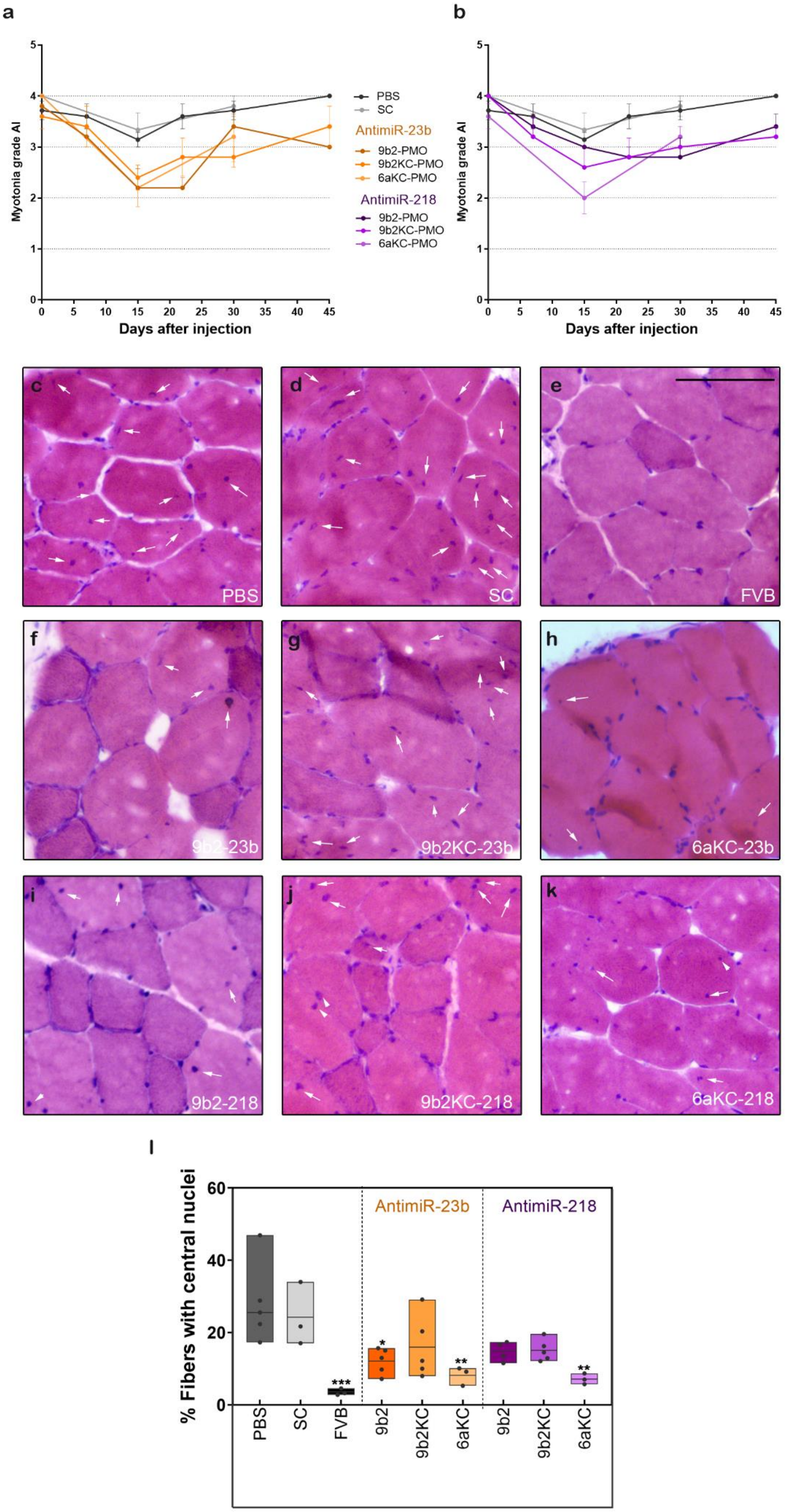
a
b
Myotonia grade AI
Days after injection
PBS
SC
AntimiR-23b
9b2-PMO
9b2KC-PMO
6aKC-PMO
AntimiR-218
9b2-PMO
9b2KC-PMO
6aKC-PMO
c PBS
d SC
e FVB
f 9b2-23b
g 9b2KC-23b
h 6aKC-23b
i 9b2-218
j 9b2KC-218
k 6aKC-218
l
% Fibers with central nuclei
AntimiR-23b
AntimiR-218
PBS
SC
FVB
9b2
9b2KC
6aKC
9b2
9b2KC
6aKC

Figure S5 CPP-PMOs antimiRs rescue functional and histological alterations in HSA^{LR} mice. (**a**,**b**) Myotonia grade was measured before injection, at the indicated intermediate time points, and immediately before sacrifice. (**c**-**k**) Representative images of hematoxylin and eosin staining of gastrocnemius muscle slices from each treatment group. White arrows indicate examples of centrally located nuclei in muscle fibres. (**l**) Quantification of the percentage of muscle fibers with central nuclei in gastrocnemius. Individual values are indicated as datapoints. Error bars indicate mean ± SEM. Data were analyzed by ANOVA one-way test compared to PBS-treated *HSA^{LR}* mice. *: p<0.05, **: p<0.01, ***: p<0.001. PBS n=7, SC n=5, 9b2-218 n=5, 9b2-23b n=5, 9b2KC-218 n=5, 9b2KC-218 n=5, 6aKC-218 n=5, and 6aKC-23b n=5.

A

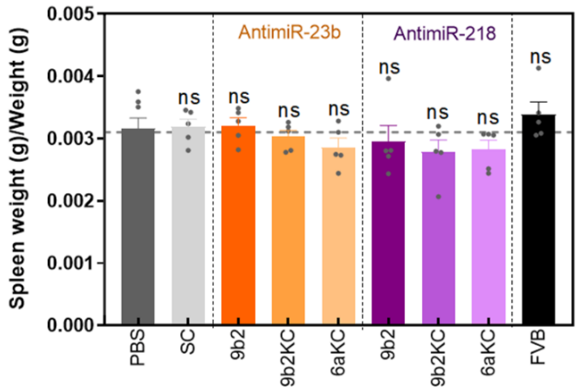
Spleen weight (g)/Weight (g)
0.005
0.004
0.003
0.002
0.001
0.000
AntimiR-23b
AntimiR-218
ns
PBS
SC
9b2
9b2KC
6aKC
9b2
9b2KC
6aKC
FVB

B

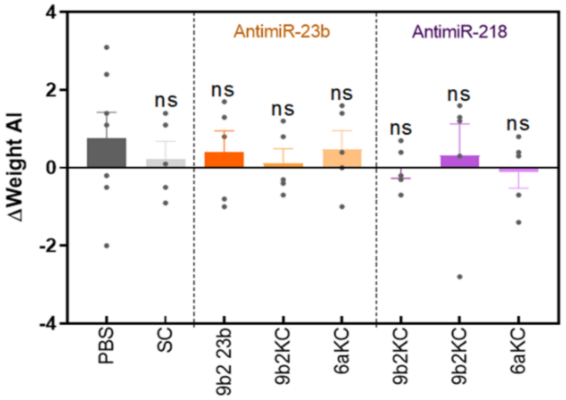
ΔWeight AI
4
2
0
-2
-4
AntimiR-23b
AntimiR-218
ns
PBS
SC
9b2 23b
9b2KC
6aKC
9b2KC
9b2KC
6aKC

C

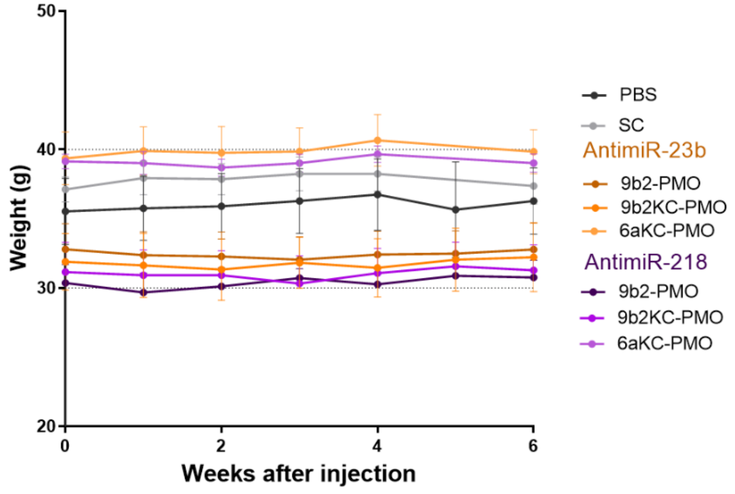
Weight (g)
50
40
30
20
0
2
4
6
Weeks after injection
PBS
SC
AntimiR-23b
9b2-PMO
9b2KC-PMO
6aKC-PMO
AntimiR-218
9b2-PMO
9b2KC-PMO
6aKC-PMO

Figure S6 Potential deleterious or toxic effects in response to CPP-PMOS in HSA^{LR} mice. Mouse relative (**a**) spleen weight or (**b**) weight after treatment with PBS, SC or different CPP-PMOs. (**c**) Evolution of weight of each mouse throughout the entire experiment. Statistical analysis (one-way ANOVA) was performed compared to PBS-treated HSA^{LR} controls. Individual values are indicated as datapoints. Error bars indicate mean ± SEM. ns: not statistically significant.

a

b

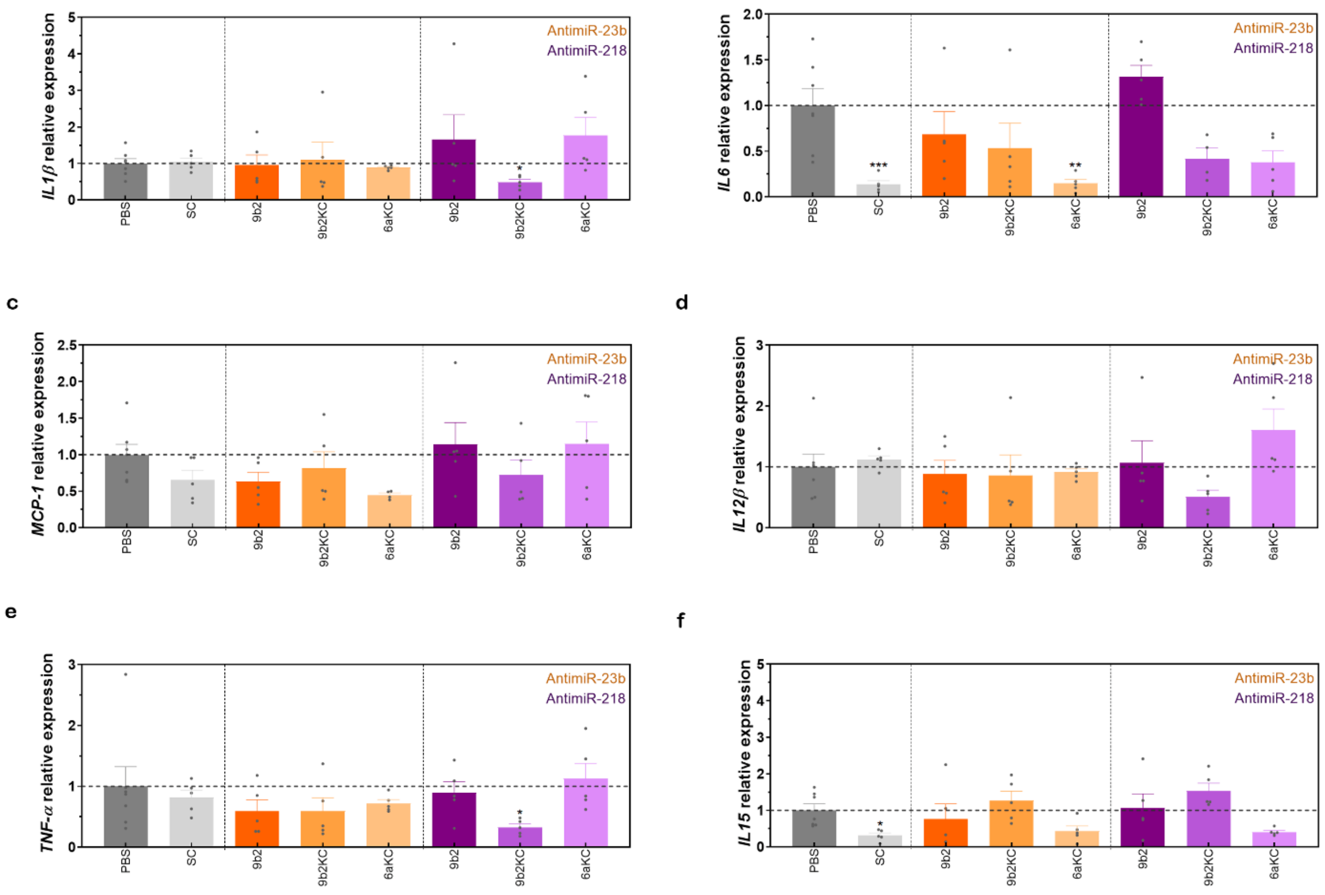

g

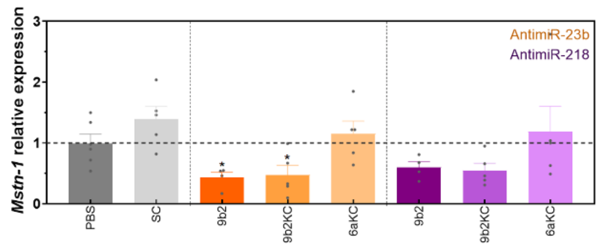

h

Qd	IL1β	IL6	MCP-1	IL12β	TNF-α	IL15	Mstn-1
SC	-	***	-	-	-	*	-
9b2-23b	-	-	-	-	-	-	*
9b2KC-23b	-	-	-	-	-	-	*
6aKC-23b	-	**	-	-	-	-	-
9b2-218	-	-	-	-	-	-	-
9b2KC-218	*	-	-	-	*	-	-
6aKC-218	-	-	-	-	-	-	-

Figure S7 CPP-PMOs antimiRs do not activate an immune response in the muscles of treated mice. Quantification by RT-qPCR of the cytokines (**a**) IL1β, (**b**) IL6, (**c**) MCP-1, (**d**) IL12β, (**e**) TNF-α, and (**f**) IL15, and **g**) muscle regeneration marker Mstn-1 in quadriceps muscles of *HSA*^{LR} mice treated with the designated treatment. (h) Summary table of significant changes observed. - indicates no significant change. All comparisons were to PBS-treated (indicated by a black dashed

line) mice and used ANOVA or Kruskal-Wallis test when necessary. All significant differences were towards reduction in treated muscles vs. untreated. *: $p<0.05$, **: $p<0.01$, ***: $p<0.001$.

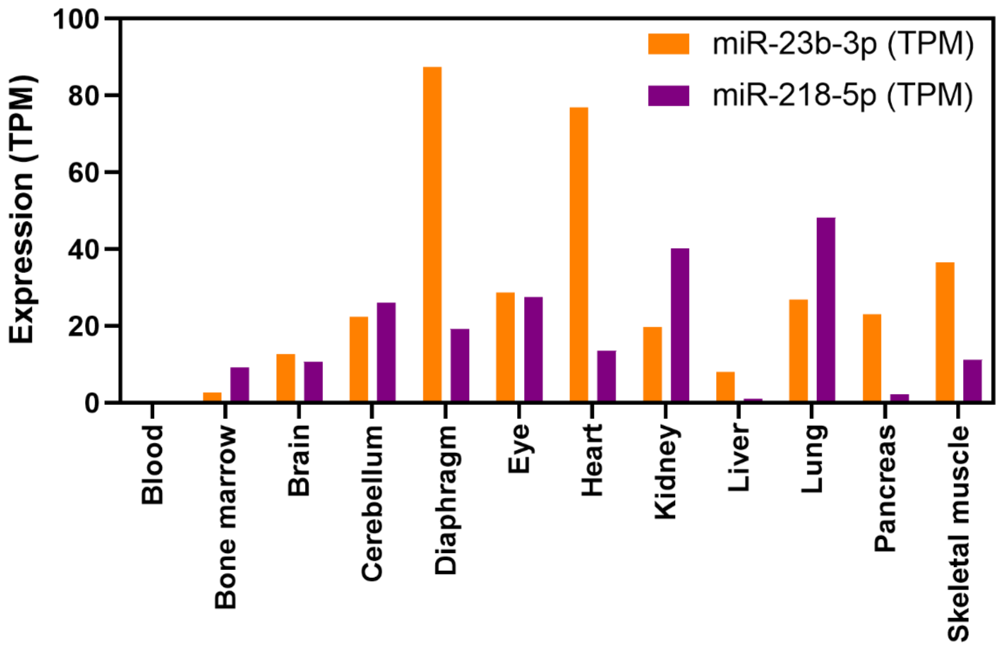

Figure S8 Expression of miR-23b and miR-218 in multiples tissues. Cap analysis gene expression data was retrieved from the ZENBU database (https://fantom.gsc.riken.jp/zenbu/) to prepare this figure. TPM: transcript per million.

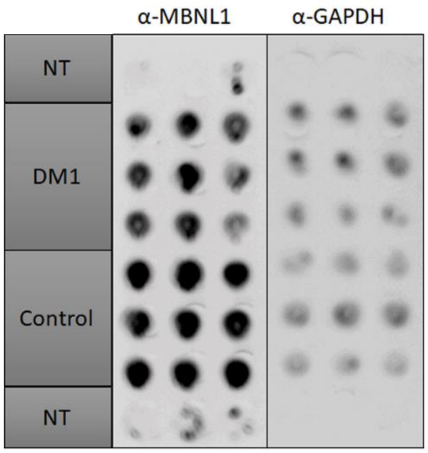

	MBNL1			GAPDH		
	1	2	3	4	5	6
Negative	2815	3097	1552	1575	1236	1395
	25462	35155	39750	8125	8334	6242
DM1	29254	51556	24689	10607	10526	9047
	29965	41118	20251	8708	7834	7103
	45666	59067	44824	10384	8918	7400
CNT	39675	48803	43641	11806	8157	9579
	47048	50495	54247	11034	9471	9794
Negative	2299	1104	1772	3260	2277	2497

Figure S9 Validation of QDB for in vitro studies. Dot blot images by ImageQuant of CNT and DM1 cells measuring MBNL1 and endogenous control GAPDH. The table shows readings of each dot obtained by Tecan plate reader.

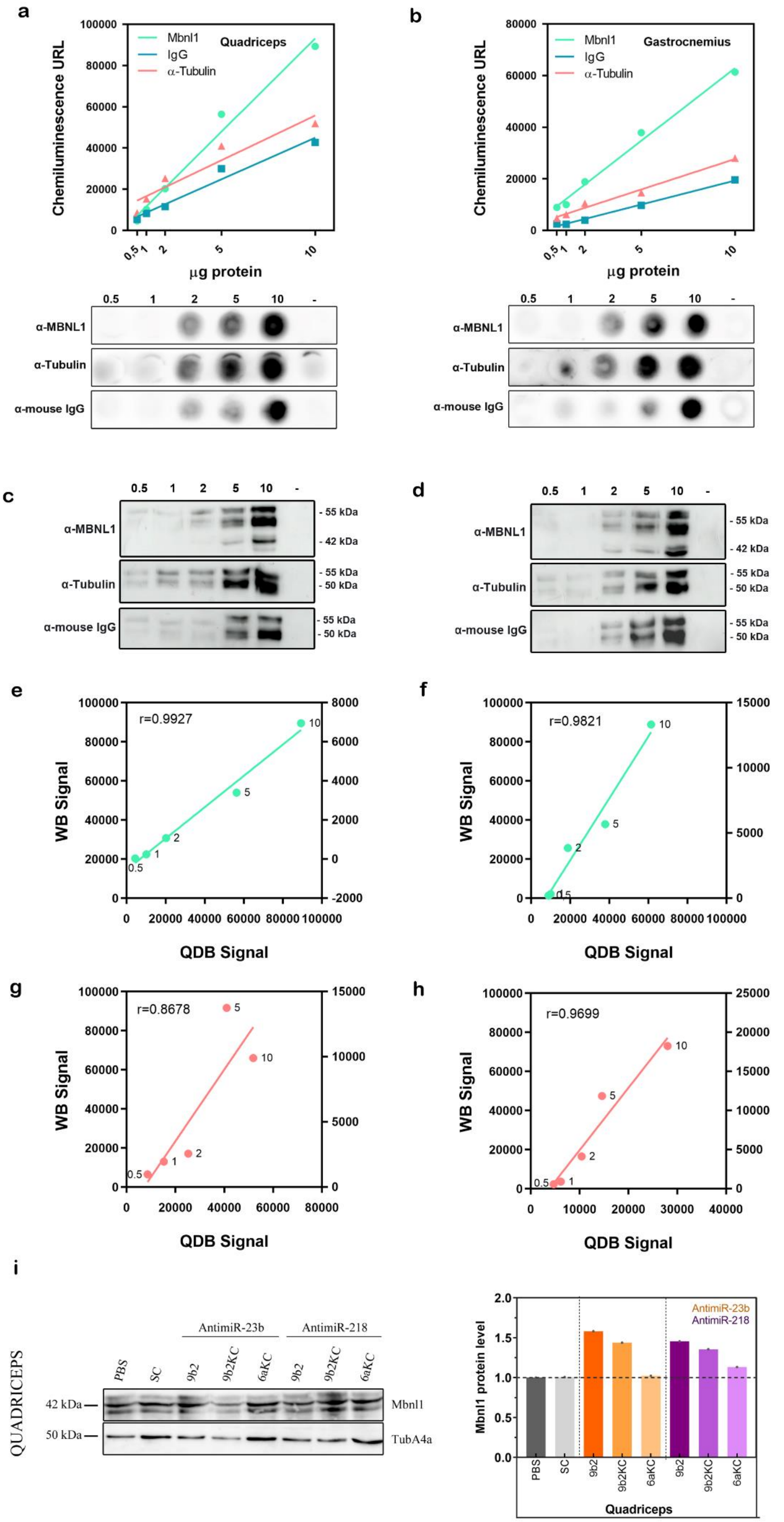
a
b
Chemiluminescence URL
Mbnl1
IgG
α-Tubulin
Quadriceps
Gastrocnemius
µg protein
0.5
1
2
5
10
-
α-MBNL1
α-Tubulin
α-mouse IgG
c
d
- 55 kDa
- 42 kDa
- 50 kDa
e
f
g
h
r=0.9927
r=0.9821
r=0.8678
r=0.9699
WB Signal
QDB Signal
i
QUADRICEPS
AntimiR-23b
AntimiR-218
PBS
SC
9b2
9b2KC
6aKC
42 kDa
50 kDa
Mbnl1
TubA4a
Mbnl1 protein level
Quadriceps

Figure S10 Optimization and validation of the QDB technique for quantification of Mbnl1 in murine muscle tissue. (**a**, **b**) Evaluation of specificity and lineal range of α-MBNL1, α-Tubulin and α-mouse-IgG antibodies in QDB using five protein inputs (0.5, 1, 2, 5 and 10 µg) of mouse muscle tissues samples. Representative images of QDB signal are shown underneath the curves, indicating the corresponding protein input. (**c**, **d**) Evaluation of the specificity of αMBNL1, αTubulin, and αmouseIgG antibodies in Western blot (WB) with mouse muscle samples in the same range of protein inputs. (**e**-**h**) Correlation between QDB and WB signal for (**e**, **f**) α-MBNL1 and (**g**, **h**) α-Tubulin antibodies in (**e**, **g**) quadriceps and (**f**, **h**) gastrocnemius muscles. (**i**) Representative Western blot of pools of samples from each treatment in quadriceps. -: negative control; r: Pearson coefficient of correlation.

Table S1 Top similarities for antimiR-23b and –218 sequences in human RNAs.

antimiR-23b				
Genomic position	Gen	Identity (%)	Overlap (bp)	Number of matches (bp)
chr9: 95085264-95085285	MIR-23B	100	21	21
chr9: 95085264-95085285	AOPEP	100	21	21
chr19: 13836594-13836615	MIR-23A	95.24	20	20
chrX: 20017101-20017122	MAP7D2	95.24	20	20
**antimiR-218**				
Genomic position	Gen	Identity (%)	Overlap (bp)	Number of matches (bp)
chr4:20528298-20528319	MIR-218-1	100	21	21
chr4:20528298-20528319	SLIT2	100	21	21
chr5:168768210-168768231	MIR-218-2	100	21	21
chr5:168768210-168768231	SLIT3	100	21	21
chr4:95432770-95432791	UNC5C	90.48	19	19
chr8:99669547-99669568	VPS13B	90.48	19	19

Table S2 Percentage spliced-in (PSI) of Nfix, Clcn1, and Atp2a1 of the indicated muscle.

		PSI					
Mice	Treatment	*Nfix* Gast	*Nfix* Quads	*Clcn1* Gast	*Clcn1* Quads	*Atp2a1* Gast	*Atp2a1* Quads
1990-1	HSA^{LR}-PBS	64.24	53.97	27.00	36.55	15.30	27.20
1995-1		55.56	71.29	24.30	30.28	30.33	21.37
1996-1		54.67	53.21	34.05	30.89	22.57	13.71
1996-2		66.55	46.33	31.36	36.72	7.55	37.47
2130-1		76.55	65.44	47.52	31.25	2.48	16.44
2710-4		67.04	77.90	28.18	33.10	12.30	18.78
2657-4		70.82	65.53	27.60	29.53	12.53	19.77
2710-1	Scramble	62.98	80.52	26.86	38.65	15.65	7.50
2657-1		76.46	72.49	36.37	28.91	6.44	18.65
2817-1		52.74	60.30	12.37	16.71	27.49	34.43
2816-1		55.70	66.59	14.67	28.33	24.15	18.71
2713-1		81.00	72.38	42.81	34.05	5.60	17.01
1990-2	9b2-218	58.31	10.51	18.06	24.88	31.61	99.28
1995-2		69.09	35.59	25.23	1.85	14.72	71.69
2032-1		29.97	30.95	5.28	4.09	85.58	99.29
2130-2		59.09	43.26	30.49	13.35	28.83	50.19
2131-1		66.06	15.39	30.67	26.96	19.19	99.72
1990-4	9b2KC-218	66.68	67.46	22.57	22.73	35.28	25.20
1995-4		62.07	21.60	29.41	2.19	12.53	99.70
2087-1		66.91	48.85	32.73	27.06	17.65	25.98
2130-4		60.18	51.19	22.25	25.84	19.44	29.31
2131-3		67.84	51.94	31.59	30.53	12.90	30.83
2710-3	6aKC-218	68.36	62.29	24.01	27.67	12.62	26.51
2657-3		74.79	66.72	28.56	33.68	14.70	21.08
2817-3		70.85	52.44	28.37	24.84	11.51	26.96
2816-3		73.58	52.77	38.26	25.74	10.78	31.16
2713-3		51.68	89.96	18.26	41.25	31.20	9.84
1990-3	9b2-23b	55.08	54.78	26.86	24.88	23.52	36.45
1995-3		47.66	23.44	11.37	1.85	52.69	98.27
2032-2		63.11	15.30	26.85	0.61	18.37	99.53
2130-3		66.38	4.40	32.69	13.35	16.01	75.32
2131-2		63.52	48.44	33.28	26.96	11.16	25.61
1990-5	9b2KC-23b	45.55	56.90	15.58	26.94	42.67	39.24
2087-2		57.17	56.46	38.58	29.66	11.22	37.72
2130-5		53.38	54.10	18.78	22.47	31.29	33.71
2032-3		57.04	66.58	26.33	19.41	33.92	28.53
2131-4		62.73	51.62	30.11	27.99	10.51	24.15
2710-2	6aKC-23b	63.59	62.29	29.03	28.33	14.74	33.87
2657-2		69.22	53.11	31.54	24.44	14.22	29.81
2817-2		47.55	56.32	15.22	30.63	26.08	23.38
2816-2		61.79	64.55	18.35	35.11	16.73	17.76
2713-2		62.43	58.42	23.17	37.69	17.96	24.03

Table S3 Serum biochemistry profile of different mice treated with CPP-PMOs antimiRs. Mean ± SEM is shown for each biomarker in the indicated treatment group. Statistical test compares CPP-PMO-treated *HSA*^{LR} mice with FVB controls.

Table S4 White blood cells (WBC) count for each treatment group and a compositional data dendrogram. Mean ± SEM is shown for each cell count of the indicated groups. The statistical test compares CPP-PMO-treated mice with FVB controls. In all cases treatments refer to *HSA*^{LR} mice treated with each CPP-PMO, Scramble or PBS. FVB controls were mock-treated with PBS. *$p<0.05$.

Table S5 Sequences of primers used for RT-qPCR of off-targets, interleukins and muscle regeneration marker Mstn-1 and RT-PCR of splicing events.

Gene	Forward primer	Reverse primer	Exon
**CHUK**	TCGGAAACCAGCCTCTCAGTGT	CTTCTGGATGCAAATGGTCCTTC	-
**SRC**	GTTGCTTCGGAGAGGTGTGGAT	CACCAGTTTCTCGTGCCTCAGT	-
**NOTCH2**	CCACCTGCAATGACTTCATCGG	TCGATGCAGGTGCCTCCATTCT	-
**TAB2**	CATTCAGCATCTCACAGACCCG	CTTTGAAGCCGTTCCATCCTGG	-
**SIRT1**	GGAGCAGATTAGTAAGCGGCTTG	GTTACTGCCACAGGAACTAGAGG	-
**HMGB2**	GATGTGGTCTGAGCAATCTGCC	CCTGCTTCACTTTTGCCCTTGG	-
**MET**	GTTCTGCTTGGCAACGAGAGCT	GGAGAATGCACTGTATTGCGTCG	-
**PLAU**	AGAAGCGACCCTGGTGCTATGT	CCACACTGGAAGCCTTGTTGGT	-
**IKBKB**	GCAGACTGACATTGTGGACCTG	ATCTCCTGGCTGTCACCTTCTG	-
**RPS6KA3**	TAACCGCAGAGGTCACACTCAG	CTCAGAAACTGTGGCATCCCGA	-
**OTUD7B**	GGAGGTGAAGTTACATCTGCTGC	TCAGGAGTGGACCTGGGTTCAT	-
**HMOX1**	CACTCTGGAGATGACACCTGAG	GTGTTCCTCTGTCAGCATCACC	-
**RET**	TCAGTACACGGTGGTAGCCACT	CGCCTCTTGTTTACTGCACAGG	-
**GLI2**	ACACTGTGGAGGACTGCCTACA	GGCATCTCCATGCCACTGTCAT	-
**CDH2**	CCTCCAGAGTTTACTGCCATGAC	CCACCACTGATTCTGTATGCCG	-
**IL1β**	TGGACCTTCCAGGATGAGGACA	GTTCATCTCGGAGCCTGTAGTG	-
**IL6**	TACCACTTCACAAGTCGGAGGC	CTGCAAGTGCATCATCGTTGTTC	-
**MCP-1**	GCTACAAGAGGATCACCAGCAG	GTCTGGACCCATTCCTTCTTGG	-
**TNF-α**	GGTGCCTATGTCTCAGCCTCTT	GCCATAGAACTGATGAGAGGGAG	-
**IL12β**	TTGAACTGGCGTTGGAAGCACG	CCACCTGTGAGTTCTTCAAAGGC	-
**Mstn-1**	AACCTTCCCAGGACCAGGAGAA	GGCTTCAAAATCGACCGTGAGG	-
**IL15**	GTAGGTCTCCCTAAAACAGAGGC	TCCAGGAGAAAGCAGTTCATTGC	-
**Nfix**	TCGACGACAGTGAGATGGAG	CAAACTCCTTCAGCGAGTCC	7
**Atp2a1**	GCTCATGGTCCTCAAGATCTCAC	GGGTCAGTGCCTCAGCTTTG	22
**Clcn1**	GTCCTCAGCAAGTTTATGTCC	GAATCCTCGCCAGTAATTCC	7a
**Gapdh**	ATCAACGGGAAGCCCATCAC	CTTCCACAATGCCAAAGTTGT	-

Table S6 Data of individual mice treated with CPP-PMOs antimiRs. Data on the following parameters: age, qRT-PCR, QDB, relative spleen weight, myotonia grade, and weight at different time points. Outliers are indicated in blue, in italics, and with an asterisk.

Table S7 Concentration of the CPP-PMOs in six different tissues: kidney, liver, heart, gastrocnemius, quadriceps, and brain.

		CPP-PMO concentration (pM)					
Mice	Treatment	Kidney	Liver	Heart	Gastrocnemius	Quadriceps	Brain
1990-2	9b2-218	348919.38	11986.59	1995.51	428.98	1437.67	128.86
1995-2		726042.17	23535.44	1662.31	504.97	482.70	33.45
2032-1		303314.43	16959.56	1166.04	1534.26	1344.85	18.40
1990-4	9b2KC-218	1320762.73	13391.72	184.66	788.22	358.44	1017.83
1995-4		1409556.69	5588.09	108.67	726.85	37.03	6.68
2087-1		1455590.50	12456.78	1724.69	1065.08	1605.97	4.47
2710-3	6aKC-218	807303.00	6547.20	1672.26	-	-	-
2657-3		282189.80	6702.77	-	-	-	-
2817-3		288519.00	6452.86	-	-	180.55	-
2816-3		763252.10	11820.61	220.63	3105.34	56.37	-
2713-3		163863.50	1738.96	8031.17	4126.64	3449.73	5.54
1990-3	9b2-23b	417329.90	13935.38	70.02	96.96	237.24	3.33
1995-3		596014.00	16995.86	540.73	1173.05	664.60	84.29
2032-2		445161.13	14705.39	458.72	672.99	444.65	5.89
1990-5	9b2KC-23b	404860.30	27707.42	2.97	0.01	0.82	0.87
2023-3		1672927.68	93287.30	105.40	3.32	127.62	17.78
2087-2		2213436.59	113314.70	256.63	2.34	457.08	0.01
2710-2	6aKC-23b	174345.23	9209.58	1508.23	199.58	1.72	4.02
2657-2		230939.23	8623.71	1701.45	93.86	72.30	0.83
2817-2		110334.18	4615.98	1789.14	1846.67	93.71	9.49
2816-2		239168.85	10333.80	954.01	1209.72	165.91	11.43
2713-2		16740.44	76.40	-	472.26	28.58	25.59
