# Supplementary material for: Peptide-conjugated antimiRs improve myotonic dystrophy type 1 phenotypes by promoting endogenous MBNL1 expression
Source: Mol Ther Nucleic Acids. 2023 Sep 5;34:102024. doi: 10.1016/j.omtn.2023.09.001 (PMC10514136; doi:10.1016/j.omtn.2023.09.001)
Supplement: Document S2. Article plus supplemental information [file mmc5.pdf]

Molecular Therapy
Nucleic Acids
Original Article

Peptide-conjugated antimiRs improve myotonic dystrophy type 1 phenotypes by promoting endogenous MBNL1 expression

Irene González-Martínez,[1,2] Estefanía Cerro-Herreros,[1,2,6] Nerea Moreno,[1,2] Andrea García-Rey,[1,2] Jorge Espinosa-Espinosa,[1,2,3] Marc Carrascosa-Sàez,[1] Diego Piqueras-Losilla,[1] Andrey Arzumanov,[4,5] David Seoane-Miraz,[4,5] Yahya Jad,[4,5] Richard Raz,[4,5] Matthew J. Wood,[4,5] Miguel A. Varela,[4,5] Beatriz Llamusí,[1,2,6] and Rubén Artero[1,2]

[1]University Research Institute for Biotechnology and Biomedicine (BIOTECMED), Universidad de Valencia, Valencia, Spain; [2]Translational Genomics Group, INCLIVA Biomedical Research Institute, Avenue Menéndez Pelayo 4 acc, 46010 Valencia, Spain; [3]Group of Emerging and Neglected Diseases, Ecoepidemiology and Biodiversity, Health Sciences Faculty, Universidad Internacional SEK, Quito 170521, Ecuador; [4]Department of Paediatrics, Institute of Developmental and Regenerative Medicine (IDRM), University of Oxford, Roosevelt Dr, Oxford OX3 7TY, UK; [5]MDUK Oxford Neuromuscular Centre, University of Oxford, Oxford, UK

Myotonic dystrophy type 1 (DM1) is a rare neuromuscular disease caused by a CTG repeat expansion in the *DMPK* gene that generates toxic RNA with a myriad of downstream alterations in RNA metabolism. A key consequence is the sequestration of alternative splicing regulatory proteins MBNL1/2 by expanded transcripts in the affected tissues. MBNL1/2 depletion interferes with a developmental alternative splicing switch that causes the expression of fetal isoforms in adults. Boosting the endogenous expression of MBNL proteins by inhibiting the natural translational repressors miR-23b and miR-218 has previously been shown to be a promising therapeutic approach. We designed antimiRs against both miRNAs with a phosphorodiamidate morpholino oligonucleotide (PMO) chemistry conjugated to cell-penetrating peptides (CPPs) to improve delivery to affected tissues. In DM1 cells, CPP-PMOs significantly increased MBNL1 levels. In some candidates, this was achieved using concentrations less than two orders of magnitude below the median toxic concentration, with up to 5.38-fold better therapeutic window than previous antagomiRs. In *HSA^{LR}* mice, intravenous injections of CPP-PMOs improve molecular, histopathological, and functional phenotypes, without signs of toxicity. Our findings place CPP-PMOs as promising antimiR candidates to overcome the treatment delivery challenge in DM1 therapy.

INTRODUCTION

Myotonic dystrophy type 1 (DM1) is a rare neuromuscular disease originating from an abnormal expansion of a CTG microsatellite in the 3′ untranslated region of the *DM1 protein kinase* (*DMPK*) gene.[1] Expansion-bearing *DMPK* transcripts act as toxic RNAs by affecting key RNA processing mechanisms that impinge on intracellular cell signaling pathways.[2] Given the widespread ubiquitous expression of the *DMPK* gene, DM1 is characterized by a multisystem collection of symptoms. However, some of the most disabling manifestations of the disease emerge from muscle tissue alterations, particularly cardiac conduction problems, skeletal muscle atrophy, and myotonia.[3] One of the chief pathogenic roles of mutant *DMPK* transcripts is their ability to sequester and deplete muscleblind-like (MBNL) proteins, impairing their normal functions as regulators of alternative splicing and polyadenylation of pre-mRNAs.[4–6] Stress responses triggered by the toxic *DMPK* RNA also cause the stabilization/activation of MBNL antagonists, such as CELF1,[7] Staufen1,[8] and hnRNPA1.[9] Nevertheless, dysregulated splicing in DM1, which has been shown to produce many of the main features of the disease, can be attributed to functional loss of MBNL proteins.[10,11] Out of three human MBNL paralogs, skeletal muscles mainly express MBNL1, while MBNL2 seems to be more prevalent in the central nervous system (CNS), and MBNL3 serves functions related to regeneration, placental growth, and aging.[12–16] There is ample evidence supporting that sequestration by the CUG repeat expansions can be overcome by transgenic, viral, or endogenous overexpression of MBNL1, indicating that an increase in these proteins may be a suitable therapeutic strategy.[17–19] MBNL1 activity can also be promoted by inhibiting the regulatory miRNAs miR-23b and miR-218, which are naturally occurring translational repressors of *MBNL1* transcripts.[20] Synthetic oligonucleotides (ONs) can be designed to target and silence miRNAs. These molecules are usually called "antimiRs" or, more specifically, antagomiRs when conjugated to cholesterol.[21,22] AntagomiRs against miR-23b and miR-218 have been shown previously to rescue splicing alterations (so-called missplicing) in cell

Received 12 July 2022; accepted 1 September 2023;
https://doi.org/10.1016/j.omtn.2023.09.001.

[6]Present address: ARTHEx Biotech, Parque Científico de la Universidad de Valencia, Calle del Catedrático Agustín Escardino Benlloch, 9, 46980 Paterna, Valencia, Spain

Correspondence: Estefanía Cerro-Herreros, Department of Genetics-Biotecmed, Faculty of Biology, Campus of Burjasot Dr. Moliner, 50, 46100 Burjasot, Valencia, Spain.
E-mail: Estefania.cerro@uv.es

This is an open access article under the CC BY-NC-ND license (http://creativecommons.org/licenses/by-nc-nd/4.0/).

and mouse models of DM1, correcting functional and histopathological signs of the disease.[20,23,24]

The pharmacokinetic/pharmacodynamic (PK/PD) profile of ONs is highly dependent on the chemical modifications necessary to enhance their binding affinity to the target and protect them against nuclease degradation.[25] Once ONs reach the target cells, they are internalized by endocytic mechanisms.[26] Although some ON molecules will reach the cytosol over the course of vesicular trafficking, most of them accumulate in endo-lysosomal compartments over time, where they can reside for long periods due to their intrinsic resistance to endogenous nucleases.[27] This endosomal entrapment phenomenon is currently a major drawback to further improving treatment efficacy because it limits the exposure to the target. Several strategies have been explored to enhance the uptake of ON-based drugs by the skeletal musculature, including the conjugation to anti-transferrin receptor antibodies and cell-penetrating peptides (CPPs).[28] Among CPPs, short peptides (∼20 amino acids) containing two flanking regions enriched with cationic residues and a central hydrophobic core are particularly effective for delivering associated cargoes.[29,30] CPPs can also help to bypass endosomal entrapment by enhancing endosomal escape and increasing the therapeutic potential of ONs.[31]

Another unique feature of cationic CPPs is that they can penetrate hard-to-reach tissues such as skeletal musculature and the heart.[29] However, negatively charged ON molecules with natural phosphodiester backbone, or those with the widely used phosphorothioate linkage modification, are not suitable to be used conjugated with this type of CPPs due to aggregation problems caused by the electrostatic interactions between the positive charges of the peptide and the negative ones in the ON backbone.[32] In contrast, ONs with phosphorodiamidate morpholino oligonucleotide (PMO) chemistry are uncharged molecules in the physiological pH range, suitable for any CPP conjugation. In addition, their unnatural structure confers them with robust protection against nuclease degradation. Notably, several investigational PMOs and four drugs have reached market authorization, namely eteplirsen, golodirsen, vitolarsen, and casimersen.[33] In the context of DM1, a recent study using a CPP-conjugated PMO (designed to target nucleus-retained CUGexp transcripts) enhanced ON delivery to DM1 mouse skeletal muscles in comparison with the unconjugated version.[34]

Here, we report the design and testing of six CPP-conjugated PMO antimiRs (three against miR-23b and three against miR-218) in DM1 patient-derived myotubes and a DM1 mouse model. To our best knowledge, this is the first study of CPP conjugation as a delivery strategy for antimiR molecules. The CPPs tested in this study were designed with two flanking regions enriched with cationic amino acids (arginine [R] or lysine [K]) and a central hydrophobic core. 9b2 and 6aKC are R-rich peptides already shown to penetrate hard-to-reach tissues, delivering associated cargoes in neuromuscular diseases.[30,34,35] Since R-content increases delivery efficiency but can also lead to cell toxicity,[36] we designed a third CPP (9b2KC) in which several R residues were replaced with another basic amino acid: K. As positive controls, we used antagomiRs 23b and 218 since they have been previously shown to increase MBNL1 levels both *in vitro* and *in vivo*.[20] In cells, we identified CPP-antimiRs with up to 5.38-fold better therapeutic window than previous antagomiRs, defined as the ratio between the median toxic and active concentrations. We also compared classic transfection conditions to test ON compounds *in vitro* with the gymnosis method,[37] as the latter can be used to assess the natural uptake and intracellular trafficking routes of DM1 cells. *In vivo*, three intravenous injections of antimiRs over 45 days improved molecular, tissue architecture, and functional parameters related to DM1, with no signs of toxicity during the entire treatment period. This study suggests that a novel family of K-rich CPPs (9b2KC, 6aKC) can yield potent activity while keeping toxicity low when targeting miRNAs.

RESULTS

Transfection-mediated evaluation of CPP-PMO antimiRs in DM1 muscle cells

As a proof-of-concept, CPP-PMOs were first evaluated for their ability to increase MBNL1 levels (i.e., the expected activity of the antimiR) in a DM1 cell model developed by Arandel et al.[38] ONs were transfected at concentrations determined in previous pilot experiments (data not shown) to cover the entire optimal range of activity in each case (Table 1). Analyzing maximum activity (E_{max}) (Table 1; Figure 1) in MBNL1 expression compared with untreated (DM1) control cells, we observed that CPP-PMOs linked to 9b2 induced a ∼4-fold increase in MBNL1 at 200 nM concentration with both antimiR against miR-23b or against miR-218 (Figures 1A and 1D). By comparison, the AntagomiRs produced a ∼2-fold increase in MBNL1 levels at the same concentration (Figures 1G and 1H). In turn, CPP-PMOs linked to 6aKC reached their maximum activity at 1,000 nM concentration, inducing a ∼1.5-fold change for 6aKC-218 and a ∼2-fold change for 6aKC-23b (Figures 1C and 1F). CPP-PMOs linked to 9b2KC presented a different pattern of activity depending on the miRNA target. The activity of 9b2KC-23b peaked at ∼3-fold change when using a concentration of 200 nM (Figure 1B). In contrast, the maximum activity of 9b2KC-218 reached almost a ∼6-fold change at only 10 nM concentration (Figure 1E). Finally, as expected, a scrambled control (SC, an oligonucleotide with a random sequence that presents the same chemistry modifications as the rest) ON showed no ability to enhance MBNL1 expression at any concentration tested (Figure 1I).

Next, we performed a cell viability assay to obtain the mean toxic concentration (TC_{50}) of our ONs (Figures S1A and S1B). These data, together with the E_{max} and the median effective concentration (EC_{50}) (Figures S1C and S1D), were used to calculate the therapeutic index (T_{index}) of each compound (Table 1). T_{index} primarily indicates the window between two thresholds: the concentration at which half of the cells are viable and the concentration at which the compound reaches half the maximum activity (see materials and methods), which is a useful parameter to rank the conjugates. According to this parameter, the 9b2KC CPP provided the best results in conjugation with both antimiRs against miR-23b and miR-218, with a T_{index}

Table 1. AntagomiRs and CPP-PMOs used in the study

On	Sequence (5′ to 3′)	Concentration	EC_{50} (nM)	TC_{50} (nM)	E_{max}	T_{index}	ΔT_{index}
Scramble	TCTTACCTCAGTTACAATTTA-Pip9b2	10 nM, 50 nM, 200 nM, 1 µM, 5 µM	890.00	217.00	1.30	0.32	–
AntagomiR-23b	mG*mG*mUmAmAmUmCmCmCmUmGmGm CmAmAmUmGmU*mG*mA*mU*-chol	10 nM, 50 nM, 200 nM, 1 µM, 5 µM	27.80	805.80	2.59	75.08	1
9b2-23b	GGUAAUCCCUGGCAAUGUGAU-Pip9b2	0.4 nM, 2 nM, 10 nM, 50 nM, 200 nM, 1 µM, 5 µM	16.00	88.00	4.58	25.19	0.34
9b2KC-23b	GGUAAUCCCUGGCAAUGUGAU-Pip9b2KC	0.4 nM, 2 nM, 10 nM, 50 nM, 200 nM, 1 µM, 5 µM	2.00	256.00	3.16	404.48	5.38
6aKC-23b	GGUAAUCCCUGGCAAUGUGAU-Pip6aKC	10 nM, 50 nM, 200 nM, 1 µM, 5 µM	452.80	1711.00	2.06	7.78	0.10
AntagomiR-218	mA*mC*mAmUmGmGmUmUmAmGmAmUmCm AmAmGmCmA*mC*mA*mA*-chol	10 nM, 50 nM, 200 nM, 1 µM, 5 µM	22.4	848.0	1.87	70.81	1
9b2-218	ACAUGGUUAGAUCAAGCACAA-Pip9b2	0.4 nM, 2 nM, 10 nM, 50 nM, 200 nM, 1 µM, 5 µM	64.68	42.00	3.89	2.52	0.04
9b2KC-218	ACAUGGUUAGAUCAAGCACAA-Pip9b2KC	0.4 nM, 2 nM, 10 nM, 50 nM, 200 nM, 1 µM, 5 µM	2.58	88.26	5.80	198.07	2.80
6aKC-218	ACAUGGUUAGAUCAAGCACAA-Pip6aKC	10 nM, 50 nM, 200 nM, 1 µM, 5 µM	138.40	1809.00	1.74	22.78	0.32

The corresponding sequence and concentration range used in DM1 myotubes is indicated for each oligonucleotide (ON). For each ON, the following parameters were calculated (see description in materials and methods): EC_{50}, TC_{50}, E_{max} (maximum fold change), and T_{index} (therapeutic index). *Phosphorothioate linkages; m, 2′-O-methyl-modified phosphoramidites; chol, cholesterol groups. ΔT_{index} indicates the fold change in T_{index} relative to the values of the control antagomiRs reported in Cerro-Herreros et al.[20]

of 404.48 and 198.07, respectively (Figure S1; Table 1). In fact, 9b2KC-23b was the most efficient compound of all CPP-PMOs overall, showing 128-fold higher TC_{50} than EC_{50}. Interestingly, the T_{index} for the previously studied antagomiRs against miR-23b and miR-218 (under the same experimental conditions) were 75.08 and 70.81, respectively. The T_{index} values of the ONs linked to 9b2KC vs. 9b2 were significantly higher in 9b2KC (404.48 vs. 25.19 for miR-23b antimiRs and 198.07 vs. 2.52 for miR-218 antimiRs), indicating that the R to K change in the 9b2 peptide broadened the difference between median activity and toxicity. Finally, the T_{index} of PMOs linked to CPP 9b2KC was superior to those linked to 6aKC (404.48 vs. 7.78 for miR-23b antimiRs and 198.07 vs. 22.78 for miR-218 antimiRs).

Gymnotic delivery of CPP-PMO antimiRs in DM1 cells

Transfection reagents induce cellular uptake and endosomal escape of the cargo,[39] hence bypassing the natural uptake routes of eukaryotic cells. Before administering these compounds to mice, an additional *in vitro* test was carried out to determine whether CPP-PMOs could penetrate DM1 cells and reach their miRNA targets unassisted by transfection reagents, i.e., by gymnosis (Figures 1J and 1K). We carried out a gymnotic delivery experiment at the same doses used for transfection in DM1 cells (Figure S2). The most similar results to transfection assay were achieved with compounds linked to 6aKC peptides, achieving a ∼2-fold change for 6aKC-218 (at 1 µM concentration) and a ∼2.5-fold change for 6aKC-23b (at 200 nM concentration), leading us to the hypothesis of a better performance *in vivo*. We also sought to compare results obtained with and without the transfection reagent using the EC_{50} dose previously calculated for each CPP-PMO (Table 1). CPP-PMOs 6aKC23b, 6aKC-218, and 9b2-218 were able to enter the cells by gymnosis and reach their targets, as shown by significant increases observed in MBNL1 protein levels in comparison with untreated DM1 cells (Figure 1J). In fact, CPP 6aKC-linked compounds achieved a higher increase of MBNL1 levels by gymnosis than by transfection ($p = 0.0022$ and 0.0029 for antimiR-23b and antimiR-218, respectively). These differences were statistically significant, pointing toward a better performance *in vivo*. It is noteworthy that 9b2KC-23b caused identical MBNL1 modulation results by transfection and gymnosis, but toxicity was significantly reduced using the latter delivery method (Figure 1K), suggesting that 9b2KC-23b could outperform 9b2-23b in mice. The cell viability profile was generally more favorable using the gymnosis method, possibly due to the inherent toxicity of transfection reagents.

Target engagement of CPP-PMO antimiRs *in vivo*

CPP-PMOs and SC were tested *in vivo* in the HSA^{LR} mouse model using PBS-injected disease mice as procedural controls and the FVB mouse strain as a reference for Mbnl1 values. Experimental compounds were administered at a mean dose of 9.2 mg/kg intravenously every 2 weeks and mice were sacrificed 2 weeks after the third administration. Quantification of the target miRNAs, *Mbnl1* and *Mbnl2* mRNA levels, Mbnl1 protein, and alternative exon inclusion was performed in gastrocnemius and quadriceps muscles of all experimental animals using vehicle-injected HSA^{LR} mice as a reference for comparisons (Figure 2). In postmortem analysis, all six test compounds

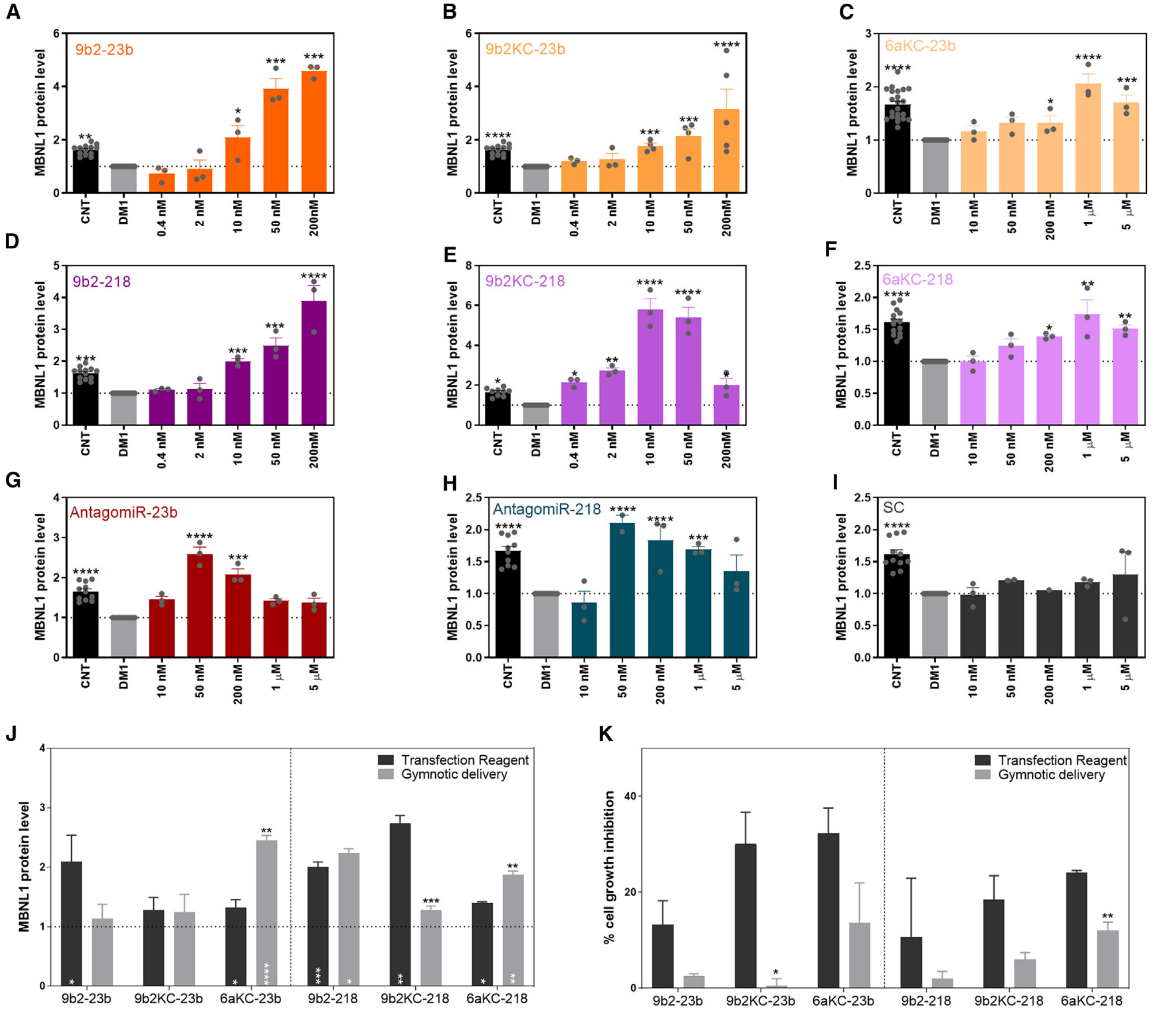

Figure 1. Activity effect of CPP-PMOs and previous antagomiRs in DM1 cells

Data show the quantification by QDB of MBNL1 protein levels after treatment with different ONs in DM1 cells at the indicated concentrations: (A) 9b2-23b, (B) 9b2KC-23b, (C) 6aKC-23b, (D) 9b2-218, (E) 9b2KC-218, (F) 6aKC-218, (G) AntagomiR-23b, (H) AntagomiR-218, and (I) scrambled control (SC). The highest value of each was used as the E_{max} of the corresponding ON. Black bars represent MBNL1 levels of unaffected control (CNT) myotubes and light gray bars represent MBNL1 levels in DM1 mock-treated cells. Fold change of (J) MBNL1 protein levels and percentage of (K) cell growth inhibition of DM1 cells using transfection reagent (black columns) or gymnosis delivery (gray columns) with ONs against miR-23b or miR-218 at their corresponding EC_{50} concentration. Dotted line indicates MBNL1 levels in DM1 cells without treatment. *$p < 0.05$, **$p < 0.01$, ***$p < 0.001$, ****$p < 0.0001$ according to one-way ANOVA compared with DM1 non-treated cells (black stars in A–I, white stars in J) or according to Student's t test between delivery strategies (black stars in J and K). Each concentration was tested in triplicate. Individual values are indicated as datapoints. Error bars indicate mean ± SEM.

significantly reduced levels of functional miR-23b and miR-218 in both muscles tested, as quantified by qRT-PCR (Figures 2A and 2B). 9b2KC-conjugated ONs showed the strongest activity against both target miRNAs in gastrocnemius (up to 90% silencing). As expected, SC control did not reduce functional levels of either of the two miRNAs, indicating the specificity of the antimiR sequences in all our experimental observations. These data are in line with our *in vitro* data.

Mbnl1 mRNA levels were determined from both muscles by qRT-PCR (Figure 2C) and Mbnl1 protein upregulation was confirmed by quantitative dot blot (QDB) (Figure 2D), thus supporting previous

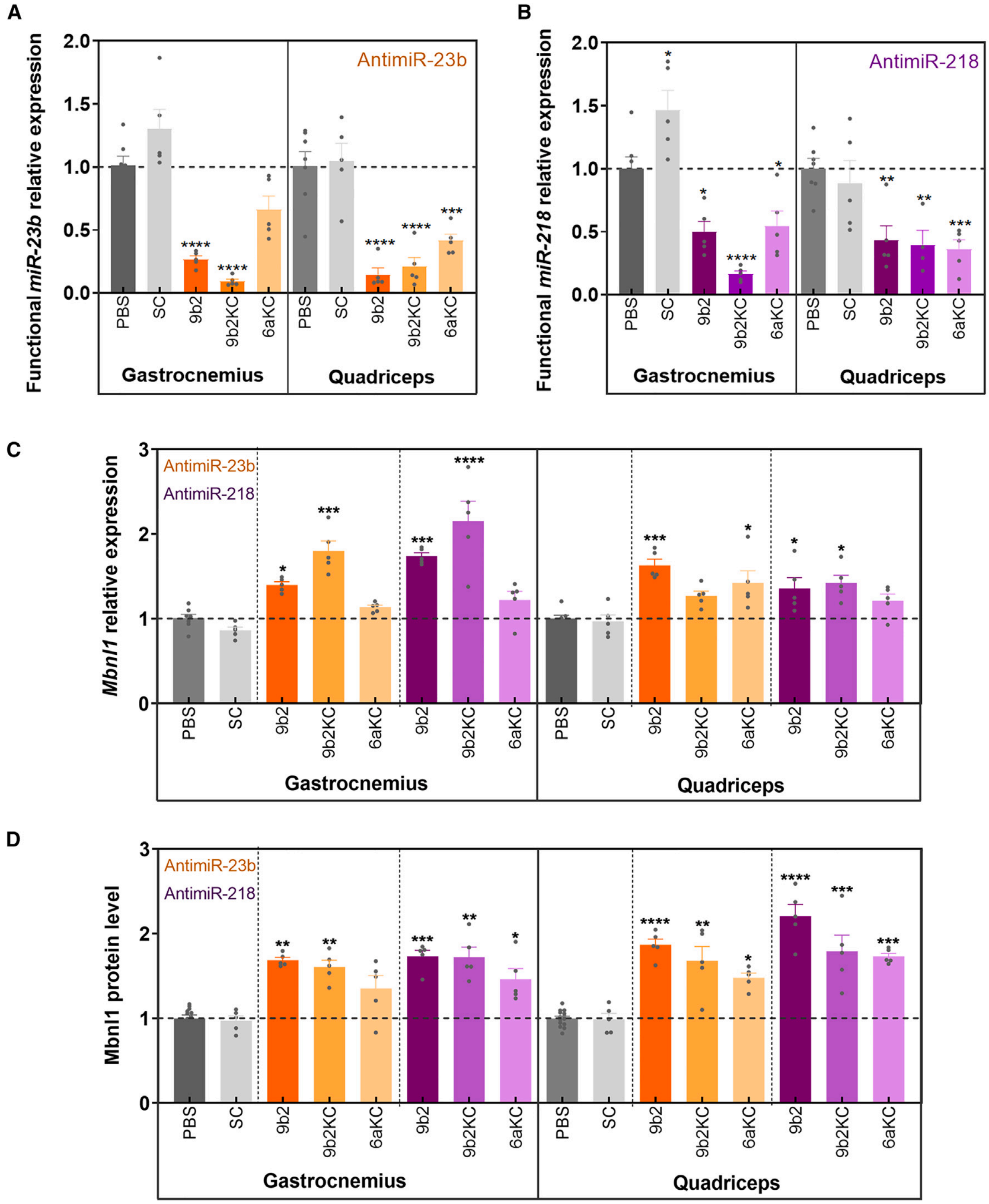

Figure 2. CPP-PMO antimiR treatment results in sustained improvement of MBNL1- and MBNL2-dependent molecular functions in *HSA*^{LR} mice
After 45 days of treatment with CPP-PMOs, gastrocnemius and quadriceps muscles were dissected to measure functional (A) miR-23b and (B) miR-218 expression relative to U1 and U6 snRNA endogenous controls, (C) *Mbnl1* transcript levels relative to *Gapdh* endogenous control on gastrocnemius and quadriceps, (D) Mbnl1 protein levels relative to endogenous tubulin control using quantitative dot blot. Statistical comparisons were performed in each case against PBS-treated *HSA*^{LR} values (indicated by a black dashed line with ANOVA one-way test. *$p < 0.05$, **$p < 0.01$, ***$p < 0.001$, ****$p < 0.0001$). PBS n = 7, SC n = 5, 9b2-218 n = 5, 9b2-23b n = 5, 9b2KC-218 n = 5, 9b2KC-23b n = 5, 6aKC-218 n = 5, and 6aKC-23b n = 5. Individual values are indicated as datapoints. Error bars indicate mean ± SEM.

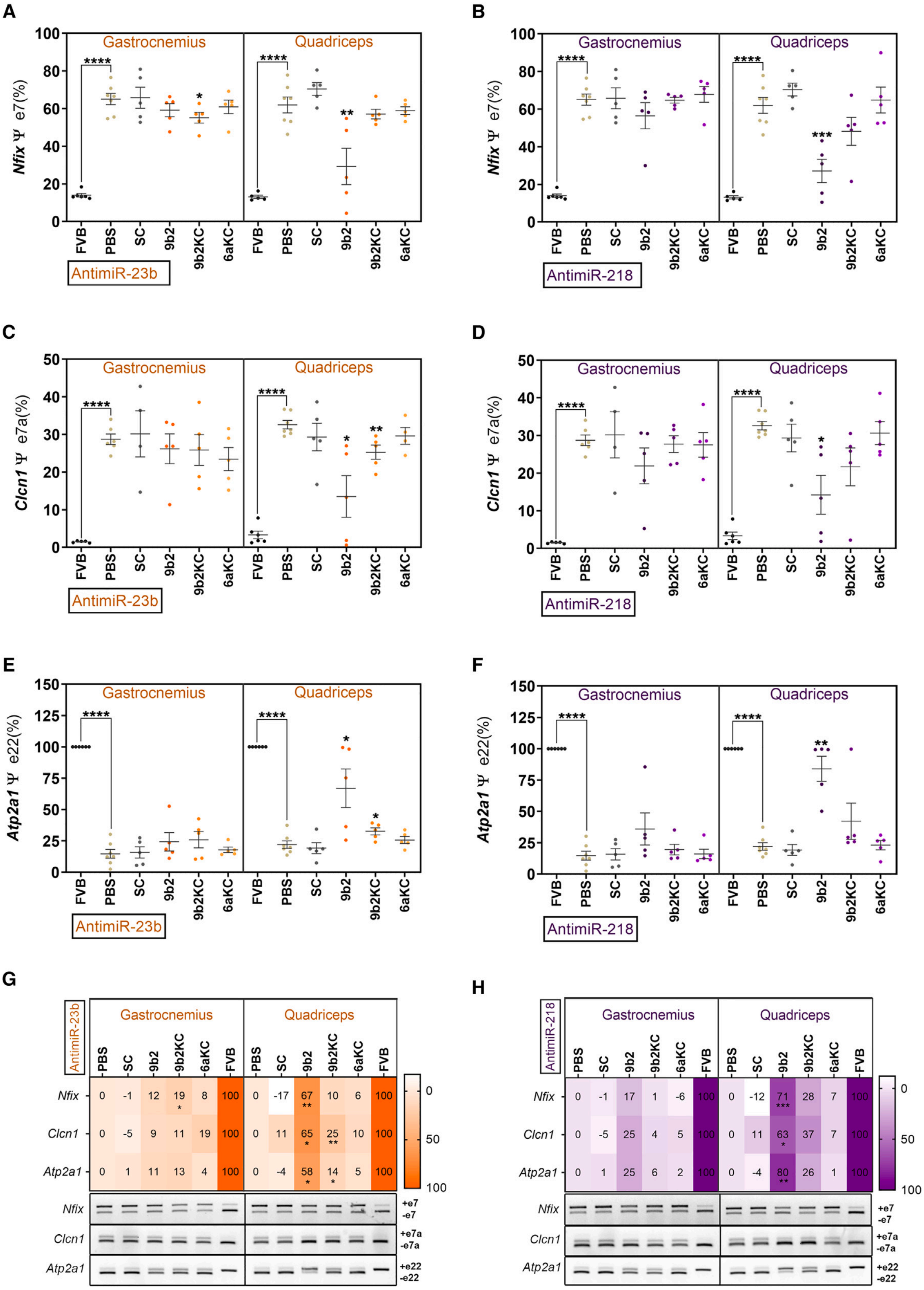

(legend on next page)

findings of reduced functional 23b and 218 miRNA levels. 9b2KC-conjugated ONs against both target miRNAs showed the strongest significant increase of *Mbnl1* levels in gastrocnemius (up to 2.15-fold for miR-218 antimiR, p = 0.0003). In the case of 6aKC-treated mice, only significant increases in quadriceps were observed for 6aKC-23b (Figure 2C). Regarding Mbnl1 protein levels, a clear and consistent trend of protein upregulation (from highest to lowest 9b2 > 9b2KC > 6aKC) was found in both muscles and against both miRNAs (Figure 2D). miR-23b and miR-218 also modulate *Mbnl2* expression, and we have previously shown that their inhibition leads to increased levels of this transcript.[20] We therefore tested by qRT-PCR whether our CPP-PMOs would also enhance mRNA levels of the *Mbnl2* gene (Figure S3A). Increased relative expression of *Mbnl2* was observed with almost every treatment in both muscles, up to 2.65-fold in the case of 9b2KC-23b in gastrocnemius (p < 0.0001). As miR-23b shares its seed region with miR-23a and presents an 87% of identity with miR-23a, candidate antimiRs against miR-23b could also potentially target miR23a. Accordingly, we performed a qRT-PCR of *miR-23a* in quadriceps of mice injected with antimiRs against miR-23, observing that all three test antimiRs significantly reduced miR-23a functional levels (Figure S3B). Interestingly, the expression levels of miR-23a and -23b (miRbase: MI0000079 and MI0000439, respectively) in muscle are similar according to different databases (human and mouse tissue atlas of small non-coding RNAs or microRNA tissue expression database),[40,41] so antimiR action in this tissue, including the effect on Mbnl1, is driven by targeting both miRNAs. This result is not an off-target effect but an additional depression of *Mbnl1* transcripts, since miR-23a is also expected to regulate Mbnl1, and our therapeutic approach is to increase Mbnl1 levels in target muscle tissues.

Focusing on hybridization-mediated effects, we have addressed possible off-targets effects of our antimiRs using bioinformatics to interrogate the reference transcriptome for complementarity to anti-miR-23b or -218 sequences using the Fuzznuc algorithm from the EMBOSS analysis suite (v.6.6)[42] to search for similarities in the human genome GRCh38.p13 using a maximum mismatch of three bases. Then, to find the biologically important similitudes, we compared the result with the human .gff file using the Bedtools intersect (v.2.28).[43] This approach identified miR-23b and the host transcript AOPEP in which this miRNA is located, with zero mismatches serving as positive controls of the approach. In addition, with one mismatch, miR-23a and the gene MAP7D2 (in the X chromosome) were identified. This gene is not expressed in cell culture[24] and has a very low expression in adult human muscle (http://www.dmseq.org/). In the case of antimiR-218, miR-218-1 and its paralog miR-218-2 and the host transcripts in which these miRNAs are located were identified (Table S1) with zero mismatches. Notably, the closest partially complementary sequences involved two mismatches, and a closer look using pre-existing RNA-seq data with antimiRs with the same sequence as the one used here[24] confirmed no significant differences in expression. Hybridizations with targets involving an even higher number of mismatches are assumed not to drive significant off-target effects, at least through this mechanism. In addition, we made use of an RNA-seq dataset generated with antagomiR-23b,[44] which has the same base-pairing capacities as the corresponding CPP-PMOs against miR-23b despite the different chemistries used. Using these data, we selected eight direct targets of miR-23b that were both significantly repressed or unchanged (Figure S4A) in a CNT vs. DM1 comparison, and were significantly upregulated comparing control and DM1 muscle cells treated with the corresponding antagomiR (CHUK, SRC, NOTCH2, TAB2, SIRT1, HMGB2, MET, and PLAU). Subsequent analyses revealed that Src was not a predicted direct target of miR-23b in mice according to miRWalk and TargetScanMouse 7 so it was removed from the analysis. Expression of the remaining genes was specifically measured by qRT-PCR in the QDB of treated mice (Figure S4B), with the supposition that their levels would be higher than in untreated controls. As expected, the majority of quantifications exhibited a trend toward upregulation that reached statistical significance in the cases indicated and was in the same range as for Mbnl1 levels (between 1- and 2-fold increase; Figure 2C). Importantly, whereas relatively small changes in mRNA amounts (assumed to translate into similar changes in protein) are likely to have minor phenotypical consequences, in a sensitized situation such as Mbnl1-depleted DM1 muscles, they are more likely to play a major role. Thus, pleiotropic effects on target expression will not likely translate into biologically meaningful responses unless these effects are somehow genetically sensitized.

CPP-PMO antimiRs improve molecular functions of Mbnl1 *in vivo*

Given the increases in both *Mbnl1* and *Mbnl2* transcripts and Mbnl1 protein levels, we next tested whether they correlated with change in the inclusion of different alternative exons regulated by these proteins (Figure 3; Table S2), such as nuclear factor 1 X-type (*Nfix*), chloride channel protein 1 (*Clcn1*), and ATPase sarcoplasmic/endoplasmic reticulum Ca^{2+} transporting 1 (*ATP2A1*). Compounds linked to 9b2 CPP achieved the best missplicing improvement in quadriceps, reaching over 60% in most cases (*Nfix* p = 0.0062 and 0.0007; *Clcn1* p = 0.0246 and 0.0218; *Atp2a1* p = 0.0421 and 0.0024, for antimiR-23b and antimiR-218, respectively in each case). In gastrocnemius, splicing was not significantly different. The 9b2KC-23b conjugate produced some splicing corrections in both muscles, but they were subtle. Overall, alternative exon inclusion roughly followed the Mbnl1 protein increase detected by QDB.

Figure 3. CPP-PMOs rescue Mbnl1 splicing alterations in mice

(A–F) Quantification of the percentage of exon inclusion (Ψ) of (A and B) Nfix exon 7, (C and D) Clcn1 exon 7a, and (E and F) Atp2a1 exon 22 in gastrocnemius and quadriceps muscles of HSA^{LR} mice upon treatment with PBS, SC, or CPP-PMOs antimiRs against (A, C, and E) miR-23b or (B, D, and F) miR-218. (G and H) Splice recovery of *Nfix ex7*, *Clcn1 ex7a*, and *Atp2a1 ex22*. Reference non-DM1 Ψ values were estimated from FVB controls. Individual values are indicated as datapoints. Error bars indicate mean ± SEM. Data were analyzed by unpaired Student's t tests compared with PBS-treated HSA^{LR} mice. *p < 0.05, **p < 0.01, ***p < 0.001, ****p < 0.0001.

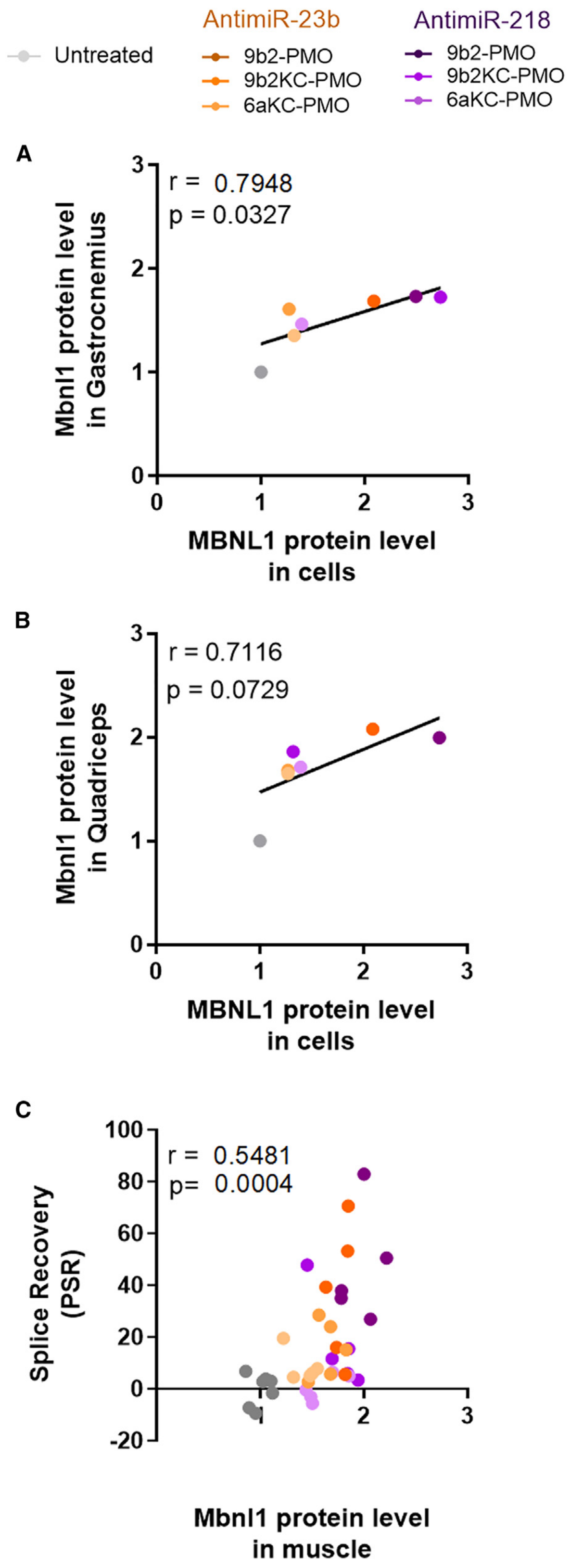

Figure 4. Activity of CPP-PMOs shows a strong correlation between *in vitro* and *in vivo* models

Positive correlation of Pearson (r > 0.5) between MBNL1 protein levels in DM1 cells and (A) gastrocnemius or (B) quadriceps tissue or (C) between average of Mbnl1 protein levels in muscle and percentage splice recovery (PSR) in individual mouse, after treatment with CPP-PMOs antimiRs. Gray dots indicate untreated samples (DM1 cells in A and B, PBS-treated HSA^{LR} mice in C).

Strong correlation between *in vitro* and *in vivo* MBNL1 protein upregulation by CPP-PMOs

Given the current controversy in the field regarding the correlation of different *in vitro* and *in vivo* evaluation methods and the importance of this question for ON drug development,[28] we next compared our cell culture experimental data from human cells with mouse model data. The correlation between these data, useful to test the predictive value of evaluating gymnosis and transfection-mediated CPP-PMO antimiRs in DM1 cells, was verified by performing a Pearson test (Figures 4A and 4B). To carry out this analysis, we used the values obtained by QDB for the cellular model at the EC_{50} concentration for each transfected compound and those obtained by this same technique for the two study muscles. Increases in the MBNL1 protein level showed a strong positive correlation between the two study models (r = 0.7116, p = 0.0729 for quadriceps; r = 0.7948, p = 0.0327 for gastrocnemius). In contrast, no significant correlation was observed between gymnosis and *in vivo* data (data not shown), suggesting that transfection may confer a higher predictive value than gymnosis in our experimental systems. In addition, we carried out a Pearson test of Mbnl1 fold change increase and percentage splice recovery (PSR), averaging the results of both muscles from treated mice (Figure 4C). The PSR ratio was calculated using exon inclusion data from *ATP2A1*, *NFIX*, and *CLCN1*. With this correlation we could see an efficacy ranking of the CPPs as 9b2 > 9b2KC > 6aKC, with a similar behavior for antimiR-23b and antimiR-218 (note the tight pairing of protein levels and PSRs brought about by antimiRs conjugated to the same CPP). The existence of a clear and significant correlation between these two parameters (r = 0.5481, p = 0.0004) suggests a functional connection between Mbnl1 protein levels and PSR, even though some of these compounds achieve only small PSR and protein upregulation.

CPP-PMO antimiRs improve histopathology signs and muscle function in HSA^{LR}

One of the most characteristic phenotypes of the HSA^{LR} mouse model is myotonia, which can be easily measured by means of electromyography. This parameter was measured every 2 weeks throughout the treatment period and a final relative change was calculated at day 45 (Figures 5C, S5A, and S5B). Mice treated with CPP-PMOs, showed a decrease in myotonia in all cases, with mice injected with the PMOs conjugated to CPP 6aKC showing the highest rescue at the endpoint (p = 0.0345 [antimiR-23b] and 0.0069 [antimiR-218]). In the case of the mice treated with SC, myotonia remained unchanged. Another DM1 feature recapitulated in HSA^{LR} mice is the presence of central nuclei in muscle fibers, indicative of regenerative attempts

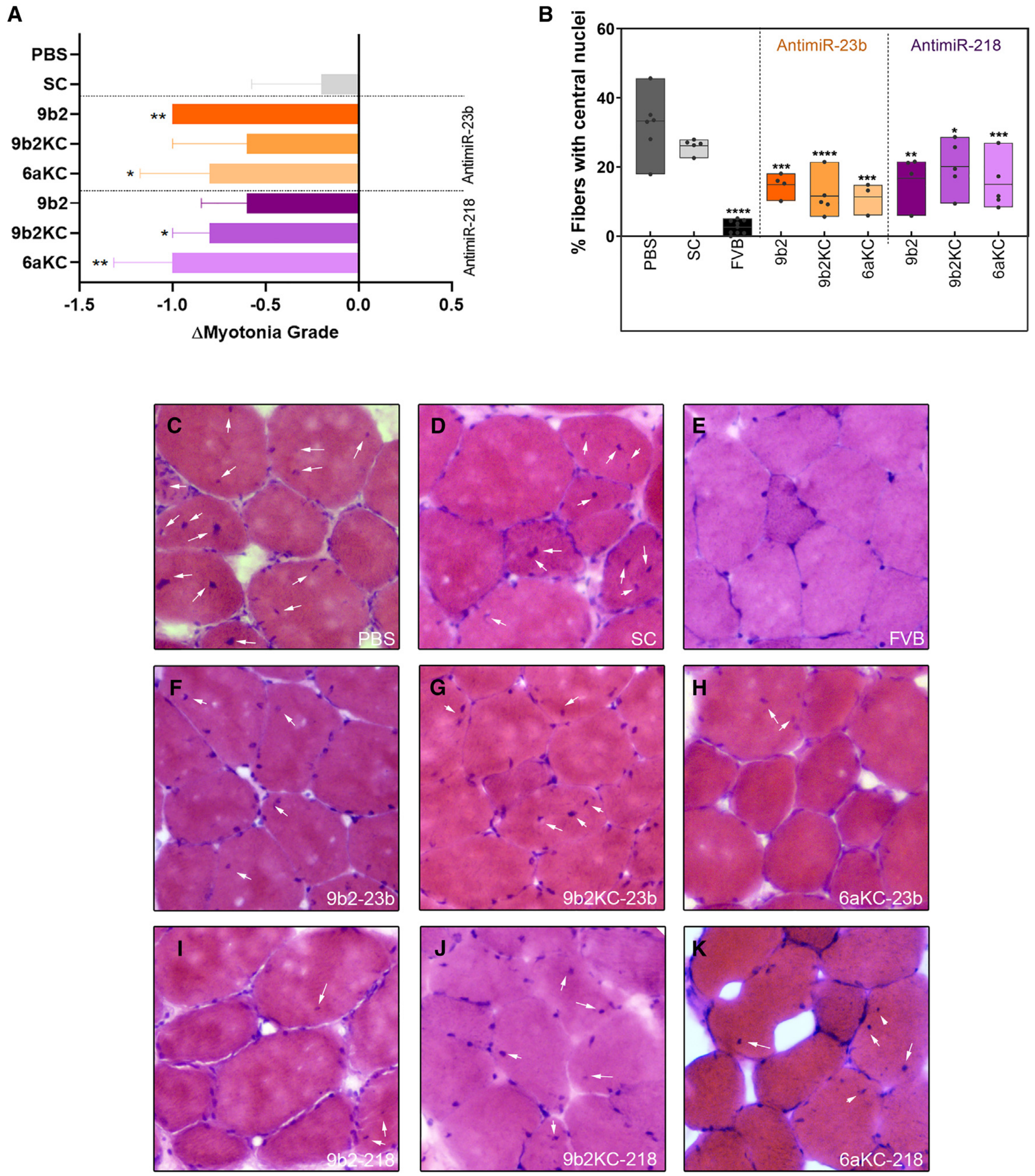

Figure 5. Recursive administration of CPP-PMO antimiRs improved functional and histological alterations in DM1 mice

(A) Relative change in myotonia grade at 45 days compared with the first injection day.

(B) Quantification of the percentage of muscle fibers with central nuclei in quadriceps of mice from each treatment group. Individual values are indicated as datapoints. (C–K) Images from hematoxylin and eosin staining of quadriceps muscles from representative mice of each treatment group. Black arrows point to examples of centrally located nuclei in muscle fibers. Error bars indicate mean ± SEM. The data were analyzed by ANOVA one-way test or Kruskal-Wallis test when it was necessary, compared with untreated HSA^{LR} mice. *$p < 0.05$, **$p < 0.01$, ***$p < 0.001$, ****$p < 0.0001$. PBS n = 7, SC n = 5, 9b2-218 n = 5, 9b2-23b n = 5, 9b2KC-218 n = 5, 9b2KC-23b n = 5, 6aKC-218 n = 5, and 6aKC-23b n = 5.

in skeletal muscle.[45] This characteristic was studied in quadriceps (Figures 5D–5M) and gastrocnemius (Figure S5) and measured as the percentage of muscle fibers with centrally located nuclei. We observed a general decrease in this parameter toward levels in control mice (FVB). The greatest improvement was observed with 6 aKC PMOs, where we obtained a 70%–80% reduction compared with mice treated with PBS. Muscles from SC-treated mice did not differ significantly from PBS-injected ones.

Toxicological assessment after treatment with CPP-PMO antimiRs

One of the biggest concerns in RNA therapeutics is liver and kidney toxicity.[46] PMO-type ONs are less toxic due to their chemical properties and are easily and rapidly excreted due to their low interaction with plasma proteins.[47] We analyzed blood samples of mice injected with CPP-PMOs and SC and of control groups treated with PBS (FVB and *HSA*^{LR}) to search for signs of toxicity (Table S3). To assess liver toxicity, we quantified serum levels of alkaline phosphatase (ALP), alanine aminotransferase (ALT), bilirubin, and aspartate aminotransferase. To check for possible renal toxicity, we analyzed the values of ALP, creatinine, urea, and lactate dehydrogenase. Taking the *HSA*^{LR} group treated with PBS as a reference, we observed a statistically significant increase in the urea values of mice treated with 9b2-23b. Significant differences were also observed in ALP, but cannot be attributed to CPP-PMO treatment since the difference was also significant in the FVB PBS group. Finally, to check for possible CPP-PMO-mediated immune system activation, the bodyweight-normalized spleen weight (Figure S6A) and weight gain or loss (Figures S6B and S6C) were analyzed in each experimental group, with no significant between-group changes found. Regarding the white blood cell differential count (Table S4), we observed several differences in monocyte and eosinophil counts non-attributable to CPP-PMO treatment due to the presence of these differences in the FVB group when compared with PBS-treated model mice.

In addition, different proinflammatory cytokines (IL-1β, IL-6, MCP-1, IL-12β, TNF-α, and IL-15) and muscle regeneration marker Mstn-1 in quadriceps muscle of treated mice were analyzed by qRT-PCR (Figure S7) for increase in any of these markers in muscle samples, which would also indicate an inflammatory response.[48–50] No treatments caused a significant increase in any of the muscles analyzed; indeed, the antimiRs that caused significant changes reduced the expression of these markers. First, the significant and specific (not detected in SC-treated mice) downregulation of Mstn-1 upon 9b2-23b and 9b2KC-23b treatment can be seen as a favorable change. This gene has a key role as a muscle mass repressor and MSTN-1 protein levels have been found downregulated in circulating blood in DM1 patients undergoing physical rehabilitation, with improved clinical functional outcome measures such as increased 6-min walking test.[51] Second, several downregulations detected seem to stem from unspecific effects of the chemistry used, particularly reduced levels of IL-6 and IL-15 levels, which are also detected in SC-treated muscles. In any case, to our best knowledge, reduced muscle levels of IL-1β, IL-6, and TNF-α have not been associated with deleterious effects on muscle homeostasis and this suggests an absence of any pro-inflammatory cytokine, as also indicated by unchanged spleen weight.

CPP-PMO antimiR levels in selected target tissues and MBNL1 regulation

Since the physiological expression levels of functional miR-23b and miR-218 vary considerably between different tissues (Figure S8), CPP-PMO penetrance is a key determinant of their silencing efficiency, especially in DM1-affected organs. As expected from the biophysical characteristics of PMO ONs and their short circulation time, CPP-PMOs mainly accumulated in kidneys (Figures 6A–6F). However, the nature of the CPP conjugated in each case also influenced the biodistribution of the cargo, since 9b2KC seemed to increase kidney concentration of the PMO. As reported in previous studies,[52] ON accumulation in liver was higher compared with our target tissues (heart, quadriceps, gastrocnemius, and brain), particularly so for 9bKC-23b. Levels of all CPP-PMOs were also reliably detected in muscle tissue. However, as expected from its high liver accumulation, lower muscle levels of 9b2KC-23b compared with the other ONs were found in muscles. In general, the ratio of ON concentration in muscle tissue compared with kidney/liver was more favorable for 6aKC-conjugated PMOs. In brain, although low levels of PMOs were detected, those conjugated to 9b2 were more efficient at crossing the blood-brain barrier (BBB) than other conjugates.

The systemic effects of the CPP-PMOs are a relevant issue, partially addressed with our kidney samples given that miR-218 does not seem to be significantly expressed in the liver (Figure S8). Using QDB determinations, we confirmed that several antimiR-23b and -218 versions could upregulate Mbnl1 in this organ (Figure 6G), despite not all versions having the same efficacy. These results indirectly confirm the suitability of both miRNAs as kidney targets to upregulate Mbnl1 levels but, as expected, also clearly point to organ-specific effects by the CPPs.

DISCUSSION

ONs represent a promising and fast-developing platform to treat neuromuscular disorders[53] and other rare diseases lacking therapeutic alternatives, resulting in 11 approved ON-based therapies, most of them in recent years.[28] Over the past decade, antisense ONs have been used to release MBNL by either targeting *DMPK* transcripts for degradation or blocking the binding between the two.[53] In contrast to most genetic diseases whose phenotypes originate from loss- or gain-of-function mutations in a particular gene, most molecular DM1 alterations stem from the functional depletion of MBNL proteins alone,[54,55] which remain encoded in perfectly functional genes. From a therapeutic perspective, this is a particularly favorable situation because we can compensate for protein depletion with additional endogenous expression, which may also correct the excessive activity of MBNL antagonists. An alternative approach to neutralizing the toxic effect of expanded DMPK transcripts therefore is to restore normal free levels of MBNL1 and/or MBNL2 proteins, since these genes are advantageous for use as therapeutic targets. In fact, it has been shown that MBNL1/2 overexpression is well tolerated *in vivo* and *in vitro* and ameliorates some typical DM1 alterations.[17,20,23,24,56]

Despite the therapeutic potential of ONs as RNA modulators to treat diseases, further development is hindered by delivery problems in most clinical applications. As a multisystemic disease, delivery is a particularly challenging endeavor in DM1 because multiple tissues and organs need to be targeted, including a large portion of the

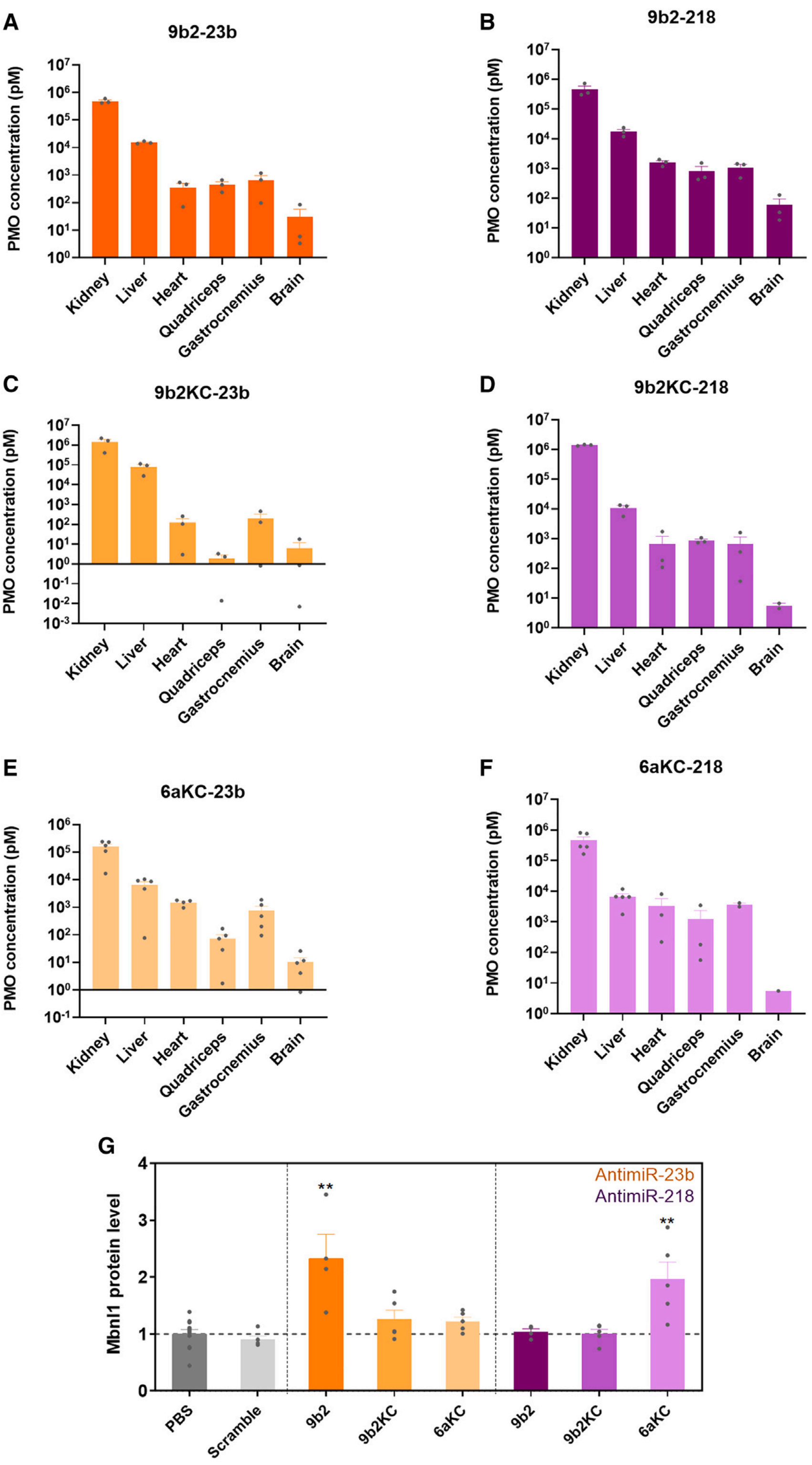

Figure 6. Levels of CPP-PMO antimiRs and Mbnl1 in selected target tissues

After 45 days of treatment with the CPP-PMOs, different tissues were dissected to measure by ELISA levels of each antimiR: (A) 9b2-23b, (B) 9b2-218, (C) 9b2KC-23b, (D) 9b2KC-218, (E) 6aKC-23b, and (F) 6aKC-218. The selected target tissues were kidney, liver, heart, quadriceps, gastrocnemius, and brain. (G) Mbnl1 protein levels in kidney relative to endogenous tubulin control using quantitative dot blot. Individual values are indicated as datapoints. Error bars indicate mean ± SEM. The data were analyzed by Kruskal-Wallis test, compared with untreated HSA^{LR} mice $^{**}p < 0.01$.

skeletal muscles of the body (a tissue especially hard to penetrate). In fact, the ON that reached the furthest therapeutic development stage for DM1[57] failed during clinical evaluation (ClinicalTrials.gov ID: NCT02312011), reportedly due to insufficient bioavailability in muscle tissue. Since then, research efforts in new ON studies have increasingly focused on strategies to enhance cellular uptake and delivery to DM1-affected cells.[34] Systemic administration of ONs frequently results in poor effective tissue uptake because delivery occurs predominantly via the endocytic system, from which these molecules must escape to avoid degradation by the lysosomal environment.[58] Endosomal entrapment is a common therapeutic bottleneck, and only a very small percentage of ON molecules escape the endosomes to become available at the site of action.[59,60] To address this problem, chemical modifications and delivery agents have been developed with the goal of increasing the rate of cellular uptake, intracellular trafficking, and endosomal escape.[27,61] CPPs can trigger the uptake of different types of large macromolecules across cell membranes.[62–64] Once internalized, CPPs can also help to induce endosomal escape.[65] For systemic diseases such as DM1, CPPs pose a promising approach to delivering ONs across multiple tissues. They have been developed for uptake into particularly difficult-to-reach tissues, such as skeletal muscle, heart, and CNS (all affected in DM1).[66,67] A recent study has shown that CPP-PMOs can cross the BBB efficiently in a mouse model of spinal muscular atrophy.[68]

Our results constitute a first proof-of-concept of antimiRs conjugated to CPPs to improve biodistribution and activity *in vivo*. Our collection of CPP-PMOs combine an efficient delivery method with ONs to enhance MBNL levels, in what could represent a paradigm shift for DM1 treatment. Cholesterol conjugation is another widely used ON delivery strategy[28] that has been applied to silence miR-218 and miR-23b with antagomiRs in previous experiments.[20,23,24] However, to the best of our knowledge, this is the first study performing side-to-side comparison of CPP with cholesterol conjugation for miRNA targeting. We identified several CPP-PMOs with higher efficiency and lower toxicity *in vitro* compared with our previous antagomiRs for miR-218 and miR-23b.[20,23,24] This was demonstrated by estimating the T_{index} of these ONs, which represents the dose window that can be used to reach the desired effect (restoration of MBNL levels to a normal range) without having toxic effects. According to the maximum efficiency (E_{max}), CPP-PMOs can be used to increase MBNL to levels well above those observed in unaffected control cells. This allows for ON doses to be adjusted to correct DM1 alterations without causing signs of cellular toxicity. For example, 9b2KC produced a restoration of MBNL1 to the same levels of unaffected cells using a dose several orders of magnitude below the TC_{50}. In this regard, it can be concluded that conjugation of PMO ONs against miR-23b or miR-218 to 9b2KC improved T_{index} compared with conjugates with the previous 9b2 CPP version. 6aKC PMO conjugates, in contrast, worsened or improved over antagomiRs depending on the specific target miRNA. Thus, *in vitro*, CPP-PMOs have a therapeutic potential that outperforms reported results for antagomiRs in DM1.

The data obtained in the DM1 mouse model confirmed the activity and favorable safety profile of the CPP-PMOs *in vivo*. 9b2 and 9b2KC conjugates reduced functional levels of their corresponding miRNA targets and increased MBNL1 expression in gastrocnemius and quadriceps muscles. 6aKC PMOs were less effective but also significantly increased MBNL1 levels in both muscles. Increased MBNL1 levels *in vivo* improved some of the most characteristic splicing alterations in DM1 relevant for muscle function. This resulted in an alleviation of myotonia grade in both muscles tested. Interestingly, a recent paper[69] reported a similar reduction in myotonia of 1° upon 12.5 mg/kg (similar dose as ours) 10 days after injection of an ON targeting the CUG repeats, while our therapeutic approach aims to block miRNAs that repress MBNL1 expression. miRNAs with shared seed regions, such as miR-23b and -23a, are certainly at risk of being blocked by the same antimiR, in this case antimiR-23b. This is expected from the antimiR approach since it is well-known that miRNAs repress the expression of hundreds of genes mainly based on complementarity to the seed region, generally weakly and in concerted action with several others.[70] However, contrary to acting on a normally expressed target miRNA, we have previously reported that miR-218 is actually upregulated in DM1 cell and mouse models and patient muscle biopsies.[24] Thus, when we act on miR-218, we intend to derepress the expression of all miR-218 targets, which critically includes MBNL1 and MBNL2 transcripts but may include several others. We are currently addressing similar quantifications with miR-23b levels. In addition, we have seen no expressed RNA in the human transcriptome (except for the intended target miRNA) is perfectly complementary or shows a single mismatch against the antimiR-218 sequence, and the closest antimiR-23b *off-target* RNA has one mismatch (MAP7D2), but it is not expressed in cell culture[24] and has a very low expression in muscle (http://www.dmseq.org/).

The number of central nuclei, another characteristic feature of DM1 muscles, was also remarkably reversed, indicating that CPP-PMOs could correct dystrophic alterations during the 45 days of the experiment. Despite the relatively modest effects of some CPP-PMOs on RNA splicing, activities of 9b2-23b and −218 stand out from other versions in terms of splicing recovery and Mbnl1 upregulation. In our view, several functions beyond alternative splicing control almost certainly contribute to the improvement in muscle function *in vivo*, since Mbnl proteins have been involved in gene expression at several stages including predominantly alternative splicing, but also alternative polyadenylation, microRNA biogenesis, mRNA stability, transcription, translation efficiency, and mRNA transport.[71–78] Therefore, since antimiRs bring about increased levels of Mbnl1 proteins, improvements in any of these Mbnl1-dependent functions will likely contribute to functional rescues *in vivo*. PMOs conjugated to 9b2 and 9b2KC performed better than 6aKC in this respect, as could be expected from their corresponding effect on MBNL1 levels. However, 6aKC-PMOs had a slightly superior effect on myotonia, perhaps because of their superior action correcting its cause: the abnormal splicing on *Clcn1* transcripts.[79] Altogether, these data show the therapeutic potential of CPP-PMO antimiRs for DM1.

Most CPPs currently used to deliver cargoes are R-rich peptides, such as 9b2. As an example, Sarepta therapeutics is testing the R6gly peptide conjugated to a splice switching PMO (SRP-5051) for the treatment of Duchenne muscular dystrophy patients in a phase II clinical trial (ClinicalTrials.gov ID: NCT04004065). Increasing the arginine content of the CPP has a favorable effect on both endocytic uptake and endosomal escape, but it is also associated with toxicity.[80] An approach to reducing R-content while keeping some of the properties of the compound involves the replacement of R with other basic amino acids such as K, as we did in 9b2KC. K is less basic than R and interacts with cell-surface proteoglycans differently.[81] However, it is generally thought that replacing R with K reduces endosomal escape resulting in poor activity.[81,82] Here, we show that K-rich peptides can have potential as delivery agents with wide therapeutic windows when addressed at miRNA targets. Our results are in accordance with previous *in vitro* observations, in which endosome trapping did not significantly affect the antimiR activity of a K-conjugated ON targeting miR-122.[83] The subcellular localization of the antimiR suggested that targeting of miR-122 may take place in or associated with endosomal compartments, where ON is delivered by the cationic peptide. This opens the possibility that miRNA targets have different dynamics in terms of subcellular trafficking and localization when compared with nuclear targets such as those of splice-switching ONs. These different requirements could allow us to fine-tune CPPs to miRNA targets, reducing toxic interactions of the PMO in the nucleus and increasing the therapeutic window of compounds. It would be interesting to test this hypothesis in future studies due to its potential implications for optimizing therapeutic strategies.

From our *in vitro* toxicity evaluation, we can conclude that the R to K substitutions in 9b2KC indeed increased the TC_{50}, conferring a safer profile to this peptide compared with the 9b2 version. The sequence of the PMO to toxicity also contribute specifically, as we obtained higher TC_{50} in 9b2-23b and 9b2KC-23b than 9b2-218 or 9b2KC-218. The TC_{50} of both PMOs conjugated to 6aKC (also containing R to K substitutions) achieved the highest levels of our panel, above the other CPP-PMOs and previously tested antagomiRs. This allowed us to test 6aKC PMOs at a higher concentration by gymnosis, achieving significantly better results than transfection. In *in vivo* evaluation, no alterations in biomarkers for renal or liver toxicity could be specifically attributed to any PMO or CPP, nor in the different proinflammatory cytokines and muscle regeneration marker Mstn-1 analyzed in quadriceps muscle of treated mice.

ON biodistribution with advanced delivery strategies can potentially limit toxicity and off-target effects, as these molecules can be designed to target specific tissues where the activity is required, while leaving other less exposed to the drug. Targeted delivery of ONs to specific tissues like the liver, CNS, skeletal muscle, spleen, pancreas, or eye is an active field of research.[84,85] This was particularly relevant in antimiRs, allowing us to prevent unwanted reduction of the miRNA targets in tissues not affected by the disease by modulating PK/PD characteristics. In this regard, the ratio of accumulation in muscle tissues compared with liver/kidney was superior in 6aKC PMOs compared with those conjugated to 9b2 and 9bKC. We hypothesized that these differences between PK profiles could account for the distinct PD patterns observed. As mentioned previously, the most effective PMOs (i.e., with lower EC_{50} values, those conjugated to 9b2 and 9b2KC) outperformed 6aKC PMOs, correcting molecular alterations like *Mbnl1* mRNA/protein levels and characteristic missplicing events in DM1. However, the more favorable biodistribution of 6aKC PMOs may have allowed them to reside in our target tissues/cells for longer periods of time. Distribution data must be interpreted with caution; for example, while they can quantify the candidate antimiR in the corresponding organ homogenate, they cannot pinpoint whether the oligo became stuck in vein walls, connective tissue, or other areas, or actually entered the cell types of interest. This could explain why 9b2KC versions of the PMOs are readily detected by ELISA in kidney samples but were unable to upregulate Mbnl1 compared with other antimiR versions that did show such capacity and easily reached the kidney (6aKC). In fact, 6aKC PMOs worked best for myotonia reduction and those DM1 alterations requiring long recovery periods after Mbnl1 levels are restored, such as central nuclei. Myotonia alleviation depends on the expression of the correct Clcn1 channel isoform, followed by the accumulation of CLCN1 proteins in the membrane of muscle fibers. The process of correcting central nuclei may be even slower, since multiple parameters of muscle tissue homeostasis must be adjusted to halt the regenerative program. Concerning the correlation of *in vitro* and *in vivo* data, we speculate that the effect of 9b2 and 9b2KC conjugates observed by transfection anticipated a fast peak of intense activity *in vivo*, while gymnosis may have been superior to predict longer residing times of 6aKC PMOs in muscle tissue.

This study opens new perspectives for the treatment of myotonic dystrophy, tackling fundamental delivery and toxicity aspects currently standing in the way of developing effective therapies. More research is warranted to fine-tune other pharmacological aspects of DM1 CPP-PMO antimiRs before advancing these drugs to clinical trials, but the present study paves the way for further development of these molecules as viable and powerful candidates prior to their possible application to DM1 patients. The good momentum shown by other ON drugs, which are recently being moved forward for approval in several other neuromuscular disorders, reflects an encouraging era in which new therapeutic opportunities for DM1 could potentially open in the coming years.

MATERIALS AND METHODS

Conjugated ONs

The following CPPs containing two flanking regions enriched with cationic amino acids (arginines or lysines) and a central hydrophobic core were used in this study: 9b2, AcRXRRBRRFQILYRBRXRB; 9b2KC, -AcKXKKBKKFQILYKBKXKC- (NH2)Ma.HEX; 6aKC, -AcKXKKBKKXKYQFLIKXKBKXKB (NH2)-Ma.HEX. Amino acids were L stereoisomers, except for the non-natural B and X that have no side chains. CPPs were synthesized and conjugated to phosphorodiamidate morpholino oligonucleotides (PMOs) through a maleimide

linkage at the 3′ end of the PMO, followed by purification by cationic ion exchange chromatography and analyzed by MALDI-TOF MS as described previously.[86] PMOs are non-ionic ONs synthesized after replacing the phosphodiester bond by phosphoramidate linkage and the ribose with a morpholino moiety. PMOs were purchased from Gene Tools LLC (Philomath, OR) and their sequences are shown in Table 1. Each CPP was conjugated with a PMO against miR23b and to another PMO against miR-218, for a total of six CPP-PMO molecules. We used a scrambled PMO sequence conjugated to 9b2 CPP (SC) as a negative control.

Cell culture experimentation

Immortalized MyoD-inducible (doxycycline) DM1 and control fibroblasts were kindly provided by Dr Furling (Institute of Myology, Paris, France).[38] For the activity assay, cells were seeded in 6-well plates at a density of 8×10^4 cells/well and grown in DMEM with 4.5 g/L glucose, 1% P/S, and 10% FBS (Sigma, St. Louis, MO). To transdifferentiate fibroblasts into myoblasts by inducing MyoD expression, the cells were plated in muscle differentiation medium containing DMEM with 4.5 g L^{-1} glucose, 1% P/S, 2% horse serum, 1% apo-transferrin (10 mg mL^{-1}), 0.1% insulin (10 mg mL^{-1}), and 0.02% doxycycline (10 mg mL^{-1}). In all cases, the cells were grown at 37°C in a humidified atmosphere containing 5% CO_2. Transdifferentiation was induced at day 0, and transfection was performed by lipofection with X-tremeGENE HP (Roche, Basel, Switzerland) 24 h later, with antimiRs and test compounds in the cell medium at different concentrations (from 0.4 nM to 5 μM). The medium was replaced with fresh differentiation medium 4 h afterward. In the gymnosis assay, test compounds were added to the culture medium only at the corresponding EC_{50} concentration. Cells were collected on day 4 of differentiation and were processed for protein extraction.

Cell proliferation assay

Cells seeded at 10^5 cells/mL in 96-well plates were transfected 24 h later with antimiRs as described previously.[20] After 96 h, cell proliferation was measured using the CellTiter 96 AQueous Non-Radioactive Cell Proliferation Assay (Promega, Madison, WI). Absorbance levels were determined using an Infinite M200 PRO plate reader (Tecan, Männedorf, Switzerland) and the TC_{50} was calculated using non-linear least-squares regression.

Protein extraction, QDB, and WB assay

For total protein extraction, human muscle cells were sonicated while mouse muscles (gastrocnemius and quadriceps) were homogenized in Pierce RIPA buffer (Thermo Scientific, Waltham, MA) supplemented with protease and phosphatase inhibitor cocktails (Roche Applied Science, Penzberg, Germany). Quantification of total protein was performed with a Pierce BCA protein assay kit (Thermo Scientific) using bovine serum albumin as standard.

A 96-well plate QDB assay was used[87] to determine MBNL1 protein levels in all replicates and concentrations of each ON tested. This technique allows for high-throughput screening of low protein concentration samples over conventional western blot (WB) (Figure S9).[88] Cell samples (1 μg/well) and mice samples (2 μg/well) were prepared with 4× loading buffer, denatured (100°C for 5 min) and loaded in QDB plates (Quanticision Diagnostics, Research Triangle Park, NC). For the quantification of *in vitro* samples, the QDB was performed exactly as described in Moreno et al.[87] Each cell sample was loaded in quadruplicate in two different plates; one was used to detect MBNL1 (it was incubated overnight with primary mouse anti-MBNL1 [1:1,000, Abcam, ab77017]) and the other for GAPDH (it was incubated overnight with primary mouse anti-GAPDH [1:500, Santa Cruz, clone G-9]), which was used here as an endogenous control. For Mbnl1 quantification in mouse muscle tissues, we implemented the following modifications: each sample was loaded in quadruplicate on three different plates. One was used to detect Mbnl1 with a mouse MB1a(4A8) antibody (DSHB, Iowa City, IA) using a 1:200 dilution. A second plate was used to quantify tubulin (endogenous normalization control) with a mouse 12G10 antibody (DSHB) using a 1:1,000 dilution. A third plate was used to quantify IgG signal (negative control to subtract background) using an anti-mouse-IgG secondary antibody (Sigma-Aldrich) at 1:3,500 dilution. This subtraction was necessary due to an unspecific IgG signal could be observed in mouse samples (Figures S10A–S10D). All primary antibodies were detected using horseradish peroxidase-conjugated anti-mouse-IgG secondary antibody (1:5,000 for the anti-mouse plate, 1:2,000 for cell plates, and 1:3,500 for mouse plates [Sigma-Aldrich, MO]). Immunoreactive bands were detected using Pierce ECL Western reagent (Thermo Scientific), and luminescence levels were determined using an Infinite M200 PRO plate reader (Tecan). The validity of this correction for QDB quantification of mouse samples was confirmed by performing a series of experiments determining both the window of linearity of the assay (2–5 μg protein, Figures S10A–S10B) and the comparison with data obtained by WB in a subset of samples (Figures S10E–S10H), obtaining a correlation of 0.9927 for MBNL1 and 0.8678 for tubulin (in the case of quadriceps), and 0.9821 for MBNL1 and 0.9699 for tubulin (in gastrocnemius). For WB assay in quadriceps (Figure S10I), samples were loaded in pools of each treatment to have a representative WB of all the treatments tested. WB assay was performed as described previously.[20] These experiments confirmed the validity of QDB for our study and demonstrated that this technique was more suitable than traditional WB for our goal of analyzing a large collection of samples of limited concentration.

RNA extraction and qRT-PCR

Total RNA from murine gastrocnemius and quadriceps muscle was isolated using the miRNeasy Mini Kit (QIAGEN, Hilden, Germany) according to the manufacturer's instructions. One microgram of RNA was digested with DNase I (Invitrogen, Carlsbad, CA) and reverse transcribed with SuperScript II (Invitrogen) using random hexanucleotides. For subsequent PCR reactions, 20 ng of cDNA was used with GoTaq polymerase (Promega). Specific primers were used to analyze the alternative splicing of *Nfix*, *Atp2a1*, and *Clcn1* in mouse samples (both muscles). *Gapdh* levels established the endogenous reference levels using 0.2 ng of cDNA. PCR products were separated on a 2% agarose gel and quantified using ImageJ software (NIH,

Bethesda, MD). PSR index was defined as value$\%_{SI}$ minus $\overline{X}\%_{DSI}$, divided by $\overline{X}\%_{DSI}$ minus $\overline{X}\%_{HSI}$ (SI, splicing inclusion of each sample; DSI, disease splicing inclusion; HSI, healthy splicing inclusion). Overall, PSR indicates the amount in both muscles analyzed. Primer sequences and exons analyzed are available in Cerro-Herreros et al.[20] In the case of off-targets, interleukins, and muscle regeneration marker Mstn-1, primers sequences are available in Table S5. We used 1 ng of mouse tissue cDNA as a template for multiplex qPCR using the QuantiFast Probe PCR Kit reagent. Commercial TaqMan probes (QIAGEN) were used for mouse (*Mbnl1* and *Mbnl2*; FAM-labeled probes) and reference (*GAPDH*; MAX-labeled probe) genes. Results were normalized to *Gapdh* endogenous gene expression.

miRNA expression in muscle tissues was quantified using specific miRCURY-locked nucleic acid microRNA PCR primers (QIAGEN) according to the manufacturer's instructions. Relative gene expression was normalized to U1 and U6 snRNAs.

Expression levels were measured using a QuantStudio 5 Real-Time PCR System (Applied Biosystems, Foster City, CA). Expression relative to the endogenous gene and control group was calculated using the $2^{-\Delta\Delta Ct}$ method.

ELISA determinations

Custom ELISA-based measurements of antimiR conjugate concentrations in brain, kidney, liver, heart, gastrocnemius, and quadriceps from treated HSA^{LR} mice were performed as described in Burki et al.,[89] using the following phosphorothioate probes: for miR-218 (5′ → 3′) [DIG] T*T*G*T*G*C*T*TGATCTA*A*C*C*A*T*G*T [BIO]; for miR-23b (5′ → 3′) [BIO] A*T*C*A*C*A*T*TGCCAGG*G*A*T* T*A*C*C [DiG] double-labeled with digoxigenin (DIG) and biotin (BIO). In all cases, a control sample was used to subtract the background (HSA^{LR} treated with PBS for 9b2 and 9b2-KC ONs; HSA^{LR} treated with Scramble for 6aKC ONs). Raw data are shown in Table S7.

Animal experimentation and CPP-PMO administration

Mouse handling and experimental procedures followed the European law regarding laboratory animal care and experimentation (2003/65/ C.E.) and were approved by Conselleria de Agricultura, Generalitat Valenciana (reference no. A1539961490857). We used homozygous transgenic HSA^{LR} (line 20 b) mice,[90] which were kindly provided by Dr Thornton (Rochester, NY) and were housed and bred in our animal facility. Experimental groups were FVB as healthy control and HSA^{LR} treated with a scrambled ON (SC) and PBS as negative controls, and HSA^{LR} mice treated with all six experimental CPP-PMOs. The mice were randomly allocated to the different treatments as follows: in each cage, each mouse was ear-marked as it was taken out, and each mouse was then assigned to a different treatment. Animals were injected biweekly intravenously with 150 μL of 1× PBS vehicle (controls; HSA^{LR} n = 7 and FVB n = 6) or 150 μL of a solution 1.875 mg/mL of the test CPP-PMOs corresponding to an average dose of 9.2 mg/kg (HSA^{LR} mice: SC n = 5, 9b2-218 n = 5, 9b2-23b n = 5, 9b2KC-218 n = 5, 9b2KC-218 n = 5, 6aKC-218 n = 5, and 6aKC-23b n = 5; see Table S6 for precise dosing). Mice were sacrificed 45 days after the first injection and the tissues of interest were frozen in liquid nitrogen for the molecular and histological assays. All the mice used in this study ranged from 5 to 5.5 months of age and all were males.

Electromyography studies

Electromyography was performed before the treatment, at the halfway point and time of sacrifice under general anesthesia, as described previously.[18] The determination was performed blindly to eliminate bias. Five needle insertions were performed in each quadriceps muscle of both hind limbs, and myotonic discharges were graded on a 5-point scale: 0, no myotonia; 1, occasional myotonic discharge in ≤50% of the needle insertions; 2, myotonic discharge in >50% of the insertions; 3, myotonic discharge in nearly all of the insertions; and 4, myotonic discharge in all insertions.

Blood assays

At 45 days post treatment, animals were sacrificed, and blood was collected by cardiac puncture exsanguination with K3-EDTA (Sarstedt, Nümbrecht, Germany). The samples were analyzed by Laboratorios Montoro Botella (Valencia, Spain). White blood cell differential counts (monocytes, stab cells, segmented cells, basophils, eosinophils, and lymphocytes) were measured with the Haematology Cell Counter ADVIA 120 (Siemens, Berlin, Germany). The serum biochemistry profile (creatinine, urea, amylase, ALP, ALT, bilirubin, lipase, and bile acids) was analyzed using a cobas 600 CCE modular analyzer (Roche).

Histology methods

Frozen 15-μm sections of mouse gastrocnemius and quadriceps muscles were stained with hematoxylin and eosin and mounted with DPX Mountant for histology (Sigma) according to standard procedures. Images were taken at a 100× magnification with a DM4000 microscope (Leica, Wetzlar, Germany). The percentage of fibers containing central nuclei was quantified in a total of 200 fibers in each mouse.

Statistical analyses

After obtaining the TC_{50}, EC_{50}, and E_{max} *in vitro*, we defined a T_{index} as follows:

$$T_{index} = \frac{TC_{50} \times E_{max}}{EC_{50}}$$

The higher this parameter, the more variable the concentrations reached, median toxicity, and activity of the compound. The E_{max} modulates the parameter, rewarding very active compounds, although median toxicity and activity concentrations might be similar, and penalizing compounds with low overall activity, despite being very different from median toxicity concentration.

When comparing the mean values of molecular and functional quantitative parameters, we performed one-way ANOVA analysis when possible. We used the Shapiro-Wilk normality test prior to hypothesis testing. Normal samples were Brown-Forsythe corrected when necessary. Samples not following normal distribution were compared by

Kruskal-Wallis without Dunn correction. The statistical significance threshold was set at $p < 0.05$. The remaining statistical differences were estimated by two-tailed Student's t test ($\alpha = 0.05$), applying Welch's correction when necessary. The sample size (n) is provided in each figure. Table S2 contains the raw data of alternative exon inclusion determinations from the indicated muscles and Table S6 contains the raw data of each mouse for age, qRT-PCR, QDB, WB, spleen relative weight, myotonia grade, weight, and dose. Statistical analyses of the average values of complete blood counts and serum biochemistry profiles were performed as described in Cerro-Herreros et al.[23]

DATA AND CODE AVAILABILITY

All data generated or analyzed during this study are included in this published article.

SUPPLEMENTAL INFORMATION

Supplemental information can be found online at https://doi.org/10.1016/j.omtn.2023.09.001.

ACKNOWLEDGMENTS

This work was made possible by the "*Therapeutic targeting of MBNL microRNAs as innovative treatments for myotonic dystrophy*" research grant (HR17-00268; TATAMI project), from the Fundación la Caixa Bank, the PROMETEO/2020/081 project (Generalitat Valenciana) granted to R.A. and the Medical Research Council (MRW014742/1) to M.J.W. Part of the equipment used in this work was funded by Generalitat Valenciana and co-financed with ERDF funds (OP ERDF of Comunitat Valenciana 2014–2020). I.G.-M. was supported by the Generalitat FDEGENT/2020/001 predoctoral fellowship Valenciana. Antibody MB1a (4A8) was provided by MDA Monoclonal Antibody Resources. The authors thank Miguel A. Beltrán from the Incliva Bioinformatics and Biostatistics Unit for contributing to the statistical analyses of mouse blood and toxicity biomarker data, Inmaculada Noguera for veterinary assistance in the University of Valencia SCSIE animal facility, Raquel Pérez-Gómez for her support with statistical analysis, and Mathieu Fischer from Oxford University for his technician contribution to this work. We are also grateful to Medical Science Consulting (Valencia, Spain) for scientific writing support, which was funded by the TATAMI grant.

AUTHOR CONTRIBUTIONS

Conceptualization, A.A., M.A.V., B.L., and R.A.; investigation, I.G.-M., E.C.-H., N.M., M.C.-S., D.P.-L., D.S.-M., A.A., Y.J., R.R., and M.A.V.; formal analysis, I.G.-M., E.C.-H., D.S.-M., Y.J., R.R., and M.A.V.; visualization, I.G.-M., and E.C.-H.; writing – original draft, I.G.-M. and R.A.; methodology, I.G.-M., E.C.-H., and A.G.-R.; data curation, J.E.-E.; funding acquisition, M.J.W. and R.A.; supervision, M.J.W., B.L., and R.A.; project administration, M.A.V. and B.L.; resources, R.A.

DECLARATION OF INTERESTS

B.L. and R.A. are founders, and CEO and scientific consultant of Arthex Biotech, respectively. Arthex Biotech is developing antimiRs for use in the treatment of DM1.

REFERENCES

1. Brook, J.D., McCurrach, M.E., Harley, H.G., Buckler, A.J., Church, D., Aburatani, H., Hunter, K., Stanton, V.P., Thirion, J.P., and Hudson, T. (1992). Molecular basis of myotonic dystrophy: expansion of a trinucleotide (CTG) repeat at the 3' end of a transcript encoding a protein kinase family member. Cell *68*, 799–808.
2. Ozimski, L.L., Sabater-Arcis, M., Bargiela, A., and Artero, R. (2021). The hallmarks of myotonic dystrophy type 1 muscle dysfunction. Biol. Rev. Camb. Phil. Soc. *96*, 716–730.
3. Ashizawa, T., Gagnon, C., Groh, W.J., Gutmann, L., Johnson, N.E., Meola, G., Moxley, R., 3rd, Pandya, S., Rogers, M.T., Simpson, E., et al. (2018). Consensus-based care recommendations for adults with myotonic dystrophy type 1. Neurol. Clin. Pract. *8*, 507–520.
4. Goers, E.S., Purcell, J., Voelker, R.B., Gates, D.P., and Berglund, J.A. (2010). MBNL1 binds GC motifs embedded in pyrimidines to regulate alternative splicing. Nucleic Acids Res. *38*, 2467–2484.
5. Batra, R., Charizanis, K., Manchanda, M., Mohan, A., Li, M., Finn, D.J., Goodwin, M., Zhang, C., Sobczak, K., Thornton, C.A., and Swanson, M.S. (2014). Loss of MBNL leads to disruption of developmentally regulated alternative polyadenylation in RNA-mediated disease. Mol. Cell *56*, 311–322.
6. Lin, X., Miller, J.W., Mankodi, A., Kanadia, R.N., Yuan, Y., Moxley, R.T., Swanson, M.S., and Thornton, C.A. (2006). Failure of MBNL1-dependent post-natal splicing transitions in myotonic dystrophy. Hum. Mol. Genet. *15*, 2087–2097.
7. Kuyumcu-Martinez, N.M., Wang, G.S., and Cooper, T.A. (2007). Increased steady-state levels of CUGBP1 in myotonic dystrophy 1 are due to PKC-mediated hyperphosphorylation. Mol. Cell *28*, 68–78.
8. Ravel-Chapuis, A., Bélanger, G., Yadava, R.S., Mahadevan, M.S., DesGroseillers, L., Côté, J., and Jasmin, B.J. (2012). The RNA-binding protein Staufen1 is increased in DM1 skeletal muscle and promotes alternative pre-mRNA splicing. J. Cell Biol. *196*, 699–712.
9. Li, M., Zhuang, Y., Batra, R., Thomas, J.D., Li, M., Nutter, C.A., Scotti, M.M., Carter, H.A., Wang, Z.J., Huang, X.S., et al. (2020). HNRNPA1-induced spliceopathy in a transgenic mouse model of myotonic dystrophy. Proc. Natl. Acad. Sci. USA *117*, 5472–5477.
10. Mérien, A., Tahraoui-Bories, J., Cailleret, M., Dupont, J.B., Leteur, C., Polentes, J., Carteron, A., Polvèche, H., Concordet, J.P., Pinset, C., et al. (2021). CRISPR gene editing in pluripotent stem cells reveals the function of MBNL proteins during human in vitro myogenesis. Hum. Mol. Genet. *31*, 41–56.
11. López-Martínez, A., Soblechero-Martín, P., de-la-Puente-Ovejero, L., Nogales-Gadea, G., and Arechavala-Gomeza, V. (2020). An Overview of Alternative Splicing Defects Implicated in Myotonic Dystrophy Type I. Genes *11*, 1109.
12. Konieczny, P., Stepniak-Konieczna, E., and Sobczak, K. (2014). MBNL proteins and their target RNAs, interaction and splicing regulation. Nucleic Acids Res. *42*, 10873–10887.
13. Charizanis, K., Lee, K.Y., Batra, R., Goodwin, M., Zhang, C., Yuan, Y., Shiue, L., Cline, M., Scotti, M.M., Xia, G., et al. (2012). Muscleblind-like 2-mediated alternative splicing in the developing brain and dysregulation in myotonic dystrophy. Neuron *75*, 437–450.
14. Poulos, M.G., Batra, R., Li, M., Yuan, Y., Zhang, C., Darnell, R.B., and Swanson, M.S. (2013). Progressive impairment of muscle regeneration in muscleblind-like 3 isoform knockout mice. Hum. Mol. Genet. *22*, 3547–3558.
15. Choi, J., Dixon, D.M., Dansithong, W., Abdallah, W.F., Roos, K.P., Jordan, M.C., Trac, B., Lee, H.S., Comai, L., and Reddy, S. (2016). Muscleblind-like 3 deficit results in a spectrum of age-associated pathologies observed in myotonic dystrophy. Sci. Rep. *6*, 30999.
16. Spruce, T., Plass, M., Gohr, A., Ray, D., Martínez de Lagrán, M., Rot, G., Nóvoa, A., Burguera, D., Permanyer, J., Miret, M., et al. (2022). The X-linked splicing regulator MBNL3 has been co-opted to restrict placental growth in eutherians. PLoS Biol. *20*, e3001615.
17. Chamberlain, C.M., and Ranum, L.P.W. (2012). Mouse model of muscleblind-like 1 overexpression: skeletal muscle effects and therapeutic promise. Hum. Mol. Genet. *21*, 4645–4654.

18. Kanadia, R.N., Shin, J., Yuan, Y., Beattie, S.G., Wheeler, T.M., Thornton, C.A., and Swanson, M.S. (2006). Reversal of RNA missplicing and myotonia after muscleblind overexpression in a mouse poly(CUG) model for myotonic dystrophy. Proc. Natl. Acad. Sci. USA *103*, 11748–11753.

19. Chen, G., Masuda, A., Konishi, H., Ohkawara, B., Ito, M., Kinoshita, M., Kiyama, H., Matsuura, T., and Ohno, K. (2016). Phenylbutazone induces expression of MBNL1 and suppresses formation of MBNL1-CUG RNA foci in a mouse model of myotonic dystrophy. Sci. Rep. *6*, 25317.

20. Cerro-Herreros, E., Sabater-Arcis, M., Fernandez-Costa, J.M., Moreno, N., Perez-Alonso, M., Llamusi, B., and Artero, R. (2018). miR-23b and miR-218 silencing increase Muscleblind-like expression and alleviate myotonic dystrophy phenotypes in mammalian models. Nat. Commun. *9*, 2482.

21. Krützfeldt, J., Rajewsky, N., Braich, R., Rajeev, K.G., Tuschl, T., Manoharan, M., and Stoffel, M. (2005). Silencing of microRNAs in vivo with 'antagomirs. Nature *438*, 685–689.

22. Stenvang, J., Petri, A., Lindow, M., Obad, S., and Kauppinen, S. (2012). Inhibition of microRNA function by antimiR oligonucleotides. Silence *3*, 1.

23. Cerro-Herreros, E., González-Martínez, I., Moreno-Cervera, N., Overby, S., Pérez-Alonso, M., Llamusí, B., and Artero, R. (2020). Therapeutic Potential of AntagomiR-23b for Treating Myotonic Dystrophy. Mol. Ther. Nucleic Acids *21*, 837–849.

24. Cerro-Herreros, E., González-Martínez, I., Moreno, N., Espinosa-Espinosa, J., Fernández-Costa, J.M., Colom-Rodrigo, A., Overby, S.J., Seoane-Miraz, D., Poyatos-García, J., Vilchez, J.J., et al. (2021). Preclinical characterization of antagomiR-218 as a potential treatment for myotonic dystrophy. Mol. Ther. Nucleic Acids *26*, 174–191.

25. Khvorova, A., and Watts, J.K. (2017). The chemical evolution of oligonucleotide therapies of clinical utility. Nat. Biotechnol. *35*, 238–248.

26. Juliano, R.L. (2016). The delivery of therapeutic oligonucleotides. Nucleic Acids Res. *44*, 6518–6548.

27. Juliano, R.L. (2018). Intracellular Trafficking and Endosomal Release of Oligonucleotides: What We Know and What We Don't. Nucleic Acid Therapeut. *28*, 166–177.

28. Hammond, S.M., Aartsma-Rus, A., Alves, S., Borgos, S.E., Buijsen, R.A.M., Collin, R.W.J., Covello, G., Denti, M.A., Desviat, L.R., Echevarría, L., et al. (2021). Delivery of oligonucleotide-based therapeutics: challenges and opportunities. EMBO Mol. Med. *13*, e13243.

29. Betts, C.A., Saleh, A.F., Carr, C.A., Hammond, S.M., Coenen-Stass, A.M.L., Godfrey, C., McClorey, G., Varela, M.A., Roberts, T.C., Clarke, K., et al. (2015). Prevention of exercised induced cardiomyopathy following Pip-PMO treatment in dystrophic mdx mice. Sci. Rep. *5*, 8986.

30. Hammond, S.M., Hazell, G., Shabanpoor, F., Saleh, A.F., Bowerman, M., Sleigh, J.N., Meijboom, K.E., Zhou, H., Muntoni, F., Talbot, K., et al. (2016). Systemic peptide-mediated oligonucleotide therapy improves long-term survival in spinal muscular atrophy. Proc. Natl. Acad. Sci. USA *113*, 10962–10967.

31. Erazo-Oliveras, A., Muthukrishnan, N., Baker, R., Wang, T.Y., and Pellois, J.P. (2012). Improving the endosomal escape of cell-penetrating peptides and their cargos: strategies and challenges. Pharmaceuticals *5*, 1177–1209.

32. McClorey, G., and Banerjee, S. (2018). Cell-Penetrating Peptides to Enhance Delivery of Oligonucleotide-Based Therapeutics. Biomedicines *6*, 51.

33. Al Shaer, D., Al Musaimi, O., Albericio, F., and de la Torre, B.G. (2022). FDA TIDES (Peptides and Oligonucleotides) Harvest. Pharmaceuticals *15*, 222.

34. Klein, A.F., Varela, M.A., Arandel, L., Holland, A., Naouar, N., Arzumanov, A., Seoane, D., Revillod, L., Bassez, G., Ferry, A., et al. (2019). Peptide-conjugated oligonucleotides evoke long-lasting myotonic dystrophy correction in patient-derived cells and mice. J. Clin. Invest. *129*, 4739–4744.

35. Winkelsas, A.M., Grunseich, C., Harmison, G.G., Chwalenia, K., Rinaldi, C., Hammond, S.M., Johnson, K., Bowerman, M., Arya, S., Talbot, K., et al. (2021). Targeting the 5' untranslated region of SMN2 as a therapeutic strategy for spinal muscular atrophy. Mol. Ther. Nucleic Acids *23*, 731–742.

36. Tünnemann, G., Ter-Avetisyan, G., Martin, R.M., Stöckl, M., Herrmann, A., and Cardoso, M.C. (2008). Live-cell analysis of cell penetration ability and toxicity of oligo-arginines. J. Pept. Sci. *14*, 469–476.

37. Deprey, K., Batistatou, N., and Kritzer, J.A. (2020). A critical analysis of methods used to investigate the cellular uptake and subcellular localization of RNA therapeutics. Nucleic Acids Res. *48*, 7623–7639.

38. Arandel, L., Polay Espinoza, M., Matloka, M., Bazinet, A., De Dea Diniz, D., Naouar, N., Rau, F., Jollet, A., Edom-Vovard, F., Mamchaoui, K., et al. (2017). Immortalized human myotonic dystrophy muscle cell lines to assess therapeutic compounds. Dis. Model. Mech. *10*, 487–497.

39. Fus-Kujawa, A., Prus, P., Bajdak-Rusinek, K., Teper, P., Gawron, K., Kowalczuk, A., and Sieron, A.L. (2021). An Overview of Methods and Tools for Transfection of Eukaryotic Cells in vitro. Front. Bioeng. Biotechnol. *9*, 701031.

40. Keller, A., Gröger, L., Tschernig, T., Solomon, J., Laham, O., Schaum, N., Wagner, V., Kern, F., Schmartz, G.P., Li, Y., et al. (2022). miRNATissueAtlas2: an update to the human miRNA tissue atlas. Nucleic Acids Res. *50*, D211–D221.

41. Kavakiotis, I., Alexiou, A., Tastsoglou, S., Vlachos, I.S., and Hatzigeorgiou, A.G. (2022). DIANA-miTED: a microRNA tissue expression database. Nucleic Acids Res. *50*, D1055–D1061.

42. Rice, P., Longden, I., and Bleasby, A. (2000). EMBOSS: The European Molecular Biology Open Software Suite. Trends Genet. *16*, 276–277.

43. Quinlan, A.R., and Hall, I.M. (2010). BEDTools: a flexible suite of utilities for comparing genomic features. Bioinformatics *26*, 841–842.

44. Overby, S.J., Cerro-Herreros, E., Espinosa-Espinosa, J., González-Martínez, I., Moreno, N., Fernández-Costa, J.M., Balaguer-Trias, J., Ramón-Azcón, J., Pérez-Alonso, M., Møller, T., et al. (2023). BlockmiR AONs as Site-Specific Therapeutic MBNL Modulation in Myotonic Dystrophy 2D and 3D Muscle Cells and HSALR Mice. Pharmaceutics *15*, 1118.

45. Vihola, A., Bassez, G., Meola, G., Zhang, S., Haapasalo, H., Paetau, A., Mancinelli, E., Rouche, A., Hogrel, J.Y., Laforêt, P., et al. (2003). Histopathological differences of myotonic dystrophy type 1 (DM1) and PROMM/DM2. Neurology *60*, 1854–1857.

46. Shen, X., and Corey, D.R. (2018). Chemistry, mechanism and clinical status of antisense oligonucleotides and duplex RNAs. Nucleic Acids Res. *46*, 1584–1600.

47. Roberts, T.C., Langer, R., and Wood, M.J.A. (2020). Advances in oligonucleotide drug delivery. Nat. Rev. Drug Discov. *19*, 673–694.

48. Tuttle, C.S.L., Thang, L.A.N., and Maier, A.B. (2020). Markers of inflammation and their association with muscle strength and mass: A systematic review and meta-analysis. Ageing Res. Rev. *64*, 101185.

49. Chen, Y.Y., Kao, T.W., Chiu, Y.L., Peng, T.C., Yang, H.F., and Chen, W.L. (2021). Association Between Interleukin-12 and Sarcopenia. J. Inflamm. Res. *14*, 2019–2029.

50. Rodriguez, J., Vernus, B., Chelh, I., Cassar-Malek, I., Gabillard, J.C., Hadj Sassi, A., Seiliez, I., Picard, B., and Bonnieu, A. (2014). Myostatin and the skeletal muscle atrophy and hypertrophy signaling pathways. Cell. Mol. Life Sci. *71*, 4361–4371.

51. Pegoraro, V., Cudia, P., Baba, A., and Angelini, C. (2020). MyomiRNAs and myostatin as physical rehabilitation biomarkers for myotonic dystrophy. Neurol. Sci. *41*, 2953–2960.

52. Geary, R.S., Norris, D., Yu, R., and Bennett, C.F. (2015). Pharmacokinetics, biodistribution and cell uptake of antisense oligonucleotides. Adv. Drug Deliv. Rev. *87*, 46–51.

53. Bizot, F., Vulin, A., and Goyenvalle, A. (2020). Current Status of Antisense Oligonucleotide-Based Therapy in Neuromuscular Disorders. Drugs *80*, 1397–1415.

54. Braz, S.O., Acquaire, J., Gourdon, G., and Gomes-Pereira, M. (2018). Of Mice and Men: Advances in the Understanding of Neuromuscular Aspects of Myotonic Dystrophy. Front. Neurol. *9*, 519.

55. Sznajder, Ł.J., Scotti, M.M., Shin, J., Taylor, K., Ivankovic, F., Nutter, C.A., Aslam, F.N., Subramony, S.H., Ranum, L.P.W., and Swanson, M.S. (2020). Loss of MBNL1 induces RNA misprocessing in the thymus and peripheral blood. Nat. Commun. *11*, 2022.

56. Song, K.Y., Guo, X.M., Wang, H.Q., Zhang, L., Huang, S.Y., Huo, Y.C., Zhang, G., Feng, J.Z., Zhang, R.R., Ma, Y., et al. (2020). MBNL1 reverses the proliferation defect of skeletal muscle satellite cells in myotonic dystrophy type 1 by inhibiting autophagy via the mTOR pathway. Cell Death Dis. *11*, 545.

57. Pandey, S.K., Wheeler, T.M., Justice, S.L., Kim, A., Younis, H.S., Gattis, D., Jauvin, D., Puymirat, J., Swayze, E.E., Freier, S.M., et al. (2015). Identification and characterization of modified antisense oligonucleotides targeting DMPK in mice and nonhuman primates for the treatment of myotonic dystrophy type 1. J. Pharmacol. Exp. Therapeut. *355*, 329–340.

58. Bailey, J.K., Shen, W., Liang, X.H., and Crooke, S.T. (2017). Nucleic acid binding proteins affect the subcellular distribution of phosphorothioate antisense oligonucleotides. Nucleic Acids Res. *45*, 10649–10671.

59. Gilleron, J., Querbes, W., Zeigerer, A., Borodovsky, A., Marsico, G., Schubert, U., Manygoats, K., Seifert, S., Andree, C., Stöter, M., et al. (2013). Image-based analysis of lipid nanoparticle-mediated siRNA delivery, intracellular trafficking and endosomal escape. Nat. Biotechnol. *31*, 638–646.

60. van der Bent, M.L., Paulino da Silva Filho, O., Willemse, M., Hällbrink, M., Wansink, D.G., and Brock, R. (2019). The nuclear concentration required for antisense oligonucleotide activity in myotonic dystrophy cells. Faseb. J. *33*, 11314–11325.

61. Segal, M., Biscans, A., Gilles, M.E., Anastasiadou, E., De Luca, R., Lim, J., Khvorova, A., and Slack, F.J. (2020). Hydrophobically Modified let-7b miRNA Enhances Biodistribution to NSCLC and Downregulates HMGA2 In Vivo. Mol. Ther. Nucleic Acids *19*, 267–277.

62. Foged, C., and Nielsen, H.M. (2008). Cell-penetrating peptides for drug delivery across membrane barriers. Expet Opin. Drug Deliv. *5*, 105–117.

63. Pooga, M., and Langel, Ü. (2015). Classes of Cell-Penetrating Peptides. Methods Mol. Biol. *1324*, 3–28.

64. Lehto, T., Ezzat, K., Wood, M.J.A., and El Andaloussi, S. (2016). Peptides for nucleic acid delivery. Adv. Drug Deliv. Rev. *106*, 172–182.

65. Cleal, K., He, L., Watson, P.D., and Jones, A.T. (2013). Endocytosis, intracellular traffic and fate of cell penetrating peptide based conjugates and nanoparticles. Curr. Pharmaceut. Des. *19*, 2878–2894.

66. Gait, M.J., Arzumanov, A.A., McClorey, G., Godfrey, C., Betts, C., Hammond, S., and Wood, M.J.A. (2019). Cell-Penetrating Peptide Conjugates of Steric Blocking Oligonucleotides as Therapeutics for Neuromuscular Diseases from a Historical Perspective to Current Prospects of Treatment. Nucleic Acid Therapeut. *29*, 1–12.

67. Zou, L.L., Ma, J.L., Wang, T., Yang, T.B., and Liu, C.B. (2013). Cell-penetrating Peptide-mediated therapeutic molecule delivery into the central nervous system. Curr. Neuropharmacol. *11*, 197–208.

68. Bersani, M., Rizzuti, M., Pagliari, E., Garbellini, M., Saccomanno, D., Moulton, H.M., Bresolin, N., Comi, G.P., Corti, S., and Nizzardo, M. (2022). Cell-penetrating peptide-conjugated Morpholino rescues SMA in a symptomatic preclinical model. Mol. Ther. *30*, 1288–1299.

69. Tanner, M.K., Tang, Z., and Thornton, C.A. (2021). Targeted splice sequencing reveals RNA toxicity and therapeutic response in myotonic dystrophy. Nucleic Acids Res. *49*, 2240–2254.

70. Bartel, D.P. (2018). Metazoan MicroRNAs. Cell *173*, 20–51.

71. Ho, T.H., Charlet-B, N., Poulos, M.G., Singh, G., Swanson, M.S., and Cooper, T.A. (2004). Muscleblind proteins regulate alternative splicing. EMBO J. *23*, 3103–3112.

72. Wang, E.T., Cody, N.A.L., Jog, S., Biancolella, M., Wang, T.T., Treacy, D.J., Luo, S., Schroth, G.P., Housman, D.E., Reddy, S., et al. (2012). Transcriptome-wide regulation of pre-mRNA splicing and mRNA localization by muscleblind proteins. Cell *150*, 710–724.

73. Batra, R., Manchanda, M., and Swanson, M.S. (2015). Global insights into alternative polyadenylation regulation. RNA Biol. *12*, 597–602.

74. Rau, F., Freyermuth, F., Fugier, C., Villemin, J.P., Fischer, M.C., Jost, B., Dembele, D., Gourdon, G., Nicole, A., Duboc, D., et al. (2011). Misregulation of miR-1 processing is associated with heart defects in myotonic dystrophy. Nat. Struct. Mol. Biol. *18*, 840–845.

75. Masuda, A., Andersen, H.S., Doktor, T.K., Okamoto, T., Ito, M., Andresen, B.S., and Ohno, K. (2012). CUGBP1 and MBNL1 preferentially bind to 3' UTRs and facilitate mRNA decay. Sci. Rep. *2*, 209.

76. Wu, D.R., Gu, K.L., Yu, J.C., Fu, X., Wang, X.W., Guo, W.T., Liao, L.Q., Zhu, H., Zhang, X.S., Hui, J., and Wang, Y. (2018). Opposing roles of miR-294 and MBNL1/2 in shaping the gene regulatory network of embryonic stem cells. EMBO Rep. *19*, e45657.

77. Choi, J., Personius, K.E., DiFranco, M., Dansithong, W., Yu, C., Srivastava, S., Dixon, D.M., Bhatt, D.B., Comai, L., Vergara, J.L., and Reddy, S. (2015). Muscleblind-Like 1 and Muscleblind-Like 3 Depletion Synergistically Enhances Myotonia by Altering Clc-1 RNA Translation. EBioMedicine *2*, 1034–1047.

78. Adereth, Y., Dammai, V., Kose, N., Li, R., and Hsu, T. (2005). RNA-dependent integrin alpha3 protein localization regulated by the Muscleblind-like protein MLP1. Nat. Cell Biol. *7*, 1240–1247.

79. Wheeler, T.M., Lueck, J.D., Swanson, M.S., Dirksen, R.T., and Thornton, C.A. (2007). Correction of ClC-1 splicing eliminates chloride channelopathy and myotonia in mouse models of myotonic dystrophy. J. Clin. Invest. *117*, 3952–3957.

80. Najjar, K., Erazo-Oliveras, A., Mosior, J.W., Whitlock, M.J., Rostane, I., Cinclair, J.M., and Pellois, J.P. (2017). Unlocking Endosomal Entrapment with Supercharged Arginine-Rich Peptides. Bioconjugate Chem. *28*, 2932–2941.

81. Poon, G.M.K., and Gariépy, J. (2007). Cell-surface proteoglycans as molecular portals for cationic peptide and polymer entry into cells. Biochem. Soc. Trans. *35*, 788–793.

82. Cordier, C., Boutimah, F., Bourdeloux, M., Dupuy, F., Met, E., Alberti, P., Loll, F., Chassaing, G., Burlina, F., and Saison-Behmoaras, T.E. (2014). Delivery of antisense peptide nucleic acids to cells by conjugation with small arginine-rich cell-penetrating peptide (R/W)9. PLoS One *9*, e104999.

83. Torres, A.G., Fabani, M.M., Vigorito, E., Williams, D., Al-Obaidi, N., Wojciechowski, F., Hudson, R.H.E., Seitz, O., and Gait, M.J. (2012). Chemical structure requirements and cellular targeting of microRNA-122 by peptide nucleic acids anti-miRs. Nucleic Acids Res. *40*, 2152–2167.

84. Seth, P.P., Tanowitz, M., and Bennett, C.F. (2019). Selective tissue targeting of synthetic nucleic acid drugs. J. Clin. Invest. *129*, 915–925.

85. Gökirmak, T., Nikan, M., Wiechmann, S., Prakash, T.P., Tanowitz, M., and Seth, P.P. (2021). Overcoming the challenges of tissue delivery for oligonucleotide therapeutics. Trends Pharmacol. Sci. *42*, 588–604.

86. Stoodley, J., Miraz, D.S., Jad, Y., Fischer, M., Wood, M.J.A., and Varela, M.A. (2023). Peptide-Conjugated PMOs for the Treatment of Myotonic Dystrophy. Methods Mol. Biol. *2587*, 209–237.

87. Moreno, N., González-Martínez, I., Artero, R., and Cerro-Herreros, E. (2022). Rapid Determination of MBNL1 Protein Levels by Quantitative Dot Blot for the Evaluation of Antisense Oligonucleotides in Myotonic Dystrophy Myoblasts. Methods Mol. Biol. *2434*, 207–215.

88. Tian, G., Tang, F., Yang, C., Zhang, W., Bergquist, J., Wang, B., Mi, J., and Zhang, J. (2017). Quantitative dot blot analysis (QDB), a versatile high throughput immunoblot method. Oncotarget *8*, 58553–58562.

89. Burki, U., Keane, J., Blain, A., O'Donovan, L., Gait, M.J., Laval, S.H., and Straub, V. (2015). Development and Application of an Ultrasensitive Hybridization-Based ELISA Method for the Determination of Peptide-Conjugated Phosphorodiamidate Morpholino Oligonucleotides. Nucleic Acid Therapeut. *25*, 275–284.

90. Mankodi, A., Logigian, E., Callahan, L., McClain, C., White, R., Henderson, D., Krym, M., and Thornton, C.A. (2000). Myotonic dystrophy in transgenic mice expressing an expanded CUG repeat. Science *289*, 1769–1773.

OMTN, Volume *34*

Supplemental information

Peptide-conjugated antimiRs improve myotonic dystrophy type 1 phenotypes by promoting endogenous MBNL1 expression

Irene González-Martínez, Estefanía Cerro-Herreros, Nerea Moreno, Andrea García-Rey, Jorge Espinosa-Espinosa, Marc Carrascosa-Sàez, Diego Piqueras-Losilla, Andrey Arzumanov, David Seoane-Miraz, Yahya Jad, Richard Raz, Matthew J. Wood, Miguel A. Varela, Beatriz Llamusí, and Rubén Artero

Supplemental Information

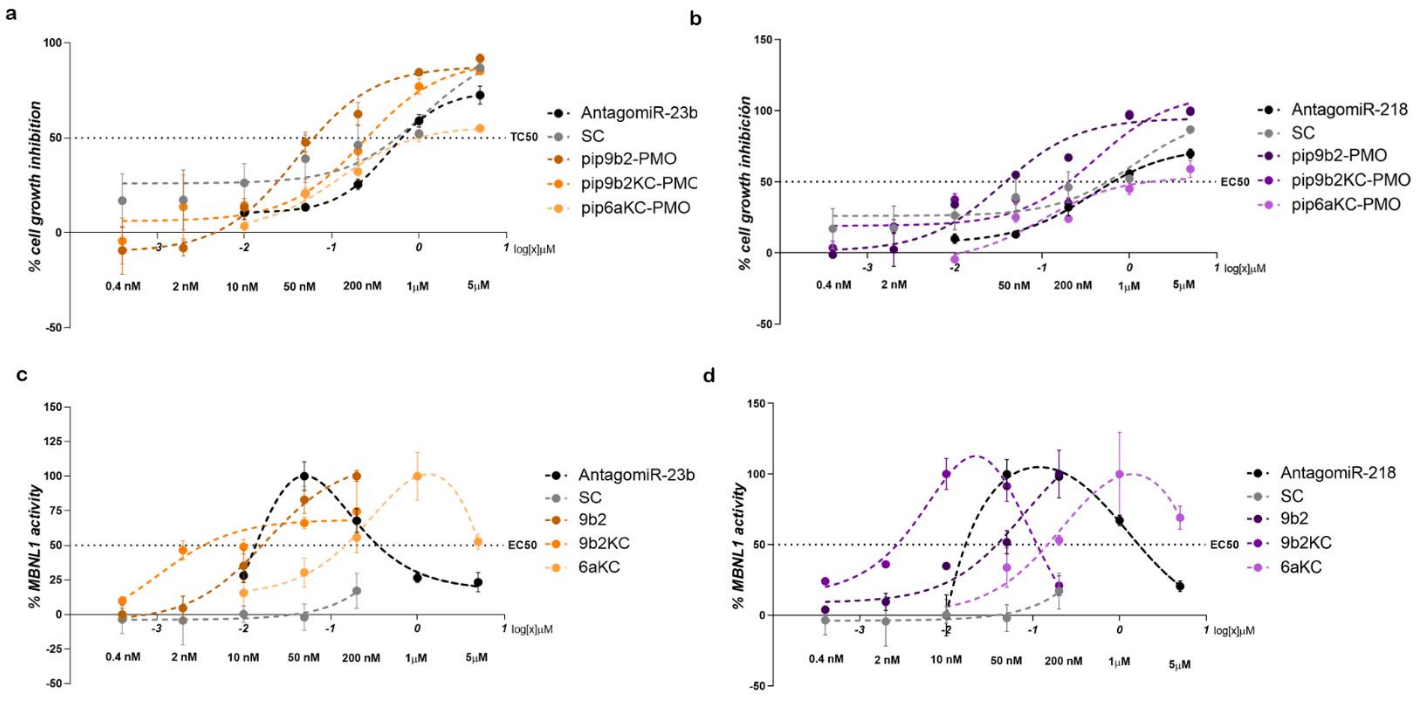

Figure S1 CPP-PMO antimiRs promote MBNL1 expression in DM1 cells. (**a**, **b**) Percentage of cell growth inhibition of DM1 cells after lipofection at different concentrations with ONs against (**a**) miR-23b and (**b**) miR-218. Dotted lines indicate TC_{50} value. (**c**, **d**) Percentage of MBNL1 expression increase (100% equals maximum increase) in DM1 cells after lipofection at the indicated concentrations with ONs against (**c**) miR-23b and (**d**) miR-218. Dotted lines indicate EC_{50} value. Error bars indicate mean ± SEM.

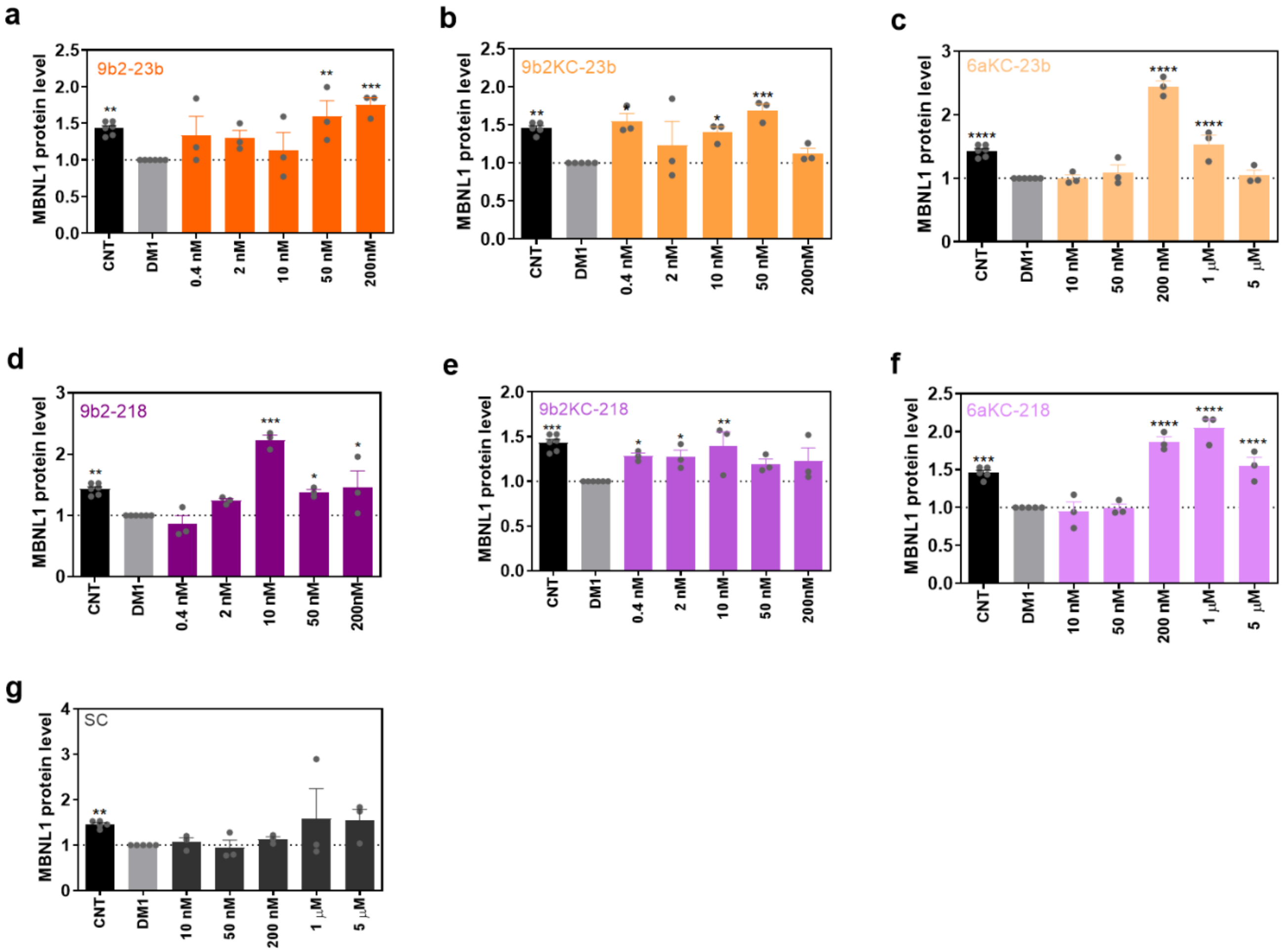

Figure S2 Activity effect of CPP-PMO antimiRs in DM cells by Gymnotic Delivery. Data shows the quantification by QDB of MBNL1 protein levels after treatment with different ONs in DM1 cells at the indicated concentrations: (**a**) 9b2-23b, (**b**) 9b2KC-23b, (**c**) 6aKC-23b, (**d**) 9b2-218, (**e**) 9b2KC-218, (**f**) 6aKC-218 and (**g**) scrambled control (SC). Black bars represent MBNL1 levels of unaffected control (CNT) myotubes and light grey bars represent MBNL1 levels in DM1 mock-treated cells. Dotted line indicates MBNL1 levels in DM1 cells without treatment. *: $p<0.05$, **: $p<0.01$, ***: $p<0.001$ according to one-way ANOVA compared to DM1 non-treated cells. Each concentration was tested in triplicate. Individual values are indicated as datapoints. Error bars indicate mean ± SEM.

a

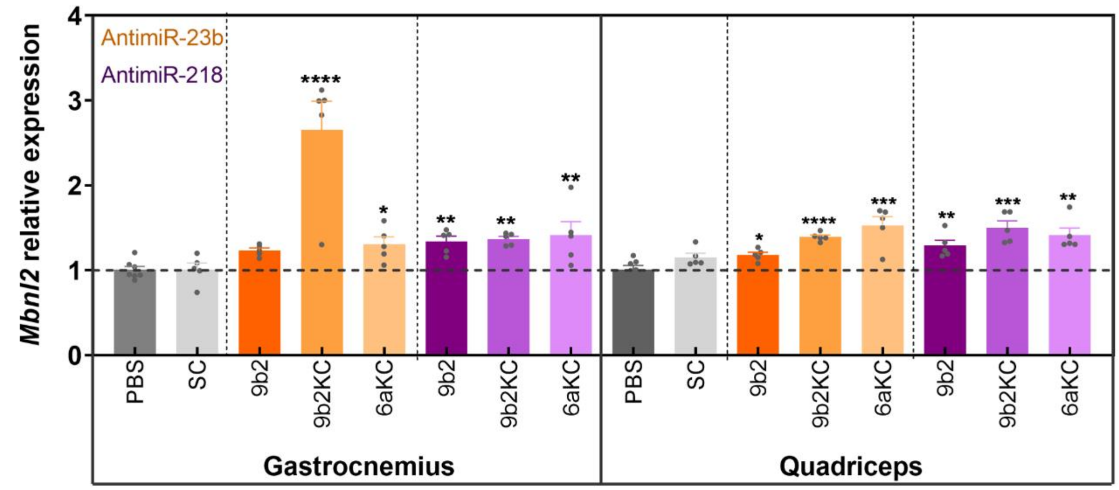

b

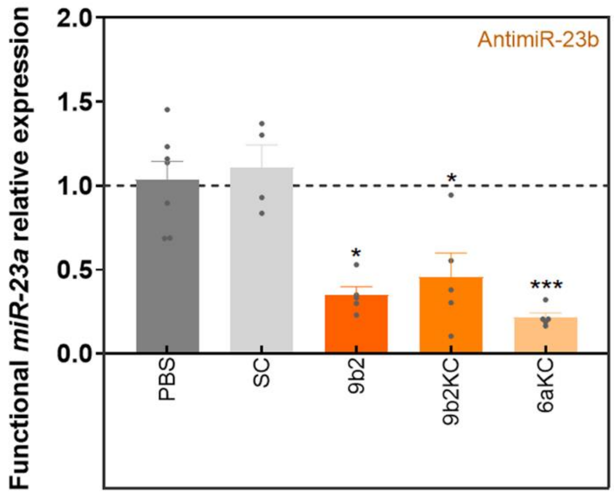

microRNA	Sequence	Max Score	Query Cover	E Value	Percent Identity	Accession Length
hsa-miR-23b-3p	AUCACAUUGCCAGGGAUUACCAC	-	-	-	-	23
hsa-miR-23a-3p	AUCACAUUGCCAGGGAUUUCC	36.2	78%	4.00E-09	87%	21

Figure S3 Administration of CPP-PMO antimiRs increases *Mbnl2* and decreases *miR-23a* expression in HSA^{LR} mice. After 45 days of treatment with CPP-PMOs, gastrocnemius and quadriceps muscles were dissected to measure (**a**) *Mbnl2* transcript levels relative to *Gapdh* endogenous control and (**b**) *miR-23a* functional levels relative to U1 and U6 snRNA endogenous controls. An alignment of miR-23b and miR-23a is shown. Statistical comparisons were performed in each case against PBS-treated *HSA^{LR}* values (indicated by a black dashed line) with Student's

t-tests. *: $p<0.05$, **: $p<0.01$, ***: $p<0.001$, ****: $p<0.0001$. PBS n=7, SC n=5, 9b2-218 n=5, 9b2-23b n=5, 9b2KC-218 n=5, 9b2KC-218 n=5, 6aKC-218 n=5, and 6aKC-23b n=5. Individual values are indicated as datapoints. Error bars indicate mean ± SEM.

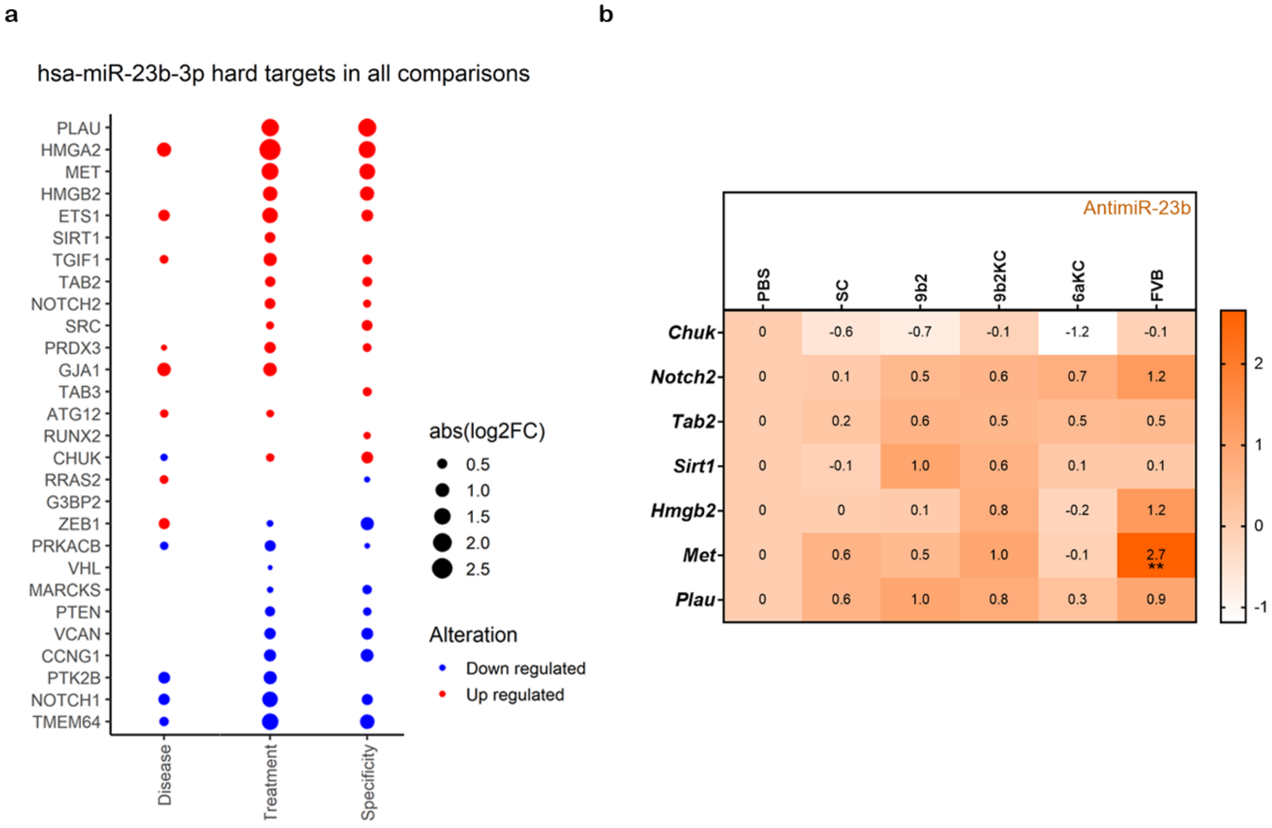

Figure S4 Assessment of effects by miR-23b blockers. (**a**) Effects on miR-23b target transcripts that previous antagomiR-23b manages to activate (red dots) or repress (blue dots). The size of each dot represents the log2 fold change of the expression of the indicated gene for the comparisons shown: DM1 untreated versus CNT healthy cells (Disease), antagomiR-23b-treated DM1 versus CNT healthy cells (Treatment), antagomiR-23b-treated DM1 versus DM1 untreated cells (Specificity). (**b**) Expression of selected genes (log2 fold change) in quadriceps muscles of CPP-PMO antimiRs-23b treated mice. Data were analysed by one-way ANOVA test compared to PBS-treated *HSA*^{LR} mice. **: p<0.01.

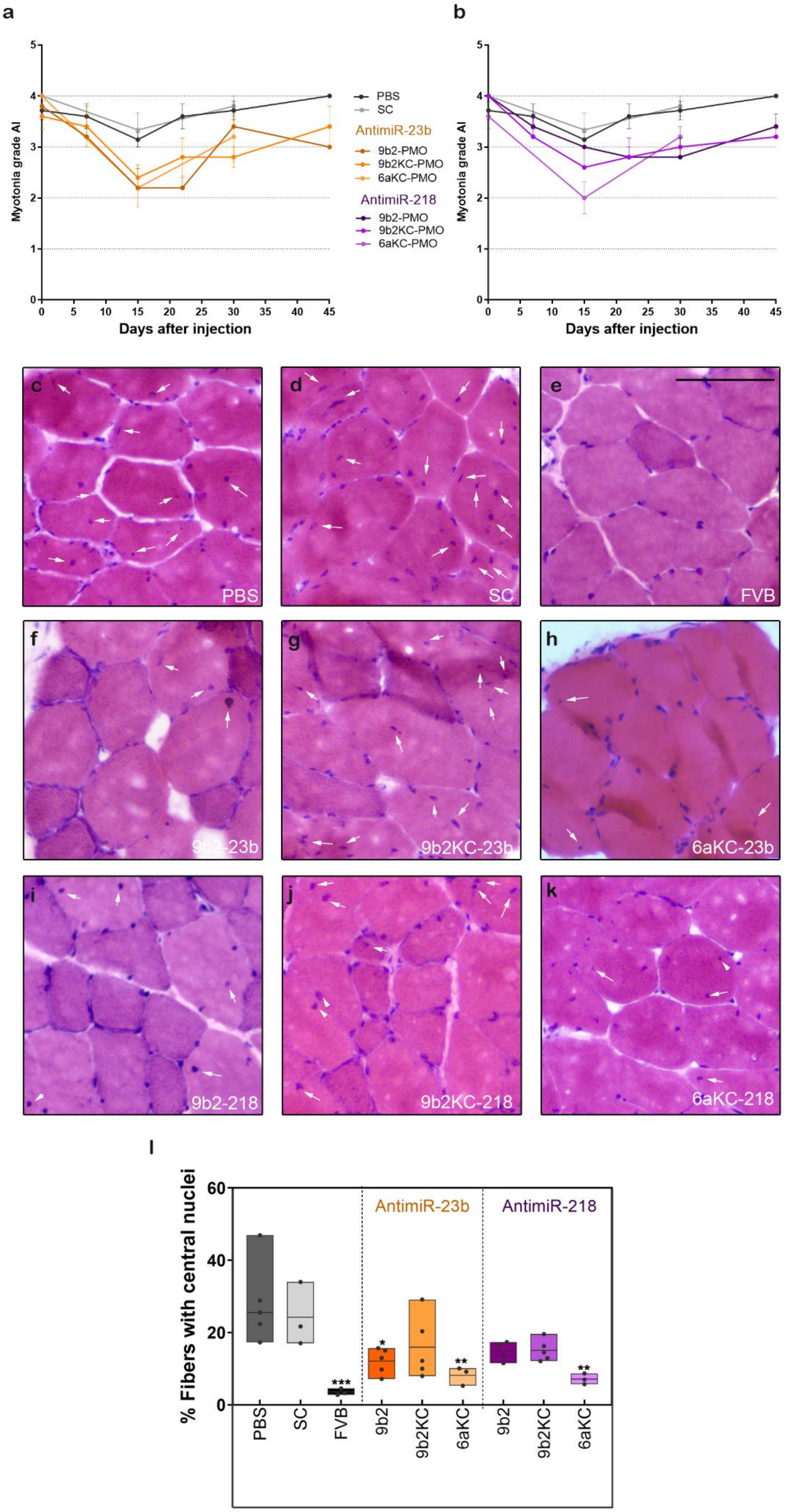
a
b
Myotonia grade AI
Days after injection
PBS
SC
AntimiR-23b
9b2-PMO
9b2KC-PMO
6aKC-PMO
AntimiR-218
9b2-PMO
9b2KC-PMO
6aKC-PMO
c
d
e
f
g
h
i
j
k
PBS
SC
FVB
9b2-23b
9b2KC-23b
6aKC-23b
9b2-218
9b2KC-218
6aKC-218
l
% Fibers with central nuclei
AntimiR-23b
AntimiR-218
PBS
SC
FVB
9b2
9b2KC
6aKC
9b2
9b2KC
6aKC

Figure S5 CPP-PMOs antimiRs rescue functional and histological alterations in HSA^{LR} mice. (**a**,**b**) Myotonia grade was measured before injection, at the indicated intermediate time points, and immediately before sacrifice. (**c**-**k**) Representative images of hematoxylin and eosin staining of gastrocnemius muscle slices from each treatment group. White arrows indicate examples of centrally located nuclei in muscle fibres. (**l**) Quantification of the percentage of muscle fibers with central nuclei in gastrocnemius. Individual values are indicated as datapoints. Error bars indicate mean ± SEM. Data were analyzed by ANOVA one-way test compared to PBS-treated HSA^{LR} mice. *: $p<0.05$, **: $p<0.01$, ***: $p<0.001$. PBS $n=7$, SC $n=5$, 9b2-218 $n=5$, 9b2-23b $n=5$, 9b2KC-218 $n=5$, 9b2KC-218 $n=5$, 6aKC-218 $n=5$, and 6aKC-23b $n=5$.

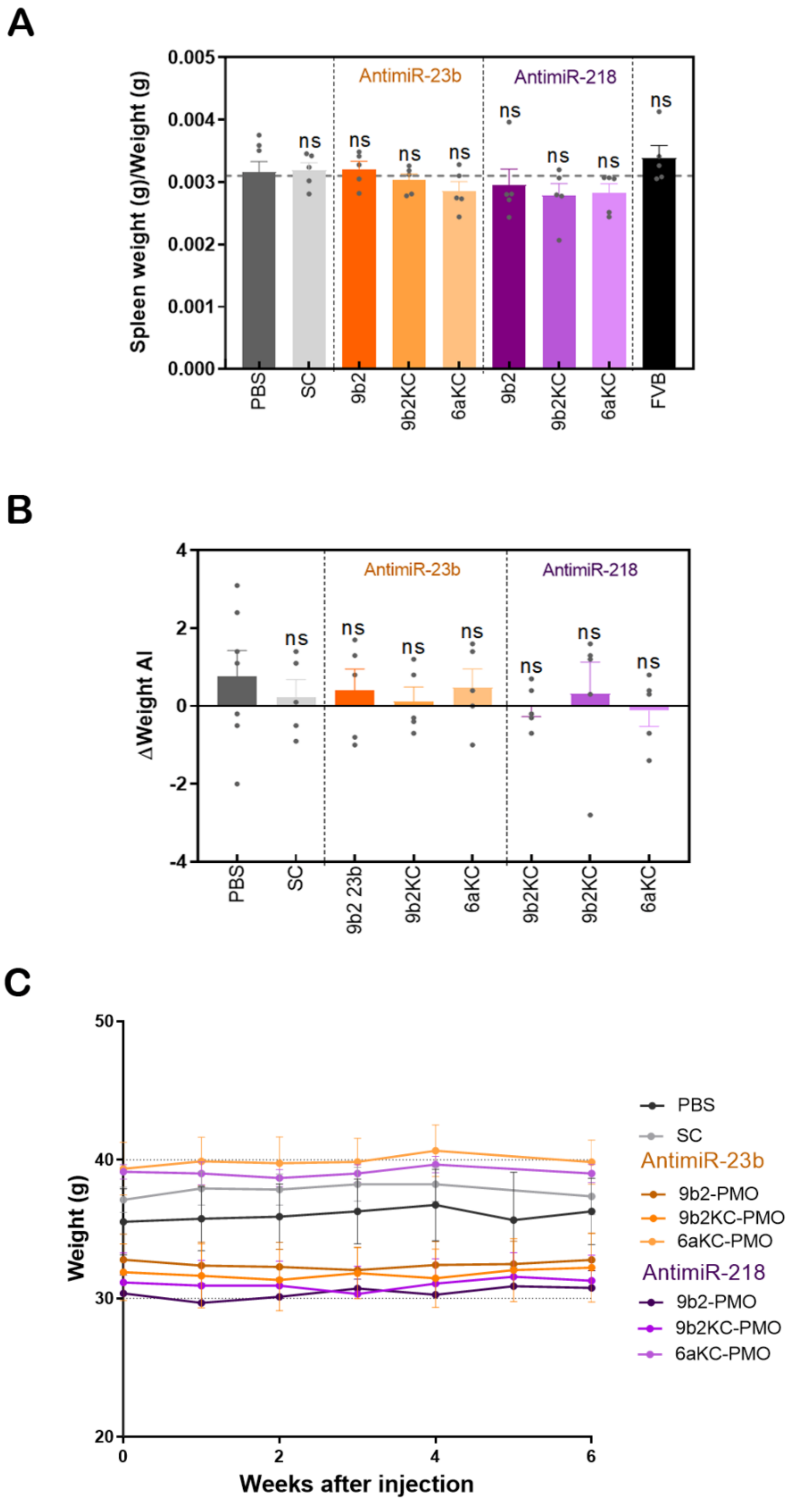
A
Spleen weight (g)/Weight (g)
AntimiR-23b
AntimiR-218
ns
PBS
SC
9b2
9b2KC
6aKC
9b2
9b2KC
6aKC
FVB
B
ΔWeight AI
AntimiR-23b
AntimiR-218
ns
PBS
SC
9b2 23b
9b2KC
6aKC
9b2KC
9b2KC
6aKC
C
Weight (g)
Weeks after injection
PBS
SC
AntimiR-23b
9b2-PMO
9b2KC-PMO
6aKC-PMO
AntimiR-218
9b2-PMO
9b2KC-PMO
6aKC-PMO

Figure S6 Potential deleterious or toxic effects in response to CPP-PMOS in HSA^{LR} mice. Mouse relative (**a**) spleen weight or (**b**) weight after treatment with PBS, SC or different CPP-PMOs. (**c**) Evolution of weight of each mouse throughout the entire experiment. Statistical analysis (one-way ANOVA) was performed compared to PBS-treated *HSA*^{LR} controls. Individual values are indicated as datapoints. Error bars indicate mean ± SEM. ns: not statistically significant.

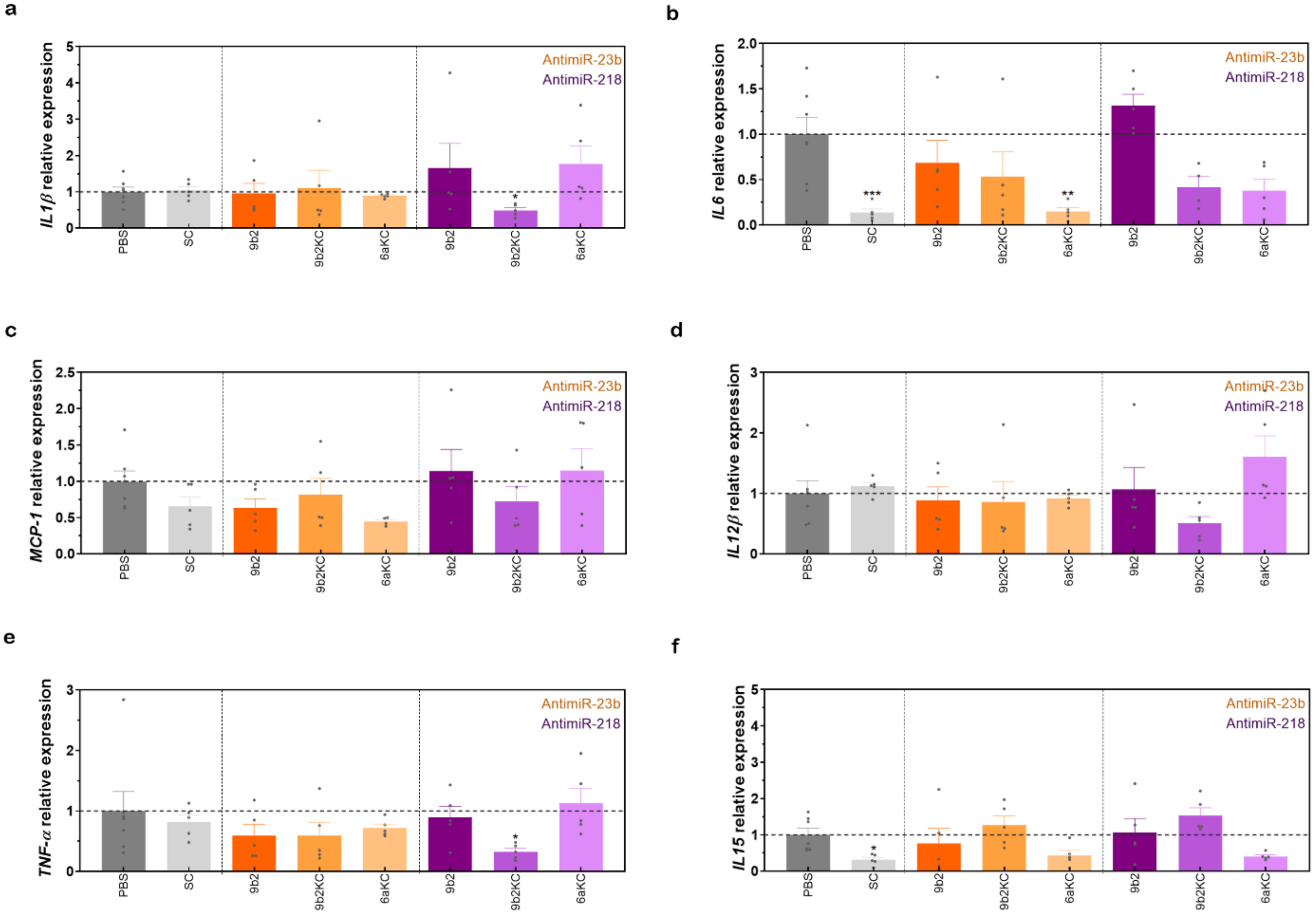

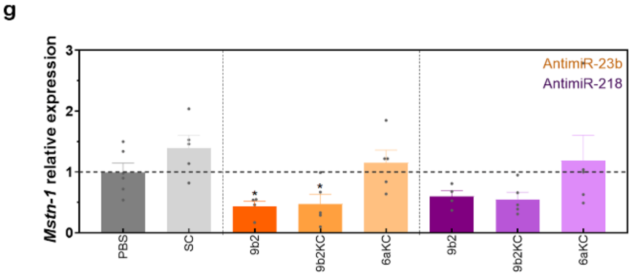

h

Qd	IL1β	IL6	MCP-1	IL12β	TNF-α	IL15	Mstn-1
SC	-	***	-	-	-	*	-
9b2-23b	-	-	-	-	-	-	*
9b2KC-23b	-	-	-	-	-	-	*
6aKC-23b	-	**	-	-	-	-	-
9b2-218	-	-	-	-	-	-	-
9b2KC-218	*	-	-	-	*	-	-
6aKC-218	-	-	-	-	-	-	-

Figure S7 CPP-PMOs antimiRs do not activate an immune response in the muscles of treated mice. Quantification by RT-qPCR of the cytokines (**a**) IL1β, (**b**) IL6, (**c**) MCP-1, (**d**) IL12β, (**e**) TNF-α, and (**f**) IL15, and **g**) muscle regeneration marker Mstn-1 in quadriceps muscles of HSA^{LR} mice treated with the designated treatment. (h) Summary table of significant changes observed. - indicates no significant change. All comparisons were to PBS-treated (indicated by a black dashed

line) mice and used ANOVA or Kruskal-Wallis test when necessary. All significant differences were towards reduction in treated muscles vs. untreated. *: p<0.05, **: p<0.01, ***: p<0.001.

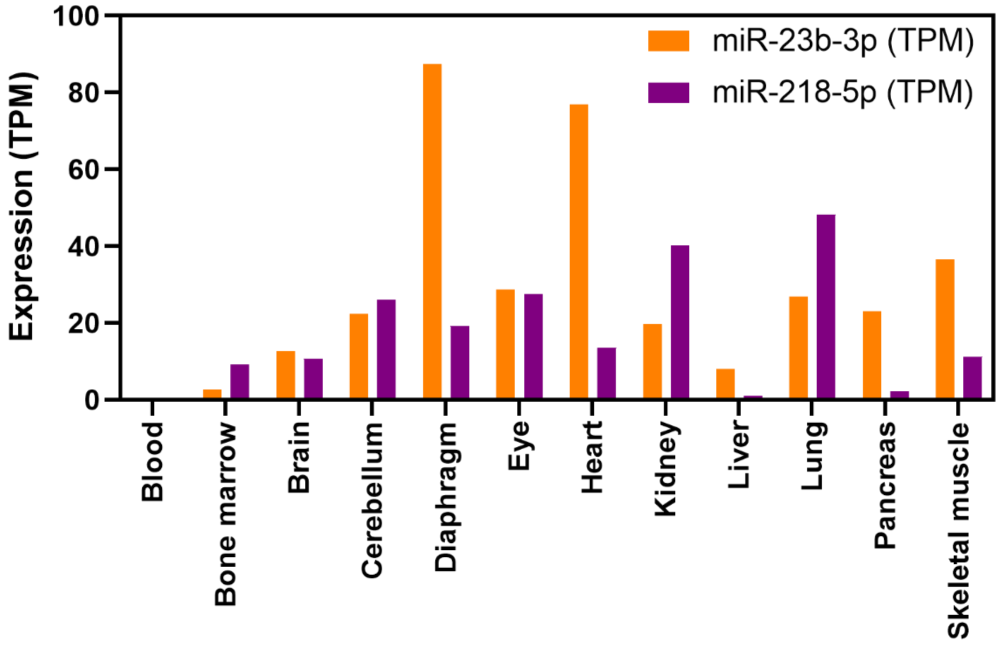

Figure S8 Expression of miR-23b and miR-218 in multiples tissues. Cap analysis gene expression data was retrieved from the ZENBU database (https://fantom.gsc.riken.jp/zenbu/) to prepare this figure. TPM: transcript per million.

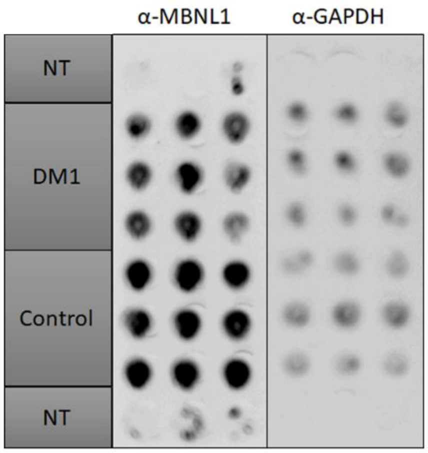

	MBNL1			GAPDH		
	1	2	3	4	5	6
Negative	2815	3097	1552	1575	1236	1395
DM1	25462	35155	39750	8125	8334	6242
	29254	51556	24689	10607	10526	9047
	29965	41118	20251	8708	7834	7103
CNT	45666	59067	44824	10384	8918	7400
	39675	48803	43641	11806	8157	9579
	47048	50495	54247	11034	9471	9794
Negative	2299	1104	1772	3260	2277	2497

Figure S9 Validation of QDB for in vitro studies. Dot blot images by ImageQuant of CNT and DM1 cells measuring MBNL1 and endogenous control GAPDH. The table shows readings of each dot obtained by Tecan plate reader.

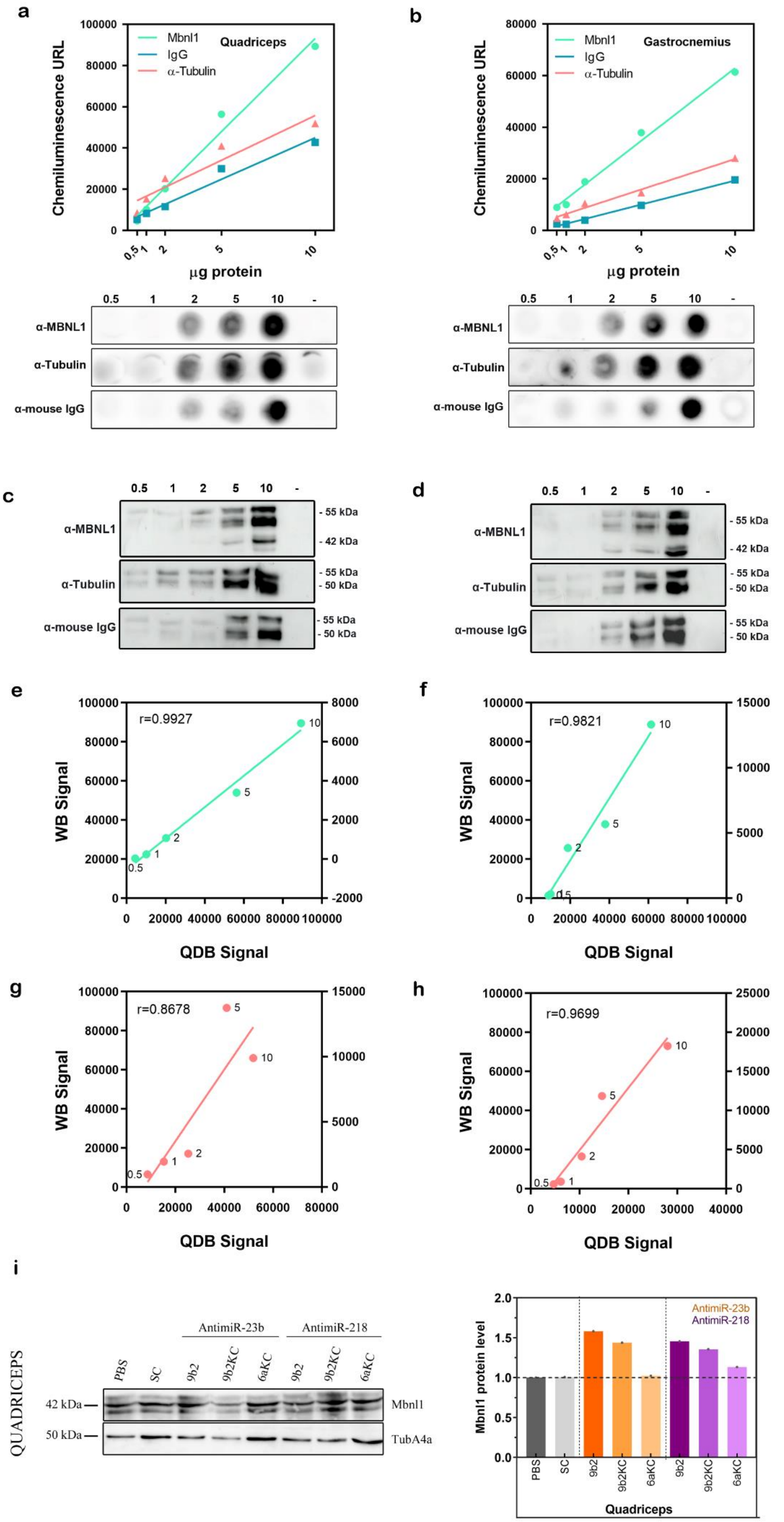
a
Mbnl1
IgG
α-Tubulin
Quadriceps
Chemiluminescence URL
μg protein
α-MBNL1
α-Tubulin
α-mouse IgG
b
Gastrocnemius
c
d
- 55 kDa
- 42 kDa
- 50 kDa
e
r=0.9927
f
r=0.9821
g
r=0.8678
h
r=0.9699
WB Signal
QDB Signal
i
QUADRICEPS
AntimiR-23b
AntimiR-218
PBS
SC
9b2
9b2KC
6aKC
42 kDa
50 kDa
Mbnl1
TubA4a
Mbnl1 protein level
Quadriceps

Figure S10 Optimization and validation of the QDB technique for quantification of Mbnl1 in murine muscle tissue. (**a**, **b**) Evaluation of specificity and lineal range of α-MBNL1, α-Tubulin and α-mouse-IgG antibodies in QDB using five protein inputs (0.5, 1, 2, 5 and 10 µg) of mouse muscle tissues samples. Representative images of QDB signal are shown underneath the curves, indicating the corresponding protein input. (**c**, **d**) Evaluation of the specificity of αMBNL1, αTubulin, and αmouseIgG antibodies in Western blot (WB) with mouse muscle samples in the same range of protein inputs. (**e**-**h**) Correlation between QDB and WB signal for (**e**, **f**) α-MBNL1 and (**g**, **h**) α-Tubulin antibodies in (**e**, **g**) quadriceps and (**f**, **h**) gastrocnemius muscles. (**i**) Representative Western blot of pools of samples from each treatment in quadriceps. -: negative control; r: Pearson coefficient of correlation.

Table S1 Top similarities for antimiR-23b and –218 sequences in human RNAs.

antimiR-23b				
Genomic position	Gen	Identity (%)	Overlap (bp)	Number of matches (bp)
chr9: 95085264-95085285	MIR-23B	100	21	21
chr9: 95085264-95085285	AOPEP	100	21	21
chr19: 13836594-13836615	MIR-23A	95.24	20	20
chrX: 20017101-20017122	MAP7D2	95.24	20	20
antimiR-218				
Genomic position	Gen	Identity (%)	Overlap (bp)	Number of matches (bp)
chr4:20528298-20528319	MIR-218-1	100	21	21
chr4:20528298-20528319	SLIT2	100	21	21
chr5:168768210-168768231	MIR-218-2	100	21	21
chr5:168768210-168768231	SLIT3	100	21	21
chr4:95432770-95432791	UNC5C	90.48	19	19
chr8:99669547-99669568	VPS13B	90.48	19	19

Table S2 Percentage spliced-in (PSI) of Nfix, Clcn1, and Atp2a1 of the indicated muscle.

		PSI					
Mice	Treatment	*Nfix* Gast	*Nfix* Quads	*Clcn1* Gast	*Clcn1* Quads	*Atp2a1* Gast	*Atp2a1* Quads
1990-1	HSA^{LR}-PBS	64.24	53.97	27.00	36.55	15.30	27.20
1995-1		55.56	71.29	24.30	30.28	30.33	21.37
1996-1		54.67	53.21	34.05	30.89	22.57	13.71
1996-2		66.55	46.33	31.36	36.72	7.55	37.47
2130-1		76.55	65.44	47.52	31.25	2.48	16.44
2710-4		67.04	77.90	28.18	33.10	12.30	18.78
2657-4		70.82	65.53	27.60	29.53	12.53	19.77
2710-1	Scramble	62.98	80.52	26.86	38.65	15.65	7.50
2657-1		76.46	72.49	36.37	28.91	6.44	18.65
2817-1		52.74	60.30	12.37	16.71	27.49	34.43
2816-1		55.70	66.59	14.67	28.33	24.15	18.71
2713-1		81.00	72.38	42.81	34.05	5.60	17.01
1990-2	9b2-218	58.31	10.51	18.06	24.88	31.61	99.28
1995-2		69.09	35.59	25.23	1.85	14.72	71.69
2032-1		29.97	30.95	5.28	4.09	85.58	99.29
2130-2		59.09	43.26	30.49	13.35	28.83	50.19
2131-1		66.06	15.39	30.67	26.96	19.19	99.72
1990-4	9b2KC-218	66.68	67.46	22.57	22.73	35.28	25.20
1995-4		62.07	21.60	29.41	2.19	12.53	99.70
2087-1		66.91	48.85	32.73	27.06	17.65	25.98
2130-4		60.18	51.19	22.25	25.84	19.44	29.31
2131-3		67.84	51.94	31.59	30.53	12.90	30.83
2710-3	6aKC-218	68.36	62.29	24.01	27.67	12.62	26.51
2657-3		74.79	66.72	28.56	33.68	14.70	21.08
2817-3		70.85	52.44	28.37	24.84	11.51	26.96
2816-3		73.58	52.77	38.26	25.74	10.78	31.16
2713-3		51.68	89.96	18.26	41.25	31.20	9.84
1990-3	9b2-23b	55.08	54.78	26.86	24.88	23.52	36.45
1995-3		47.66	23.44	11.37	1.85	52.69	98.27
2032-2		63.11	15.30	26.85	0.61	18.37	99.53
2130-3		66.38	4.40	32.69	13.35	16.01	75.32
2131-2		63.52	48.44	33.28	26.96	11.16	25.61
1990-5	9b2KC-23b	45.55	56.90	15.58	26.94	42.67	39.24
2087-2		57.17	56.46	38.58	29.66	11.22	37.72
2130-5		53.38	54.10	18.78	22.47	31.29	33.71
2032-3		57.04	66.58	26.33	19.41	33.92	28.53
2131-4		62.73	51.62	30.11	27.99	10.51	24.15
2710-2	6aKC-23b	63.59	62.29	29.03	28.33	14.74	33.87
2657-2		69.22	53.11	31.54	24.44	14.22	29.81
2817-2		47.55	56.32	15.22	30.63	26.08	23.38
2816-2		61.79	64.55	18.35	35.11	16.73	17.76
2713-2		62.43	58.42	23.17	37.69	17.96	24.03

Table S3 Serum biochemistry profile of different mice treated with CPP-PMOs antimiRs. Mean ± SEM is shown for each biomarker in the indicated treatment group. Statistical test compares CPP-PMO-treated HSA^{LR} mice with FVB controls.

Table S4 White blood cells (WBC) count for each treatment group and a compositional data dendrogram. Mean ± SEM is shown for each cell count of the indicated groups. The statistical test compares CPP-PMO-treated mice with FVB controls. In all cases treatments refer to HSA^{LR} mice treated with each CPP-PMO, Scramble or PBS. FVB controls were mock-treated with PBS. *p<0.05.

Table S5 Sequences of primers used for RT-qPCR of off-targets, interleukins and muscle regeneration marker Mstn-1 and RT-PCR of splicing events.

Gene	Forward primer	Reverse primer	Exon
**CHUK**	TCGGAAACCAGCCTCTCAGTGT	CTTCTGGATGCAAATGGTCCTTC	-
**SRC**	GTTGCTTCGGAGAGGTGTGGAT	CACCAGTTTCTCGTGCCTCAGT	-
**NOTCH2**	CCACCTGCAATGACTTCATCGG	TCGATGCAGGTGCCTCCATTCT	-
**TAB2**	CATTCAGCATCTCACAGACCCG	CTTTGAAGCCGTTCCATCCTGG	-
**SIRT1**	GGAGCAGATTAGTAAGCGGCTTG	GTTACTGCCACAGGAACTAGAGG	-
**HMGB2**	GATGTGGTCTGAGCAATCTGCC	CCTGCTTCACTTTTGCCCTTGG	-
**MET**	GTTCTGCTTGGCAACGAGAGCT	GGAGAATGCACTGTATTGCGTCG	-
**PLAU**	AGAAGCGACCCTGGTGCTATGT	CCACACTGGAAGCCTTGTTGGT	-
**IKBKB**	GCAGACTGACATTGTGGACCTG	ATCTCCTGGCTGTCACCTTCTG	-
**RPS6KA3**	TAACCGCAGAGGTCACACTCAG	CTCAGAAACTGTGGCATCCCGA	-
**OTUD7B**	GGAGGTGAAGTTACATCTGCTGC	TCAGGAGTGGACCTGGGTTCAT	-
**HMOX1**	CACTCTGGAGATGACACCTGAG	GTGTTCCTCTGTCAGCATCACC	-
**RET**	TCAGTACACGGTGGTAGCCACT	CGCCTCTTGTTTACTGCACAGG	-
**GLI2**	ACACTGTGGAGGACTGCCTACA	GGCATCTCCATGCCACTGTCAT	-
**CDH2**	CCTCCAGAGTTTACTGCCATGAC	CCACCACTGATTCTGTATGCCG	-
**IL1β**	TGGACCTTCCAGGATGAGGACA	GTTCATCTCGGAGCCTGTAGTG	-
**IL6**	TACCACTTCACAAGTCGGAGGC	CTGCAAGTGCATCATCGTTGTTC	-
**MCP-1**	GCTACAAGAGGATCACCAGCAG	GTCTGGACCCATTCCTTCTTGG	-
**TNF-α**	GGTGCCTATGTCTCAGCCTCTT	GCCATAGAACTGATGAGAGGGAG	-
**IL12β**	TTGAACTGGCGTTGGAAGCACG	CCACCTGTGAGTTCTTCAAAGGC	-
**Mstn-1**	AACCTTCCCAGGACCAGGAGAA	GGCTTCAAAATCGACCGTGAGG	-
**IL15**	GTAGGTCTCCCTAAAACAGAGGC	TCCAGGAGAAAGCAGTTCATTGC	-
**Nfix**	TCGACGACAGTGAGATGGAG	CAAACTCCTTCAGCGAGTCC	7
**Atp2a1**	GCTCATGGTCCTCAAGATCTCAC	GGGTCAGTGCCTCAGCTTTG	22
**Clcn1**	GTCCTCAGCAAGTTTATGTCC	GAATCCTCGCCAGTAATTCC	7a
**Gapdh**	ATCAACGGGAAGCCCATCAC	CTTCCACAATGCCAAAGTTGT	-

Table S6 Data of individual mice treated with CPP-PMOs antimiRs. Data on the following parameters: age, qRT-PCR, QDB, relative spleen weight, myotonia grade, and weight at different time points. Outliers are indicated in blue, in italics, and with an asterisk.

Table S7 Concentration of the CPP-PMOs in six different tissues: kidney, liver, heart, gastrocnemius, quadriceps, and brain.

		CPP-PMO concentration (pM)					
Mice	Treatment	Kidney	Liver	Heart	Gastrocnemius	Quadriceps	Brain
1990-2	9b2-218	348919.38	11986.59	1995.51	428.98	1437.67	128.86
1995-2		726042.17	23535.44	1662.31	504.97	482.70	33.45
2032-1		303314.43	16959.56	1166.04	1534.26	1344.85	18.40
1990-4	9b2KC-218	1320762.73	13391.72	184.66	788.22	358.44	1017.83
1995-4		1409556.69	5588.09	108.67	726.85	37.03	6.68
2087-1		1455590.50	12456.78	1724.69	1065.08	1605.97	4.47
2710-3	6aKC-218	807303.00	6547.20	1672.26	-	-	-
2657-3		282189.80	6702.77	-	-	-	-
2817-3		288519.00	6452.86	-	-	180.55	-
2816-3		763252.10	11820.61	220.63	3105.34	56.37	-
2713-3		163863.50	1738.96	8031.17	4126.64	3449.73	5.54
1990-3	9b2-23b	417329.90	13935.38	70.02	96.96	237.24	3.33
1995-3		596014.00	16995.86	540.73	1173.05	664.60	84.29
2032-2		445161.13	14705.39	458.72	672.99	444.65	5.89
1990-5	9b2KC-23b	404860.30	27707.42	2.97	0.01	0.82	0.87
2023-3		1672927.68	93287.30	105.40	3.32	127.62	17.78
2087-2		2213436.59	113314.70	256.63	2.34	457.08	0.01
2710-2	6aKC-23b	174345.23	9209.58	1508.23	199.58	1.72	4.02
2657-2		230939.23	8623.71	1701.45	93.86	72.30	0.83
2817-2		110334.18	4615.98	1789.14	1846.67	93.71	9.49
2816-2		239168.85	10333.80	954.01	1209.72	165.91	11.43
2713-2		16740.44	76.40	-	472.26	28.58	25.59
